# Major Role for Cellular MicroRNAs, Long Noncoding RNAs (lncRNAs), and the Epstein-Barr Virus-Encoded BART lncRNA during Tumor Growth *In Vivo*

**DOI:** 10.1128/mbio.00655-22

**Published:** 2022-04-18

**Authors:** Rachel Hood Edwards, Nancy Raab-Traub

**Affiliations:** a Lineberger Comprehensive Cancer Center, University of North Carolina at Chapel Hill, Chapel Hill, North Carolina, USA; b Department of Microbiology and Immunology, University of North Carolina at Chapel Hill, Chapel Hill, North Carolina, USA; Princeton University

**Keywords:** Epstein-Barr virus, gene expression, lncRNA, miRNA

## Abstract

This study assessed the effects of Epstein-Barr virus (EBV) and one form of virally encoded BART long noncoding RNAs (lncRNAs) on cellular expression in epithelial cells grown *in vitro* and as tumors *in vivo* determined by high-throughput RNA sequencing of mRNA and small RNAs. Hierarchical clustering based on gene expression distinguished the cell lines from the tumors and distinguished the EBV-positive tumors and the BART tumors from the EBV-negative tumors. EBV and BART expression also induced specific expression changes in cellular microRNAs (miRs) and lncRNAs. Multiple known and predicted targets of the viral miRs, the induced cellular miRs, and lncRNAs were identified in the altered gene set. The changes in expression *in vivo* indicated that the suppression of growth pathways *in vivo* reflects increased expression of cellular miRs in all tumors. In the EBV and BART tumors, many of the targets of the induced miRs were not changed and the seed sequences of the nonfunctional miRs were found to have homologous regions within the BART lncRNA. The inhibition of these miR effects on known targets suggests that these induced miRs have reduced function due to sponging by the BART lncRNA. This composite analysis identified the effects of EBV on cellular miRs and lncRNAs with a functional readout through identification of the simultaneous effects on gene expression. Major shifts in gene expression *in vivo* are likely mediated by effects on cellular noncoding RNAs. Additionally, a predicted property of the BART lncRNA is to functionally inhibit the induced cellular miRs.

## INTRODUCTION

**FIG 1:**
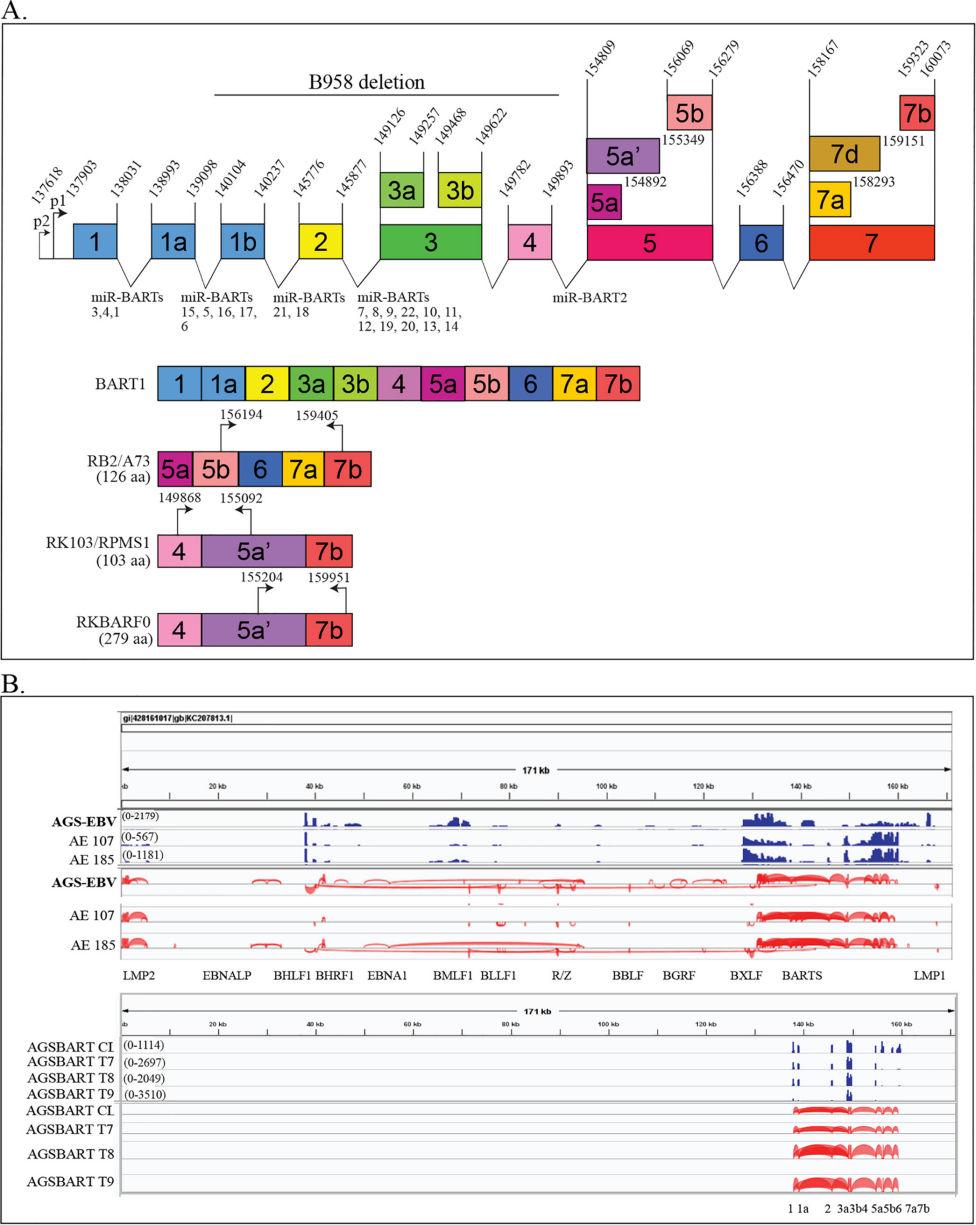
Visualization of EBV reads and splice junctions mapped to the Akata genome. Diagram of the BART locus, the BART1 clone, and the open reading frames present in previously studied BART cDNAs. (A) Diagram of the BART locus, with the locations of the exons and BART miRs. The exon structure of BART1 and the open reading frames present in the BART exons. The coordinates refer to the Akata genome. (B) Visualization of EBV reads (blue) in the AGS-EBV cell line and tumors (AE107 and AE185) and the AGS-BART cell line and tumors (AGSBART T7, T8, and T9). The numbers in parentheses are numbers of reads in the scale, with the height of the peaks indicating the number of reads. The EBV splice junctions are shown at the bottom in red, with their corresponding EBV genes indicated on the bottom axis.

Epstein-Barr virus (EBV) is a major oncogenic virus that is linked to the development of several lymphoid malignancies, including Burkitt lymphoma (BL), Hodgkin lymphoma (HL), diffuse large-B-cell lymphoma (DLBCL), primary effusion lymphomas (PEL) in coinfections with Kaposi sarcoma virus (KSHV), and central nervous system (CNS) lymphoma, and to the development of several carcinomas, including gastric carcinoma (GC) and nasopharyngeal carcinoma (NPC) (1). EBV readily infects B lymphocytes and induces continuous growth *in vitro*; however, infection of epithelial cells is much more difficult, and cell lines and xenografts have been difficult to establish from NPC and GC (1–3). Different patterns of viral gene expression are characteristic of the distinct malignancies and are also observed in cell lines and xenografts (1, 2). B lymphocytes infected *in vitro* express eight viral proteins and several noncoding RNAs and are considered to have type III latency (4). NPC is considered to have type II latency, with expression of EBNA1, latent membrane protein 1 (LMP1), latent membrane protein 2 (LMP2), the noncoding polymerase III (Pol III) EBV encoded RNAs (EBER) RNAs, and the BART (Bam-HI A rightward transcript) RNAs (2). Unique EBV expression was first identified in NPC, where sequences encoding EBNA2 and EBNA3 were not detected while abundant transcription from the BamHI A region was detected (5–7). These transcripts are encoded by a region that is deleted in the B95-8 strain of EBV and are not required for B-lymphocyte transformation (Fig. 1A) (8, 9). These abundant polyadenylated RNAs are intricately spliced, with various splices forming potential open reading frames (Fig. 1) (10–12). The BART RNAs were subsequently shown to be templates for 44 virally encoded microRNAs (miRs) produced from the introns, while the processed spliced polyadenylated BART RNAs remain nuclear and apparently function as long noncoding RNAs (lncRNAs) (Fig. 1A) (13, 14).

The AGS cell line was established from an EBV-negative gastric adenoma and is one of the few epithelial cell lines that can be infected with EBV (15). EBV infection of AGS cells results in type 1 latency, with expression of EBNA1, possibly LMP2, and the BART RNAs (4, 14, 16). EBV infection of the AGS cell line induces anchorage-independent growth and affects cellular expression (17). Analysis of cellular expression in AGS cells with and without EBV in cell lines grown *in vitro* and as tumors in immunodeficient mice in comparison with NPC xenografts revealed that expression was very different in the EBV-positive gastric cells compared to NPC, and cellular pathways involved in growth and signaling were predicted to be repressed *in vivo*. In the AGS tumors, many of the predicted upstream regulators for altered gene expression were predicted to be cellular miRs (18).

To determine the effects of EBV on expression of cellular miRs and lncRNAs and to identify the effects of a BART lncRNA *in vitro* and *in vivo*, total cellular transcription was assessed for the parental AGS cell line containing pcDNA3 (AGSpc) and the AGS-BART cell line stably expressing a single form of the highly spliced BART noncoding RNAs (BART1) in comparison with previous findings with EBV-infected AGS cells (AGS-EBV) (Fig. 1) (14, 18). The cell lines clustered together distinctly from the tumors, and the BART tumors clustered together separately from the EBV tumors. EBV had considerably greater effects on cellular expression; however, both BART and EBV tumors had changes in gene expression and altered expression of cellular lncRNAs and miRs. Many predicted and known targets of cellular lncRNAs and miRs were identified in the transcriptome sequencing (RNA-Seq) data set. These findings suggest that EBV mediates some its effects on gene expression indirectly through effects on regulatory RNAs and that the BART lncRNAs contribute to these effects.

## RESULTS

### BART tumor growth and transcription.

The AGS, AGS-EBV, and AGS-BART cell lines were inoculated subcutaneously (s.c.) as previously described (14, 18). Parental AGS cells with pcDNA3 vector (AGSpc cells) (*n* = 4) and AGS-BART cells (*n* = 3) inoculated subcutaneously produced tumors of equivalent size after 60 days *in vivo* with unremarkable histology (Fig. S1A and B). Reverse transcriptase-based PCR (RT-PCR) detected BART expression by the splices between exons 4 and 5 and exons 6 and 7 (Fig. 1). Polyadenylated selected RNA cDNA libraries and small-RNA libraries were assessed using high-throughput sequencing. The specific tumors, number of reads in the polyadenylated libraries, aligned pairs, and mapped reads to human and EBV genomes are presented in Table S1A, with all libraries producing at least 50 million reads. The percentage of reads mapping to EBV decreased dramatically in the AGS-EBV tumors compared with the cell line, decreasing from 1.5% to 0.1%. In contrast, the abundance of the BART transcript did not change in the AGS-BART tumors; the equivalent numbers of reads in cell lines and tumors represented approximately 0.01% of total reads.

10.1128/mbio.00655-22.1 FIG S1 Survival and tumor growth rates of the EBV negative AGS tumors and the BART lncRNA tumors that develop in mice, quantitative RT-PCR for the splices for the BART lncRNA transcripts, and comparison of EBV DNA levels in the AGS-EBV tumors and cell lines. (A) Kaplan-Meier survival plot of the AGSpc-injected mice and the AGS-BART-injected mice as a function of days after subcutaneous (s.c.) injection. (B) Growth rate of the tumors after s.c. injection of the cell lines measured in tumor volume and days postinjection. (C) Quantitative PCR comparing the fold change in expression of the BART lncRNA splices in the BART tumors and cell lines in reference to the AGS-EBV cell line. (D) quantitative PCR was performed in triplicate for EBV LMP2 DNA and the reference gene actin in the AGS-EBV tumors and cell lines in reference to the Raji (50 copies/cell) and Namalwa (2 copies/cell) cell lines, the C17 xenograft, and the negative-control AGS cell line and AGS T106 tumor and equal aliquots visualized on an agarose gel (18). Download FIG S1, TIF file, 0.4 MB. Copyright © 2022 Edwards and Raab-Traub. 2022 Edwards and Raab-Traub. https://creativecommons.org/licenses/by/4.0/ This content is distributed under the terms of the Creative Commons Attribution 4.0 International license.

10.1128/mbio.00655-22.2 TABLE S1 HISAT and TopHat summaries of reads to the humane (hg38) and Akata EBV genomes and small RNA reads summaries to human miRs and Akata EBV. (A) HISAT and TopHat read summaries for hg38 and Akata. (B) Small-RNA reads mapped to mature hsa-miRs. (C) Small-RNA reads mapped to Akata. Download Table S1, DOCX file, 0.06 MB. Copyright © 2022 Edwards and Raab-Traub. 2022 Edwards and Raab-Traub. https://creativecommons.org/licenses/by/4.0/ This content is distributed under the terms of the Creative Commons Attribution 4.0 International license.

As the AGS-EBV cell lines were grown without selection in nonobese diabetic/severe combined immunodeficiency (NOD) scid gamma (NSG) mice, it is possible that this decrease in RNA levels reflects loss of the EBV genome. Therefore, DNA copy number was assessed using quantitative PCR and comparison of the PCR product abundance to known DNA standards. This analysis revealed that EBV DNA copy numbers were retained in the tumors at levels equivalent to those in the cell line and that the decreased expression did not reflect loss of the viral genome (Fig. S1D).

### EBV transcription with identified splice junctions.

To determine the EBV-specific transcription, reads that did not map to the human and mouse genomes were aligned to the Akata EBV sequence using TopHat. The transcripts were assembled and quantified using Cufflinks and StringTie (Fig. 1B). In contrast to the AGS-EBV cell line, the AGS-EBV tumors (AE107 and AE185) had more restricted expression, with decreases in numbers of detected reads (Table S1 and Fig. 1). The tumors had consistent expression of the BART RNAs and LMP2 and detectable EBNA1 and BHRF1.

The AGS-BART cell line and tumors (AGSBART) had readily detectable transcription of the BART locus with expression of the specific exons 1, 1a, 2, 3a, 3b, 4, 5a, 5b, 6, and 7 contained in the BART1 clone in the cell line and tumors (Fig. 1B). The consistent expression of the BART transcripts in the absence of selection suggests that they likely contribute to the effects of EBV on cellular expression in both the EBV-infected AGS cell line and AGS-EBV tumors.

### RNA sequence analysis distinguishes AGS-EBV and BART tumors.

**FIG 2:**
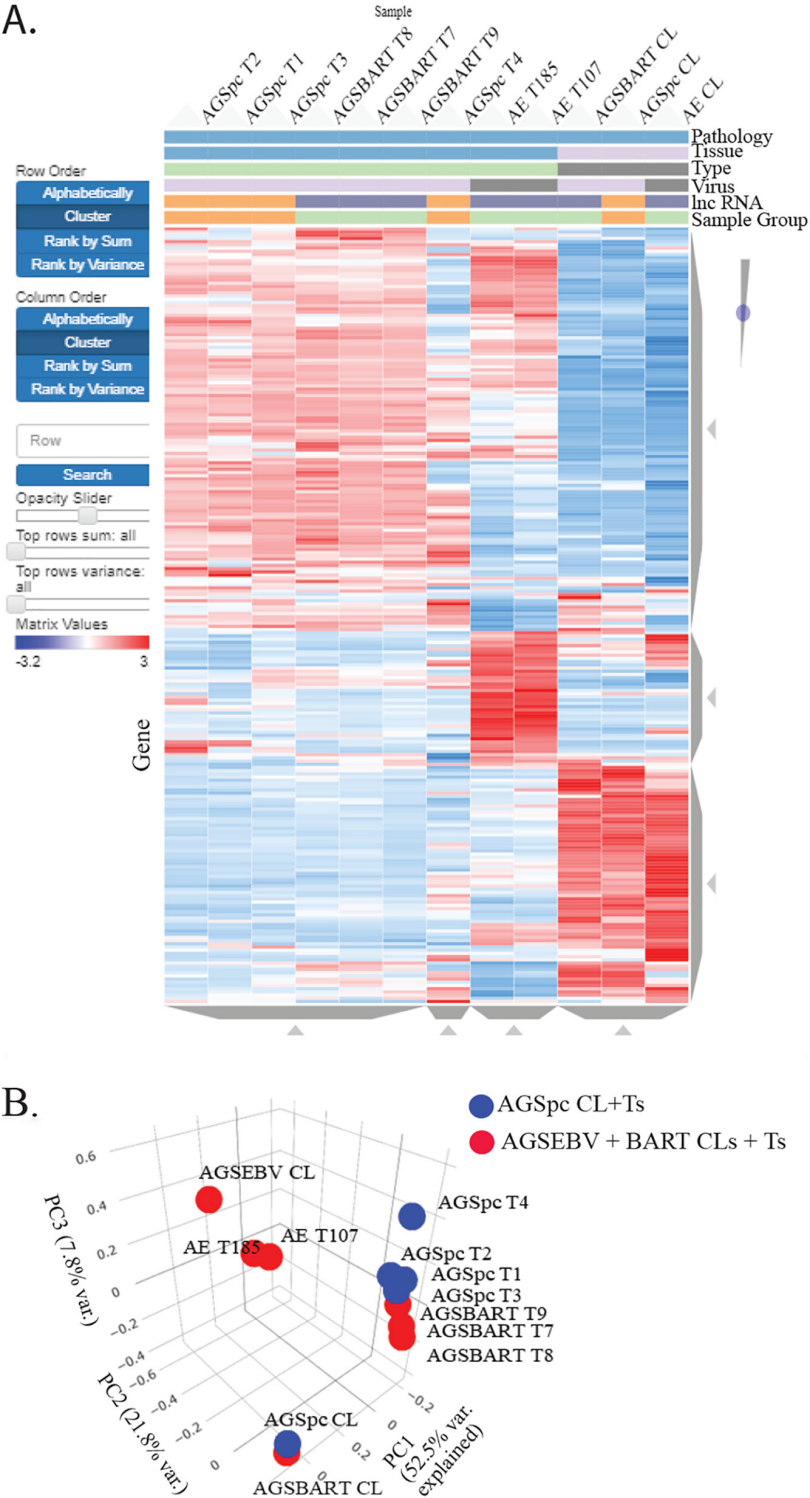
RNA-Seq analysis of the cell lines and tumors that developed in the mice following injection. (A) Hierarchical clustering heat map of gene expression of all samples versus hg38. Red denotes upregulation, and blue denotes downregulation. The samples are identified by pathology (gastric), tissue (cell line or tumor), type (s.c. tumor or cell line), virus (EBV negative or positive), lncRNA (BART lncRNA negative or positive), and sample group (BART lncRNA negative or positive). (B) Principal-component analysis based on variation between all expressed human transcripts from the AGSpc, AGS-BART, and AGS-EBV cell lines and tumors.

Aligned reads were mapped to the human Ensembl transcripts database using the Partek Genomics Suite. Partek and Biojupies were used to identify differentially expressed genes, generate hierarchical clusters, and perform principal-component analysis (PCA). Several distinct expression patterns were visible on the hierarchical heat map (Fig. 2A). The AGSpc, AGS-EBV, and AGS-BART cell lines clustered together as did the AGS-EBV tumors which clustered separately from the AGS-BART tumors. PCA analysis confirmed the clustering and revealed that the cell lines were separated in PCA1 by 52.5% and that the AGS-BART tumors were closely related to the control tumors (Fig. 2B).

**FIG 3:**
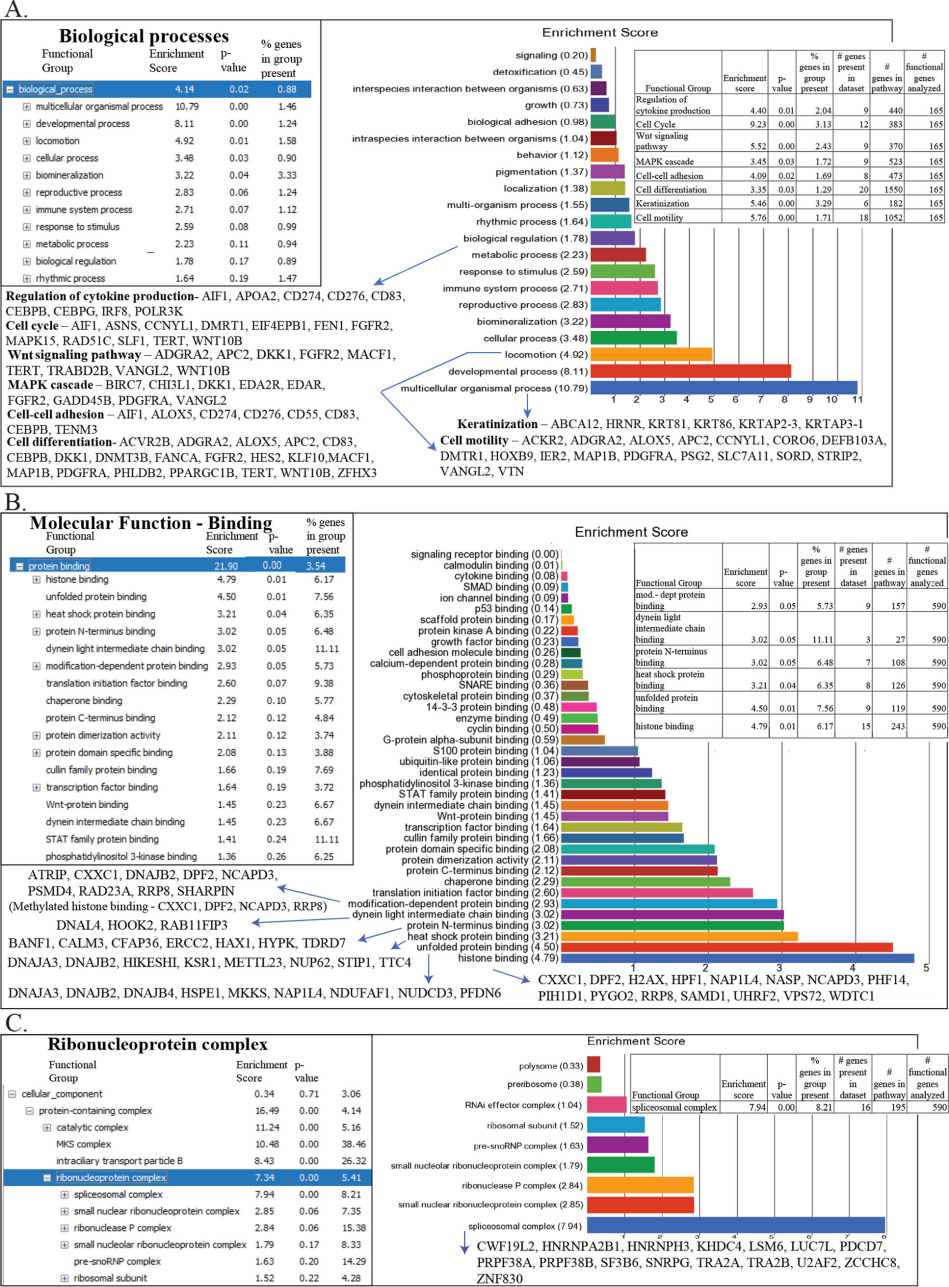
GO enrichment analysis of common differentially expressed genes changed in the same direction in the AGS-EBV and AGS-BART samples versus the AGSpc samples. (A) GO enrichment analysis of biological processes of the common differentially expressed genes of the AGS-EBV and AGS-BART cell lines versus the AGSpc cell line. The enrichment scores and *P* values of the pathways, along with gene names, are in the inset. (B) GO enrichment analysis of molecular function of protein binding of the common differentially expressed genes of the AGS-EBV and AGS-BART tumors versus the AGSpc tumors. The enrichment scores and *P* values of the pathways, along with gene names, are in the inset. (C) GO enrichment analysis of cellular components of the common differentially expressed genes of the AGS-EBV and AGS-BART tumors versus the AGSpc tumors. The enrichment scores and *P* values of the pathways, along with gene names, are in the inset.

To begin to delineate BART1 functions during EBV infection, genes changed in the same direction (*P* values and false discovery rates [FDR] of <0.05) in the AGS-EBV and BART samples versus the pc control were analyzed for Gene Ontology (GO) enrichment in Partek (Table S2). The GO analysis identified multiple biologic processes that were enriched in the AGS-EBV and BART cell lines compared to the pc control cell lines (Fig. 3A). Multicellular organismal process was enriched in the AGS-EBV and BART cell lines, and cell motility or locomotion was almost 5-fold enriched. This is interesting, as it has been shown in several studies that EBV infection of AGS cells increases motility (17, 19). Many genes associated with cell adhesion and differentiation and Wnt signaling were also enriched in the AGS-EBV and AGS-BART cell lines (Fig. 3A).

10.1128/mbio.00655-22.3 TABLE S2 Statistically significant differential expression files of genes, lncRNAs, and hsa-miRs from the comparison analyses of the AGS-EBV, BART lncRNA, and AGSpc samples (*P* < 0.05). Download Table S2, XLSX file, 2.4 MB. Copyright © 2022 Edwards and Raab-Traub. 2022 Edwards and Raab-Traub. https://creativecommons.org/licenses/by/4.0/ This content is distributed under the terms of the Creative Commons Attribution 4.0 International license.

Similar analyses of genes changed in the same direction in the AGS-EBV and BART tumors compared with the pc tumors identified protein binding as the major function, with almost 22-fold enrichment (Table S2; Fig. 3B). Histone binding was the most enriched category, followed by unfolded protein binding, and included heat shock protein binding and modification-dependent protein binding. The identification of modulation of binding to histones as a major category is interesting, considering the current understanding that lncRNAs likely impact chromatin structure and RNA transcription in part through effects on histone modifications (20). GO enrichment analysis also identified significant enrichment of genes involved in ribonucleoprotein complexes, with 16 genes in the spliceosomal complex being upregulated. This suggests that EBV, through BART expression, may also modulate splicing (Fig. 3C).

### Viral miR expression.

**FIG 4:**
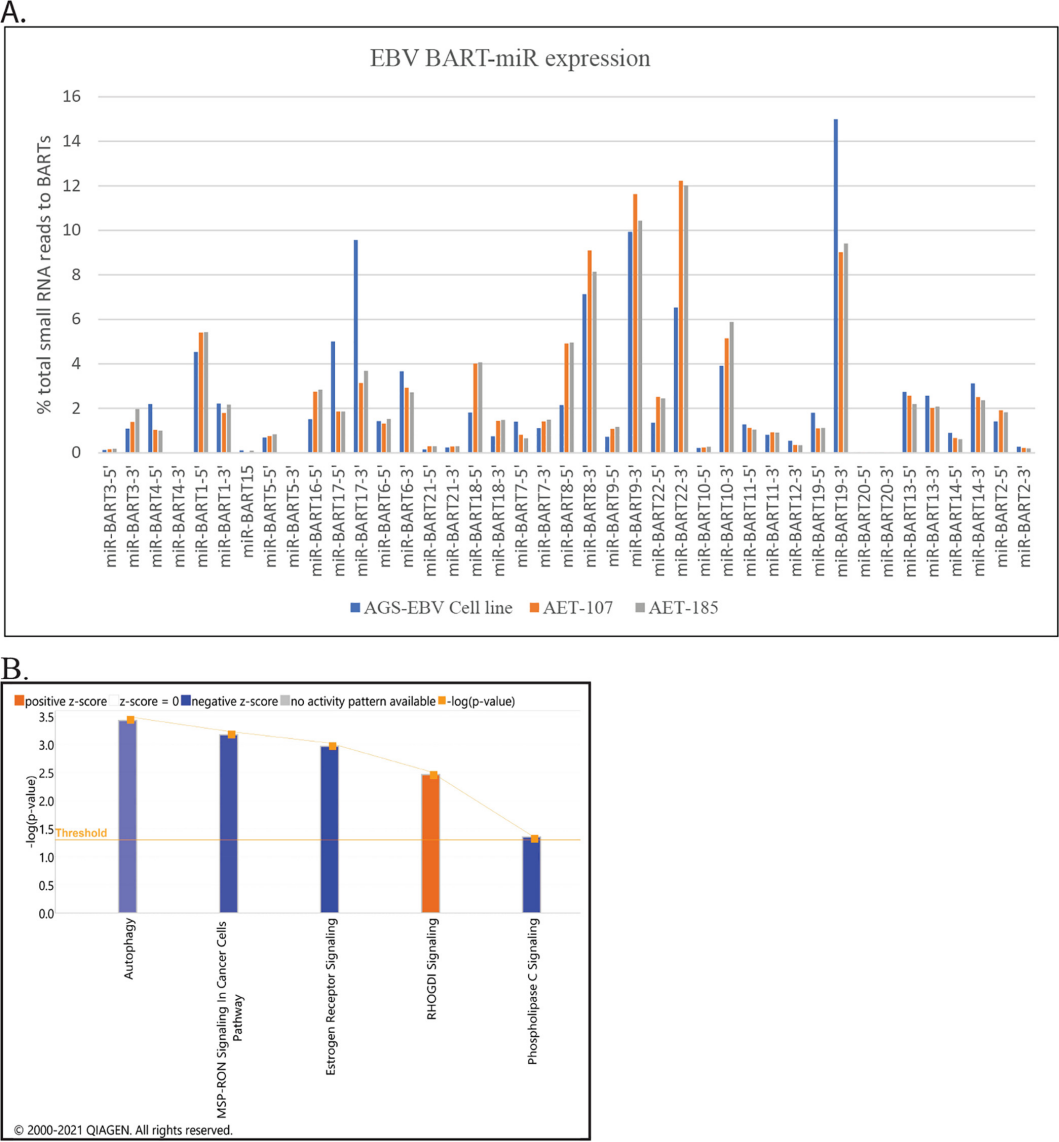
EBV BART-miR expression and canonical pathways associated with BART-miR targets with change in expression. (A) EBV BART-miR expression in the AGS-EBV cell line and tumors (AE107 and AE185) based on percentage of total small RNA reads to the BARTs. (B) Ingenuity Pathway Analysis (IPA) generated Canonical pathways associated with the BART-miR target genes that were downregulated in the AGS-EBV tumors compared to the AGSpc and AGS-BART tumors with a significant *P* value and FDR (<0.05).

Identification of viral miRs revealed that in the EBV cell line, 12.2% of the small-RNA reads mapped to the viral genome, and in the EBV tumors, 8.5 and 11.6% of all small RNAs represented viral miRs (Table S1C). miR-BART17-5p, BART17-3p, and BART19-3p were considerably more abundant in the cell lines and miR-BART18-5p, miR-BART8-5p, and BART22-3p were more abundant in the tumors based on percent small-RNA reads from the BART region (Fig. 4A).

Analysis of the sequencing data identified 68 known targets of the BART miRs with *P* values and FDR of <0.05 that had decreased expression in the AGS-EBV tumors but not in the BART or pcDNA tumors, which lack BART miR expression (Table 1). Canonical pathway analysis of the BART miR target genes indicated that autophagy, macrophage stimulating protein-recepteur d'origine nantais (MSP-RON) (linked to chronic inflammation), and estrogen receptor signaling were considerably impaired, with low z-scores, while Rho-GDI signaling was activated (Fig. 4B). It has been suggested in many studies that the EBV miRs inhibit apoptosis in cell culture systems (21, 22). The data presented here suggest that the BART miRs also impede autophagy during tumor growth. The consistent detection of considerable levels of BART miRs and identification of effects on known targets within the sequencing data set indicate that the BART miRs function in the EBV tumors and likely affect cellular expression.

**TABLE 1:** EBV-miRs and targets downregulated in AGS-EBV tumors compared to pc and BART tumors

EBV miR	Target(s)^*a*^
ebv-miR-BART3	IPO7 (−1.8/−2.3), PANK3 (−2.2/−2.7), PPARA (−2.0/−2.6), PPP3CA (−1.7/−2.3), SEC24A (−1.6/−1.8), SH3BGRL2 (−2.3/−2.3)
ebv-miR-BART4	GLCCI1 (−1.8/−2.0), MIB1 (−1.6/−1.7), PANK3 (−2.2/−2.7), RAB11FIP1 (−5.9/−5.3), RCOR1 (−1.6/−1.9), TRIM23 (−1.8/−2.3)
ebv-miR-BART1-5p	FAM120A (−1.6/−1.9), SLC39A9 (−1.6/−1.9)
ebv-miR-BART1-3p	HECTD1 (−1.8/−2.4), SEC24A (−1.6/−1.8)
ebv-miR-BART15	COBLL1 (−1.9/−2.4), DICER1 (−1.7/−2.1), HECTD1 (−1.8/−2.4), RPS6KA3 (−1.8/−1.9), TNKS2 (−1.7/−1.7)
ebv-miR-BART5	PIK3C2A (−1.7/−2.2), TNKS2 (−1.7/−1.7)
ebv-miR-BART5-1-5	ARHGAP5 (−2.4), ARHGEF12 (−1.5/−1.9), DICER (−1.7/−2.1), EHF (−2.9/−3.6), KLF13 (−1.6/−1.8), KLF3 (−2.2/−2.2), NCOA2 (−1.7/−1.9), TNKS2 (−1.7/−1.7)
ebv-miR-BART16	KLF3 (−2.2/−2.2)
ebv-miR-BART17-5p	KLHL24 (−1.8/−1.9), TRIM24 (−2.0/−2.4)
ebv-miR-BART17-3p	APOL6 (−3.1/−2.8)), ATP13A3 (−2.6/−3.4), CDKN2AIP (−1.5/−1.6)
ebv-miR-BART6-5p	DICER1 (−1.7/−2.1), SMG1 (−1.3/−1.6)
ebv-miR-BART21-5p	HECTD1 (−1.8/−2.4)
ebv-miR-BART21-3p	DIXDC1 (−2.4/−3.2), GPHN (−7.8/−10.1)
ebv-miR-BART7	MED13 (−1.6/−1.9), MEX3C (−1.5/−1.7), NCOA2 (−1.7/−1.9), PAK2 (−1.8/−2.2), SEC23A (−3.1/−3.5), SETD7 (−2.1/−2.3), TET2 (−1.8/−2.3), TNKS2 (−1.7/−1.7), ZBTB1 (−1.5/−1.8)
ebv-miR-BART18-5p	COPA (−1.5/−1.5), CREBBP (−1.7/−1.6), KLHL24 (−1.8/−1.9), PAK2 (−1.8/−2.2), RAB11FIP1 (−5.9/−5.3), SPTBN1 (−2.6/−2.9), UBR3 (−1.8/−2.1)
ebv-miR-BART8	RPS6KA3 (−1.8/−1.9), TET2 (−1.8/−2.3)
ebv-miR-BART8*	ARHGAP5 (−2.4), EHF (−2.9/−3.6), TM9SF3 (−1.7/−1.8), TMEM64 (−1.8/−2.2)
ebv-miR-BART9	SIX4 (−242.5/−307.9), FOXO3 (−1.9/−1.9), SNX18 (−2.1/−2.1)
ebv-miR-BART22	ATG2B (−1.8/−2.2), BTBD7 (−1.8/−2.1), CEP350 (−1.6/−2.1), KLF13 (−1.6/−1.8), LMAN1 (−1.5/−1.7), NFIB (−2.2/−2.4), ZBTB44 (−1.4/−1.6)
ebv-miR-BART10	FAM120A (−1.6/−1.9), FGD4 (−1.7/−1.8), MEX3C (−1.5/−1.7), SEC23A (−3.1/−3.5), SEL1L (−2.3/−2.6), UBR5 (−1.3/−1.4)
ebv-miR-BART19-5p	PSAP (−1.8/−1.8)
ebv-miR-BART19-3p	GCNT2 (−6.5/−6.1), MPP5 (−5.4/−5.9), RCOR1 (−1.6/−1.9), SNX29 (−1.4/−1.6), SPAG9 (−3.3/−4.6)
ebv-miR-BART20-3p	DICER1 (−1.7/−2.1)
ebv-miR-BART13	ATP13A3 (−2.6/−3.4)
ebv-miR-BART14	MED13 (−1.6/−1.9), PUM1 (−1.5/−1.6)
ebv-miR-BART2-5p	AHNAK (−1.9/−2.3), GLCCI1 (−1.8/−2.0), RAPH1 (−1.8/−1.9), RNF2 (−1.4/−1.7), SEC23A (−3.1/−3.5), SIPA1L1 (−2.7/−3.4), ZBTB1 (−1.5/−1.8), ZBTB44 (−1.4/−1.6)

a Fold change in expression is shown in parentheses (AGS-EBV versus pc/AGS-EBV versus BART), with a *P* value and an FDR of <0.05.

### Cellular miR expression.

**FIG 5:**
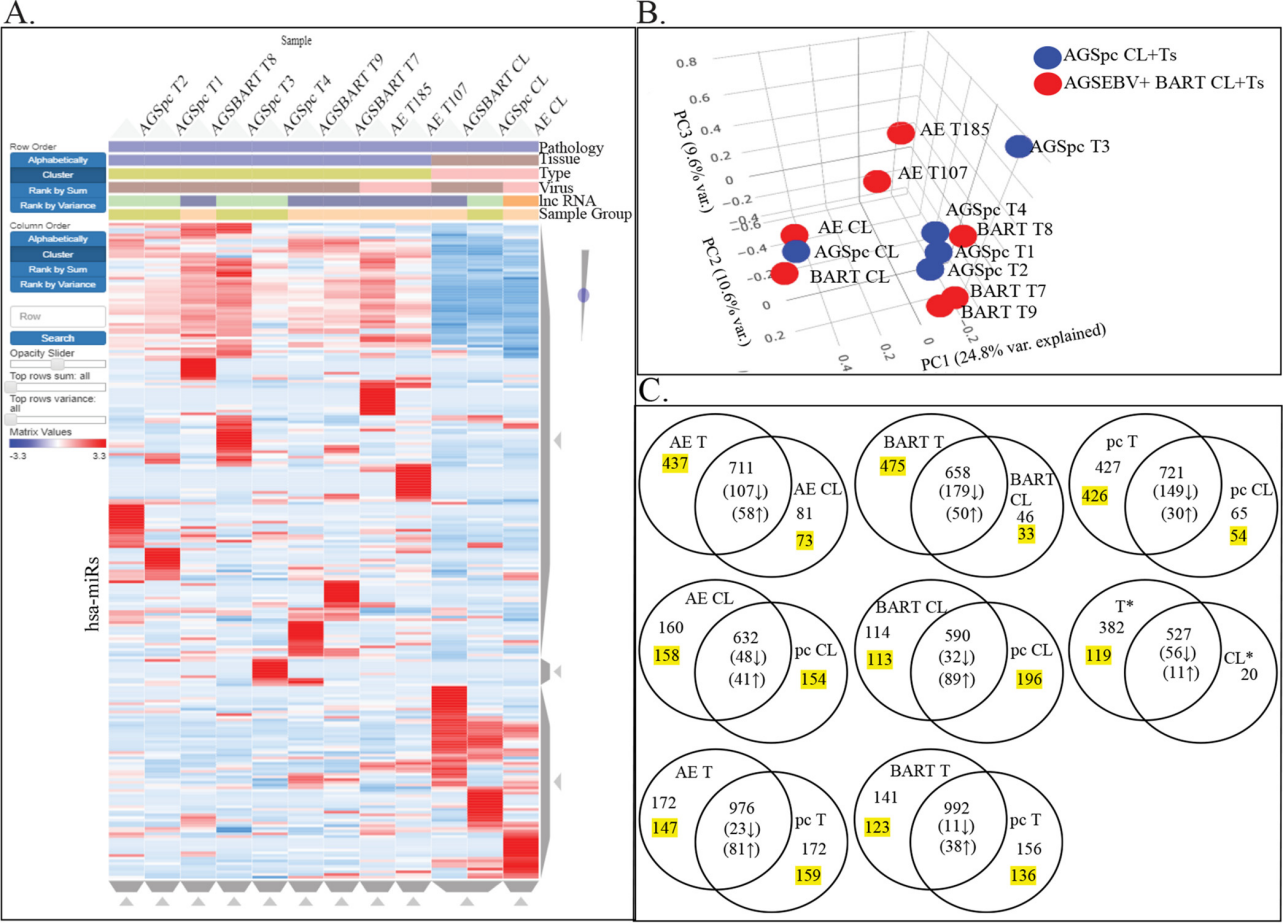
miRNA-Seq analysis of the small reads of the cell lines and tumors that develop in the mice following injection. (A) Hierarchical clustering heat map of hsa-miR expression of all samples versus human miRs. Red denotes upregulation, and blue denotes downregulation. The samples are identified by pathology (gastric), tissue (cell line or tumor), type (s.c. tumor or cell line), virus (EBV negative or positive), lncRNA (BART lncRNA negative or positive), and sample group (BART lncRNA negative or positive). (B) Principal-component analysis based on variation between all expressed human miRs from the AGSpc, AGS-BART (BART), and AGS-EBV (AE) cell lines and tumors. (C) Venn diagrams based on hsa-miR expression in the AGSpc, AGS-EBV, and AGS-BART cell lines and tumors. Expression is based on hsa-miR reads of >0.5 reads per kilobase per million (RPKM); numbers in parentheses are numbers of hsa-miRs with differential expression with significant *P* values (<0.05), and highlighted numbers reflect hsa-miRs with 0 reads in the compared sample. *, hsa-miR expressed in all tumors and/or cell lines.

We showed previously that in tumors *in vivo*, the predicted upstream regulators of genes with altered expression are cellular miRs (18). To identify the effects on expression of cellular miRs, small-RNA libraries were prepared from size-selected RNA from all samples and sequenced. Clustergram analysis indicated that the cell lines grouped together, with striking regions of decreased or activated expression that differed between the EBV, BART, and pc cell lines. The EBV tumors clustered together, as did the BART tumors, with the exception of BART T8, which was more closely related to the pc control tumors (Fig. 5A). PCA confirmed the clustering of the cell lines, which were separated from the tumors by approximately 25% of the variance (Fig. 5B). Identification of hsa-miR transcripts revealed that in all tumors, the percentage of miRs with reads increased considerably in comparison with that in the matching cell line (Table S1B). This increase was statistically significant, with a *P* value of 0.0026 (Table S1B).

Venn analysis of differential expression revealed that the AGS-EBV tumors had 437 miRs with unique expression, compared to 81 unique miRs in the EBV cell line (Fig. 5C). The BART tumor had 475 miRs uniquely expressed, while the cell line had 46. The control tumors had 427 induced miRs, while the cell line had 65. Significant differences in the number of miRs were not identified in comparisons of EBV or BART cell lines to pc control cell lines or EBV or of BART tumors to pc tumors. More than one-third of all the hsa-miRs that were identified were detected only in the tumors, although the majority of these miRs were less than 0.001% of total host miR reads per million (rpm).

To identify potentially functional miRs that altered gene expression, cellular miRs with changed expression were analyzed in conjunction with affected gene expression using Ingenuity Pathway Analysis (IPA). Identification of hsa-miRs with significant fold changes (*P* < 0.05) that were predicted to be upstream regulators identified multiple targets that were correspondingly up- or downregulated in the sequencing data set (Table 2). In particular, miRs with higher expression in tumors than cell lines had major effects in decreasing expression of the known miR targets in the data set, with 94% of the targets downregulated in the pc tumors, 89% in the BART tumors, and 81% in the EBV tumors (Table 2; Table S3). Several of the cellular miRs were significantly changed in common in all tumor types. The hsa-miRs 148a/b-3p, 26a/b-5p, 3074-5p, 30d-5p, and 424-5p were upregulated in all the tumors, with the majority of their targets being downregulated in the gene set. These miRs were predicted by IPA to be upstream regulators of the differentially expressed gene set (Table 2; Table S3). The hsa-miRs 106b-5p, 107, and 503-5p were specifically upregulated in the AGS-EBV tumors compared to the cell line and had numerous targets downregulated (Table 2; Table S3A). Identification of cellular miRs with decreased expression in the tumors compared to the cell lines indicated less dramatic effects on expression of known targets in the data set, with only a subset of targets affected (Table 2). In contrast, in comparisons of EBV or BART tumors to pc tumors, the miRs that had decreased expression had significant effects on targets in the gene set, with increased expression of most targets. Surprisingly, although many miRs had increased expression in the EBV tumors compared to the pc tumors, most of their targets were not affected; only 14% of the targets of the upregulated miRs were downregulated. Similarly, analysis of the miRs that were upregulated in the BART tumor compared to the pc tumor also indicated that predicted targets were not affected, with only 3% being downregulated. For example, the miR let-7b-5p was upregulated 1.5-fold in the BART tumor, and only two of the possible 175 targets were downregulated (Table 2; Table S3E). This lack of effect was more specific to comparisons of the EBV and BART tumors to the pc tumors and was not as apparent in comparisons of EBV or BART cell lines to the pc cell line, where 63% and 44% of their respective targets were downregulated (Table 2). This suggests that in the EBV and BART tumors, many of the cellular miRs with increased expression do not function. It is possible that these miRs are sponged by lncRNAs whose expression is also changed. Additionally, the BART lncRNA may potentially inactivate cellular miRs by sponging due to complementary sequences to the miR seed sequences.

**TABLE 2:**
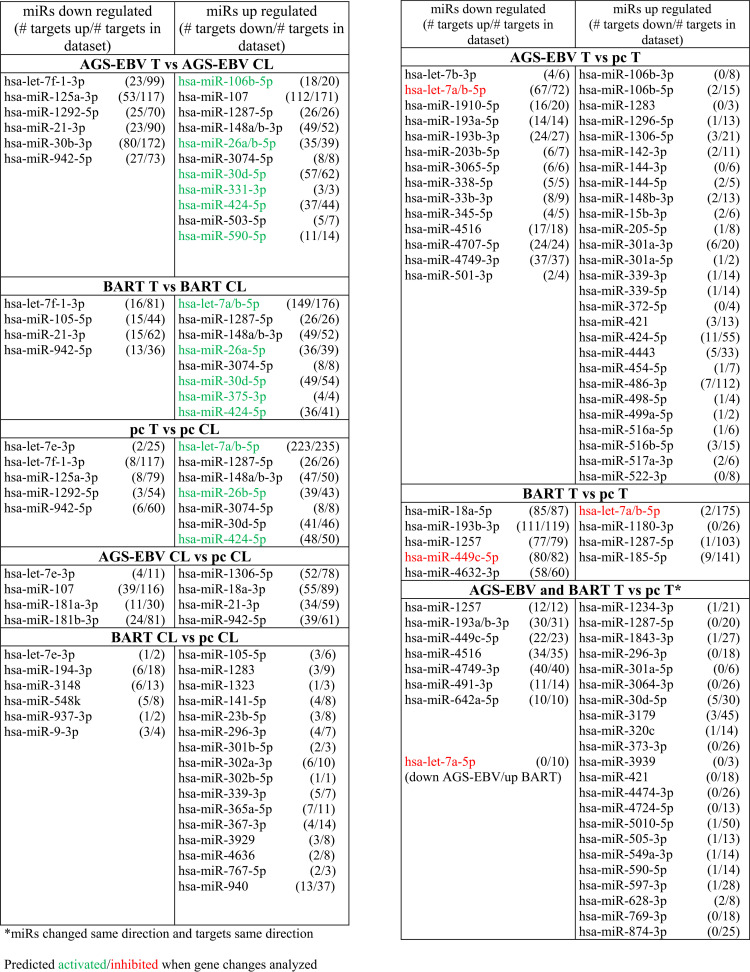
hsa-miR regulators

10.1128/mbio.00655-22.4 TABLE S3 Predicted hsa-miR upstream regulators and target changes. (A) AGS-EBV tumor versus AGS-EBV cell line; (B) BART tumor versus BART cell line; (C) pc tumor versus pc cell line; (D) AGS-EBV tumor versus pc tumor; (E) BART tumor versus pc tumor; (F) common AGS-EBV and BART tumor versus pc tumor; (G) all tumors versus cell lines. Download Table S3, DOCX file, 0.1 MB. Copyright © 2022 Edwards and Raab-Traub. 2022 Edwards and Raab-Traub. https://creativecommons.org/licenses/by/4.0/ This content is distributed under the terms of the Creative Commons Attribution 4.0 International license.

**FIG 6:**
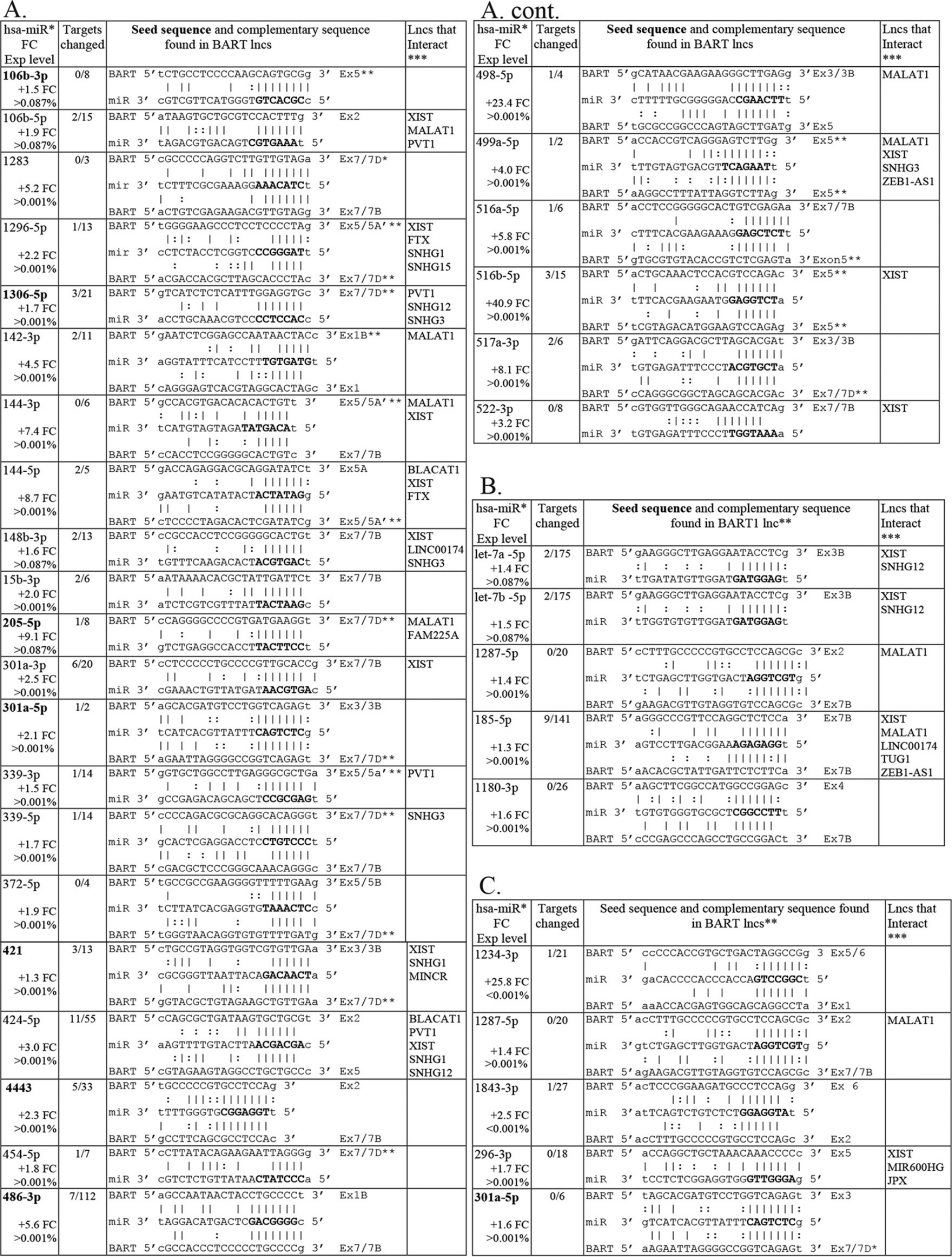

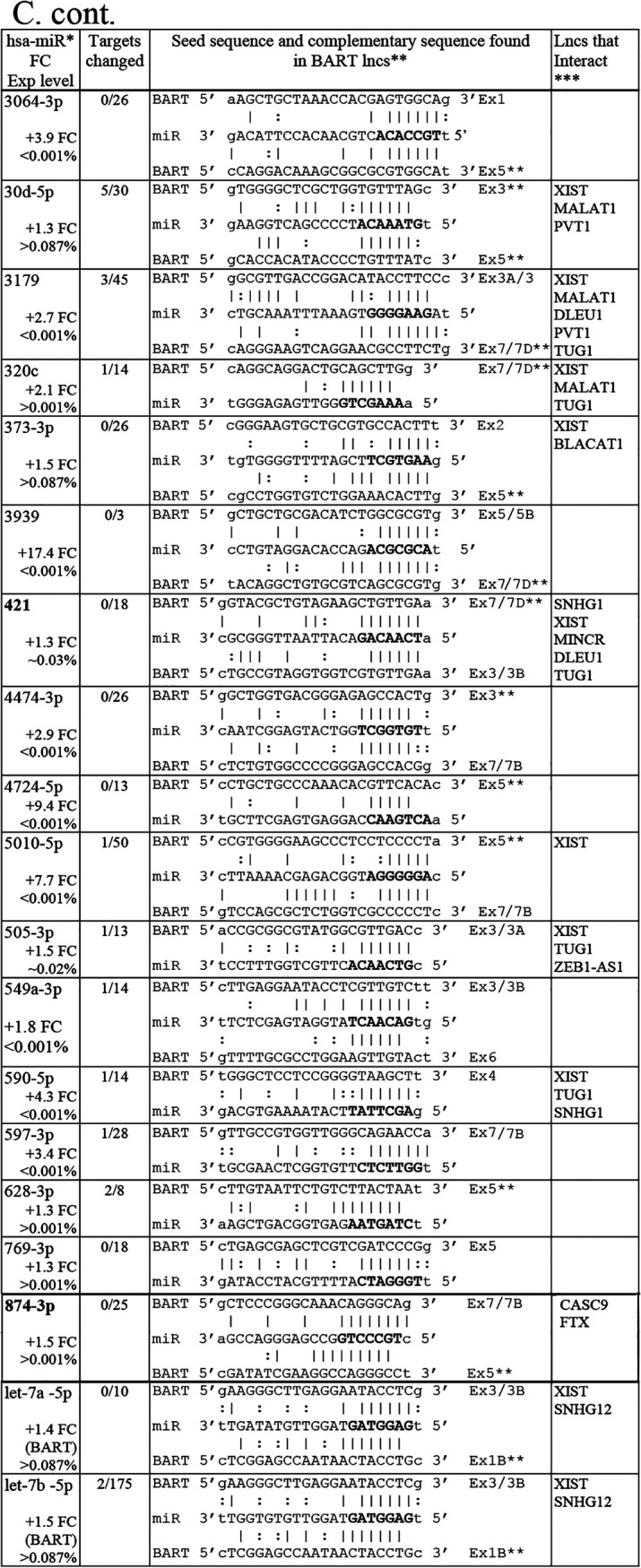
Cellular miRs upregulated in AGS-EBV and AGS-BART tumors versus pc tumors with targets unchanged and complementary seed sequence found in BART lncRNA exons. hsa-miRs upregulated in the AGS-EBV and BART lncRNA tumors versus the pc tumors with fold change (FC) in expression and expression level indicated (*), the number of targets changed out of those in the data set of significantly changed genes (*P* < 0.05), and the complementary sequences to the miR found in the BART lncRNA exons with the seed sequence in boldface. miRs having a perfect match to the seed sequence in the BART lncRNAs are in boldface. **, The BART1 lncRNA consists of exons 1-1A-2-3A-3B-4-5A-5B-6-7A-7B, so not all potential complementary sequences are encoded in the BART1 lncRNA. ***, The lncRNAs listed are those shown to interact with the miRs based on STARBASEv3.0. (A) miRs upregulated (*P* < 0.05) in the AGS-EBV tumors versus the pc tumors with targets unchanged and complementary seed sequence present in the BART1 lncRNA exons. (B) miRs upregulated in the BART1 tumors (*P* < 0.05) versus the pc tumors with targets unchanged and complementary seed sequence found in BART1 lncRNA exons. (C) Common miRs upregulated (*P* < 0.05) in the AGS-EBV and BART1 tumors versus the pc tumors with targets unchanged and complementary seed sequence found in the BART lncRNA exons.

To identify potential BART lncRNA effects on cellular miR function, the sequences of the hsa-miRs that had increased expression in the EBV and BART tumors yet did not affect expression of known cellular targets were searched for homology in the BART lncRNAs (Fig. 6). For each miR, a sequence with considerable complementary homology to the miR seed sequence was identified within the BART lncRNA. The homology and the location within the BART RNA are indicated (Fig. 6). The hsa-miRs 106b-3p, 1306-5p, 205-5p, 301a-5p, 421, 4443, 486-3p, and 874-3p all had perfect matches to their seed sequence in the BART lncRNA exons, and only 20 of their 222 known targets were downregulated (Fig. 6A and C). Also of note is the strong homology to the miR let7a/b-5p identified in the BART lncRNAs (Fig. 6B and C). miR let-7a/b is highly expressed in all the tumors and upregulated in the BART tumors compared to the pc tumors, yet its targets are not downregulated. Strong homology and in many cases multiple sites of homology in the BART lncRNA, were detected for all the hsa-miRs that were upregulated in the AGS-EBV and BART tumors and that lacked expression changes in their targets (Fig. 6).

These analyses reveal that during growth *in vivo* when many of the cellular growth and signaling pathways have decreased activity, many cellular miRs have increased expression with clear effects on target expression. However, in the EBV and BART tumors, a subset of the induced cellular miRs have complementary homology to their seed sequence in the BART lncRNA and do not affect their target expression. These data indicate that cellular miRs are major effectors of changes in expression *in vivo* and *in vitro*. Additionally, the data reveal that the EBV lncRNA modulates the effects of the cellular miRs induced *in vivo* and likely decreases the inhibitory effects of these miRs on cellular expression.

**FIG 7:**
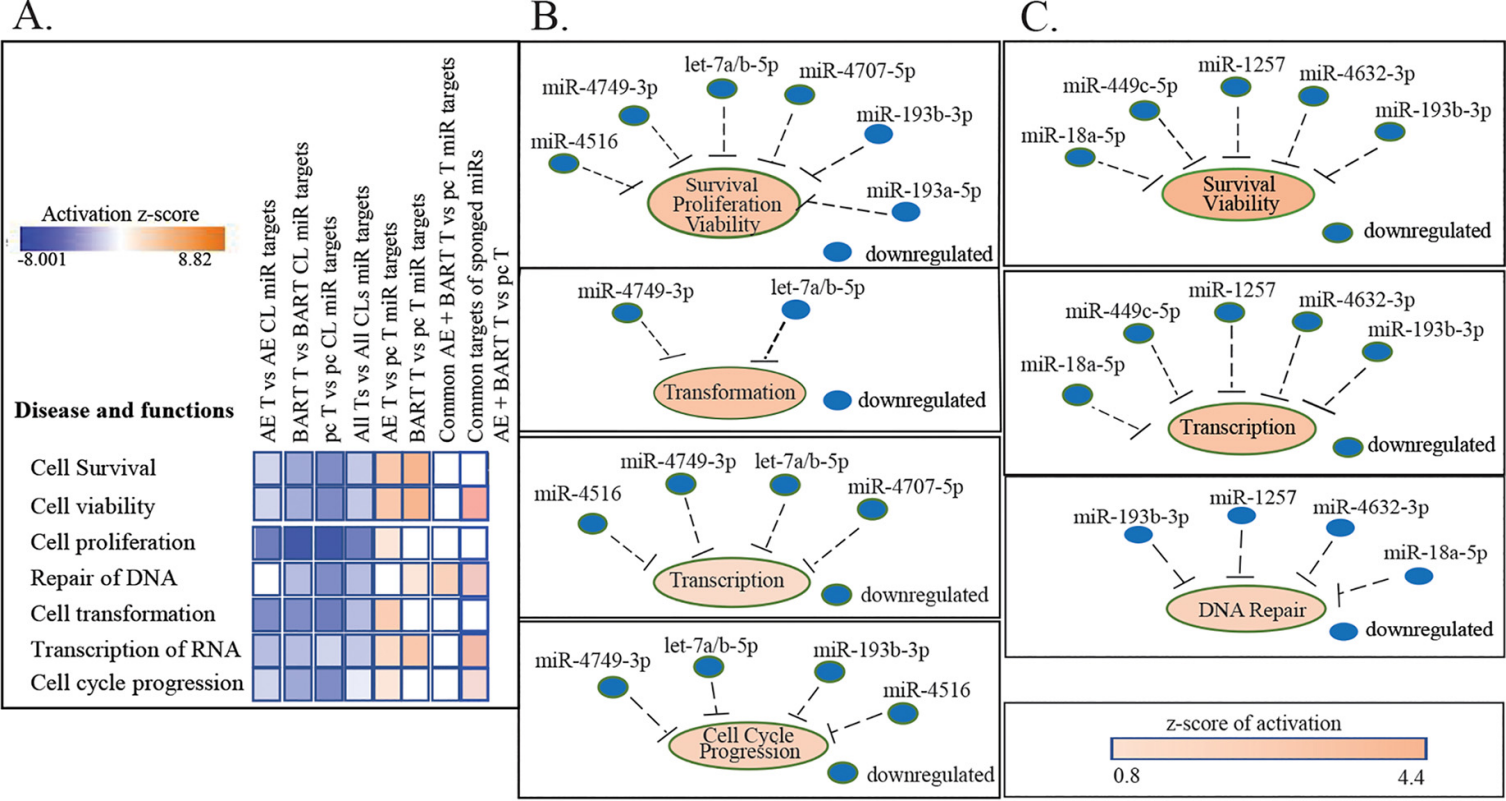
Differentially expressed hsa-miRs and their target changes contribution to tumor development. (A) Analysis of disease and functions resulting from the hsa-miR target changes in tumors compared to their cell line and the AGS-EBV and AGS-BART samples compared to the AGSpc samples (*P* < 0.05). Blue denotes a negative z-score (inhibition), and red denotes a positive z-score (activation). (B) hsa-miRs with target changes associated with increased activation of survival, viability, transformation, transcription, proliferation, and cell cycle progression in the AGS-EBV tumors compared to the AGSpc tumors (*P* < 0.05). (C) hsa-miRs with target changes associated with increased activation of survival, viability, transcription, and DNA repair in the AGS-BART tumors compared to the AGSpc tumors (*P* < 0.05).

To identify the effects of these target changes, IPA disease and function analysis of the genes affected by the miRs identified functions that are inhibited, with negative z-scores, and those that are activated, with positive z-scores. Many pathways indicative of highly proliferative growth, such as survival, viability, proliferation, and transformation, had greatly decreased activity in the tumors compared to the cell lines, confirming our previous findings that most signaling pathways are repressed in the tumors (Fig. 7A) (18). In contrast, comparison of the EBV or BART tumors to the pc tumors indicated that these processes, including survival, viability, and transcription, were activated in the EBV and BART tumors (Fig. 7A). Identification of the specific miRs that contribute to these processes revealed that those that have decreased expression would be inhibitory to the property (Fig. 7B). The decreased expression of the miRs 4516, 4749-3p, let7a/b-5p, 4707-5p, 193b-3p, and 193a-5p in the EBV tumors and subsequent increase in expression of their targets would enhance the survival, viability, transformation, and proliferation of the EBV tumors (Fig. 7B; Table S3D). HRAS, a target of the upregulated miR 1306-5p, and AKT2, a target of miR 4443, were not downregulated, possibly due to the complete matches to the seed sequences of the regulating miRs in the BART lncRNA (Fig. 6A; Table S3D). The increased levels of HRAS and AKT would also contribute to the more proliferative state of the EBV tumors (Table S3D). In the BART tumors, the decreased expression of the miRs 18a-5p, 449c-5p, 1257, 4632-3p, and 193b-3p with the resulting increase in expression of their targets would enhance survival, viability, and RNA transcription (Fig. 7C; Table S3E). The targets of the miRs that are possibly sponged by BART lncRNAs and were not downregulated in the EBV and BART tumors were analyzed separately, and the increased expression of the targeted genes would also lead to enhancement of viability, DNA repair, transcription, and cell cycle progression (Fig. 7A; Table S3F). This suggests that the repression of active growth *in vivo* is somewhat alleviated by EBV infection and in part by the BART lncRNA.

### Effects on cellular lncRNA expression.

**FIG 8:**
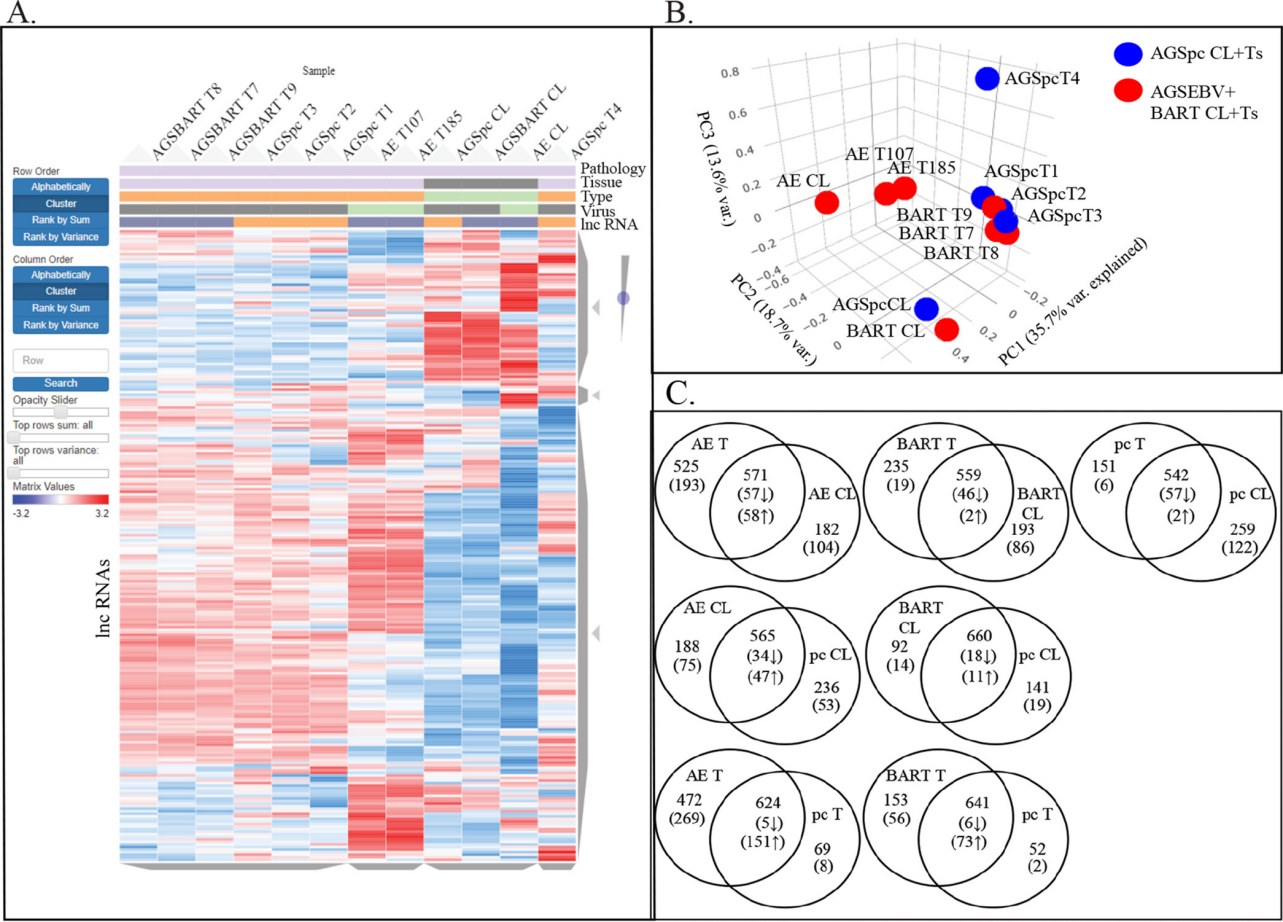
lncRNA Analysis of the cell lines and tumors that develop in the mice. (A) Hierarchical clustering heat map of lncRNA expression of all samples. Red denotes upregulation, and blue denotes downregulation. The samples are identified by pathology (gastric), tissue (cell line or tumor), type (s.c. tumor or cell line), virus (EBV negative or positive), and lncRNA (BART lncRNA negative or positive). (B) Principal-component analysis based on variation between all expressed lncRNAs from the AGSpc, AGS-BART, and AGS-EBV cell lines and tumors. (C) Venn diagrams based on lncRNA expression in the AGSpc, AGS-EBV, and AGS-BART1 cell lines and tumors. Expression is based on lncRNA reads of >0.5 RPKM; numbers in parentheses are numbers of lncRNAs showing differential expression with significant *P* values (*P* < 0.05).

To identify effects of EBV *in vivo* on lncRNA expression, lncRNAs were identified within the RNA sequence data using the lncRNA disease 2 database and the LNC2CANCER database. Heat map analysis revealed that the lncRNAs distinguished all cell lines which grouped together and each of the three tumor types (Fig. 8A). The cell lines had major regions with decreased lncRNA expression and several regions with increased lncRNA transcription. The tumors had almost a reverse pattern of expression, with large regions representing increased expression. PCA analysis also revealed the distinct groups, with 35.7% of the variance distinguishing the cell lines from the tumors (Fig. 8B). PCA analysis also confirmed the close relationship of the BART tumors to the pc tumors.

Analysis of differential expression did not reveal striking differences between lncRNA expression in the BART and pc tumors compared to the matched cell lines or in comparisons of cell line to cell line, while the EBV tumors had more lncRNAs expressed than their corresponding cell lines (Fig. 8C). Comparison of the EBV tumors to the pc tumors revealed a considerable increase in lncRNA transcription. The EBV tumors had 472 unique lncRNAs, compared to 69 unique lncRNAs in the pc tumors, and of the 624 lncRNAs expressed in both tumor types, 151 had increased expression in the EBV tumors (Fig. 8C). The BART tumors had slightly elevated unique lncRNAs, 153 compared to 52 in pc tumors, and of the 641 detected in both, 73 had increased expression in the BART tumors (Fig. 8C). These findings indicate that EBV infection increases lncRNA expression and that the BART RNA also affects cellular lncRNA expression levels.

Differential expression of the cellular lncRNAs was determined, and those that had previously described known targets and had increased expression in the EBV tumors were identified, with many increased more than 2-fold (Table 3). The rank in the list of lncRNAs in the EBV, BART, and pc tumors is indicated (Table 3). The fold change of the proposed lncRNA targets in the tumors reveals that many are affected in the EBV tumor RNA-Seq data. Expression of the lncRNA BCYRN1 was increased 6.2-fold, and it was ranked 82 of all of the detected lncRNAs in the EBV tumors and ranked 465 in the pc tumors (Table 3). BCYRN1 potentially binds the miR 939-3p and affects the levels of the miR target, HDAC11 (23). In the EBV tumors, HDAC11 was increased 2.3-fold (Table 3). The BLACAT1 lncRNA, which is increased 3.7-fold, has been suggested to induce EZH2, a component of the PRC complex, which was increased 1.7-fold, resulting in increased H3K27 trimethylation and decreased expression of the cyclin-dependent kinase inhibitor CDKN1C, decreased 2.3-fold in the gene expression data (24). Several of the induced lncRNAs, such as MALAT1, PVT1, and XIST, have also been shown to have specific properties in gastric cancer and were also identified as upregulated in NPC (Table S4B) (25–27).

**TABLE 3:** LncRNAs upregulated in AGS-EBV tumors

lncRNA	FC^*a*^	Rank^*b*^	Mechanism^*c*^	Biological function	Reference
BCYRN1^*d*^	+6.2; +2.5	82/465; 181/465	miR-939-3p/HDAC11 (**+2.3**) axis	Proliferation	23
			MYC upregulates BCYRN1	Invasion, migration	29
BLACAT1	+3.7	69/222	EZH2 (**+1.7**) induced H3K27me3 epigenetic silencing, CDKNIC (−2.3)	Proliferation, migration	24
			miR-16 (424-5p) sponge, CCND1, MYC	Transformation, invasion	28
DBH-AS1^*e*^	+4.0	805/1256	miR-138/FAK/Src/ERK pathway	Proliferation, apoptosis	30
			Upregulation of PI3K/AKT pathway	Proliferation, migration	31
FAM225A^*e*^	+2.8	965/1182	miR-590-3p/miR-1275/FAK/PI3K/Akt	Proliferation, metastasis	32
FIRRE^*e*^	+3.5	659/1113	MYC/Wnt/β-catenin	Proliferation	33
FTX^*e*^	+2.9	668/1006	miR-144/ZFX (+1.3) axis	Proliferation, invasion	34
			Positive regulation of XIST		35
JPX^*d*^	+2.2; +1.5	344/481; 366/481	miR-5195-3p/VEGFA (+1.7) axis	Proliferation, invasion	36
			XIST (**+3.3**)		37
			PI3K	Proliferation, invasion	38
LINC00174^*d*^^,^^*e*^	+2.8; +1.6	532/842; 651/842	miR-3127-5p/ E2F7 axis (+1.2)	Proliferation, migration	39
LINC00659^*d*^	+4.3; +1.8	115/411; 242/411	IQGAP3 (+1.6)	Migration, invasion	40
LINC00680	+1.8	184/223	miR-410-3p/HMGB1 axis (+1.2)	Proliferation	41
LINC00888	+2.1	191/285	Sponging miR-34a	Proliferation, migration	42
LINC01138^*e*^	+3.0	712/1105	miR-375/SP1 axis (+1.2)	Proliferation	43
MALAT1	+2.3	3/3	Vasculogenic mimicry via VE-cadherin/β-catenin complex	Metastasis	44
			ERK/MMP and FAK/paxillin signaling path		25
			miR-124/Capn4 (CAPNS1) axis in NPC		45
			PI3K/Akt pathway in gastric cancer	Proliferation, invasion	46
			HIF1A (stabilization)	Proliferation, invasion	47
MINCR	+3.6	254/616	miR-26a-5p/EZH2 axis (**+1.7**)	Proliferation, invasion	48
			miR-126/SLC7A5 axis (**+1.7**)	Proliferation	49
MRPL23-AS1^*d*^^,^^*e*^	+3.4; +2.1	471/904; 543/904	miR-30b/MYH9/Wnt/β-catenin	Metastasis	71
PVT1^*e*^	+2.5	888/1068	miR-149-5p/FOXM1 axis (**+1.7**) in GC	Proliferation, invasion	50
			Upregulated by FOXM1	Proliferation, invasion	51
			STAT3/VEGFA axis (+1.7) in GC	Angiogenesis	52
			Vasculogenic mimicry STAT3/Slug axis	Proliferation	53
			KAT2A (**+2.2**)/HIF1A in NPC	Proliferation	26
			Upregulates CCND1, MYC	Proliferation, migration	54
			Sponges miR-16/VEGFA (+1.7)	Proliferation, invasion	55
			Sponges let-7 (circPVT1)	Proliferation	56
SNHG1	+2.6	19/40	miR-154-5p/EZH2 (**+1.7**)		
			PRC2/KLF2 (−2.8)/CDKN2B (−1.2)	Proliferation	72
			DNMT1 (**+1.4**) in GC	Proliferation	57
			Upregulation MYC, AKT	Proliferation, invasion	58
			Sponges miR-16 (424-5p)	Transformation, invasion	59
SNHG12	+2.1	57/96	Activating PI3K/AKT pathway in GC	Proliferation	60
			Notch signaling in NPC	Proliferation, metastasis	61
SNHG15	+2.0	87/141	EZH2 (**+1.7**) mediated H3K27me3		
			KLF2 (−2.8)/CDKN2B(−1.2)	Proliferation	62
SNHG3	+2.6	27/68	miR-3619-5p/ARL2 axis (+1.3)	Proliferation, viability	63
			miR-326/ITGA5 (**+1.9**) Vav2/Rac1 (+1.5) signaling pathway	Viability, invasion, migration	64
TPT1-AS1^*e*^	+2.2	582/739	TPT1-AS1/NF90/VEGFA(+1.7) signaling	Angiogenesis, metastasis	65
XIST^*f*^	+3.3; +1.6	9/20; 17/20	miR-185/TGFB1 (+1.5) axis	Proliferation	66
			miR-101/EZH2 (**+1.7**) axis	Proliferation, migration	67
			Sponging miR-let-7b	Proliferation, migration	68
			miR-93-5p/HIF1A axis		69
ZEB1-AS1^*d*^^,^^*e*^	+1.8; +1.5	846/857; 720/857	Sponging miR-335-5p in GC	Proliferation, invasion	70

a Fold change in expression (with a *P* value and FDR of <0.05) relative to AGSpc tumors. The AGS-EBV fold change is listed first when the lncRNA is also upregulated in AGS-BART.

b Rank in expression compared to AGSpc tumors (values of >693 have <0.5 RPKM in AGSpc tumors). The numerator reflects the rank in AE or BART tumors, and the denominator reflects the rank in pc tumors. The AGS-EBV ranking is listed first when the lncRNA is also upregulated in BART.

c Fold change in target expression relative to AGSpc tumors. Boldface indicates results with *P* values and FDR of <0.05.

d Also upregulated in AGS-BART1 tumors.

e Not expressed in pc tumors (<0.5 RPKM).

f Also upregulated in BART tumors, but the FDR was >0.05.

10.1128/mbio.00655-22.5 TABLE S4 Statistically significant (*P* < 0.05) differentially expressed lncRNAs from the comparison analyses of the AGS-EBV, BART lncRNA, and AGSpc samples. (A) Differentially expressed lncRNAs between the tumors and corresponding cell line; (B) differentially expressed lncRNAs between the AGS-EBV and AGS-BART cell lines and the pc control cell line; (C) differentially expressed lncRNAs between the AGS-EBV and BART tumors and the pc control tumors. Download Table S4, DOCX file, 0.03 MB. Copyright © 2022 Edwards and Raab-Traub. 2022 Edwards and Raab-Traub. https://creativecommons.org/licenses/by/4.0/ This content is distributed under the terms of the Creative Commons Attribution 4.0 International license.

**FIG 9:**
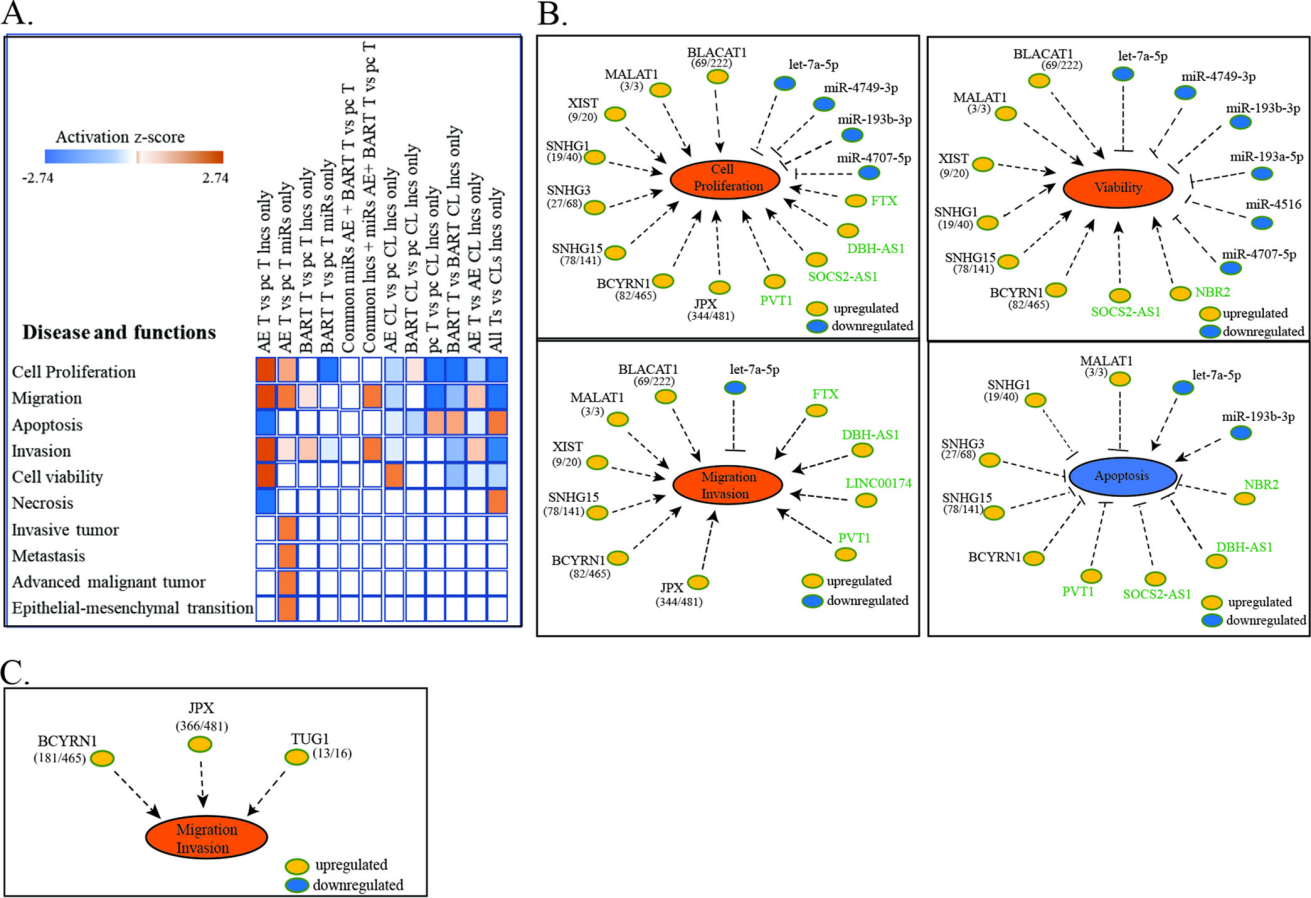
Differentially expressed lncRNAs and hsa-miRs and their contribution to tumor development. (A) Comparison analysis of disease and functions resulting from the lncRNAs and hsa-miRs in the AGS-EBV and AGS-BART samples compared to the AGSpc samples (*P* < 0.05). Blue denotes a negative z-score (inhibition), and red denotes a positive z-score (activation). (B) lncRNAs and hsa-miRs associated with increased activation of proliferation, viability, migration and invasion, and decreased apoptosis in the AGS-EBV tumors compared to the AGSpc tumors. The numbers in parentheses reflect the rank in expression of the lncRNA in the AGS-EBV tumors compared to the pc tumors (*P* < 0.05). The lncRNAs in green font reflect those expressed only in the AGS-EBV tumor and not the pc tumors. (C) lncRNAs associated with increased migration and invasion in the AGS-BART tumors compared to the AGSpc tumors (*P* < 0.05). The numbers in parentheses reflect the rank in expression of the lncRNA in the AGS-BART tumors compared to the pc tumors.

In most cases, the attributed biologic functions associated with these lncRNAs are proliferation, viability, invasion, and metastasis (Table 3) (25, 28–72). These biologic properties are also targets of the affected cellular miRs (Fig. 7 and 9). Significantly affected lncRNAs with increased expression, including BLACAT1, DBH-AS1, FTX, MALAT1, PVT1, and XIST were identified by IPA to positively regulate proliferation, migration, and invasion (Fig. 9). The miRs with decreased expression, including let7a-5p, 4749, 193b-3p, and 4707, inhibit proliferation and migration such that their decreased function complements the induced lncRNAs (Fig. 9).

IPA analysis of disease and functions confirms that proliferation and migration are decreased in comparisons of the pc and BART tumors to their cell lines while apoptosis is increased (Fig. 9A). However, the lncRNA changes in the EBV tumors in comparison to the cell line predict decreased proliferation and apoptosis, with increased migration and invasion (Fig. 9A). In contrast, comparison of the EBV tumors to pc tumors reveals that the lncRNAs and miRs both significantly activate proliferation and migration (Fig. 9). The induced lncRNAs also strongly increase invasion and viability and decrease apoptosis and necrosis. The miRs whose targets would inhibit tumor invasion, metastasis, and epithelial mesenchymal transition are downregulated or possibly sponged by the BART lncRNAs or upregulated cellular lncRNAs (Fig. 6; Fig. 9B). In the BART tumors, increased expression of BCYRN1, JPX, and TUG1 and their effects would increase migration and invasion (Fig. 9C; Table 3; Table S4C). These findings show that the effects of EBV on gene expression reflect not only changes in protein regulators but also the combination of lncRNAs and miRs.

## DISCUSSION

We previously determined that the major changes in cellular gene expression in EBV-infected gastric AGS cells grown as tumors in immunodeficient mice were due to effects on miRs that are predicted upstream regulators of the affected genes (18). In this study, the effects on the total cellular miRs and cellular lncRNAs were determined and shown to be significantly affected *in vivo* by EBV infection and by a single form of the BART lncRNAs. Comparison of all tumors to their respective cell lines revealed considerable induction of miRs that affected the expression of their known targets and predicted repression of survival, viability, proliferation, and transformation *in vivo* (Fig. 7; Fig. 9). Analysis of the induced lncRNAs in tumors compared to the corresponding cell lines also indicated repression of proliferation. In contrast, comparison of the EBV tumors to the pc tumors revealed that proliferation, migration, invasion, and viability were increased and likely reflected effects on miR activity through the induced cellular lncRNAs and the BART lncRNA.

**FIG 10:**
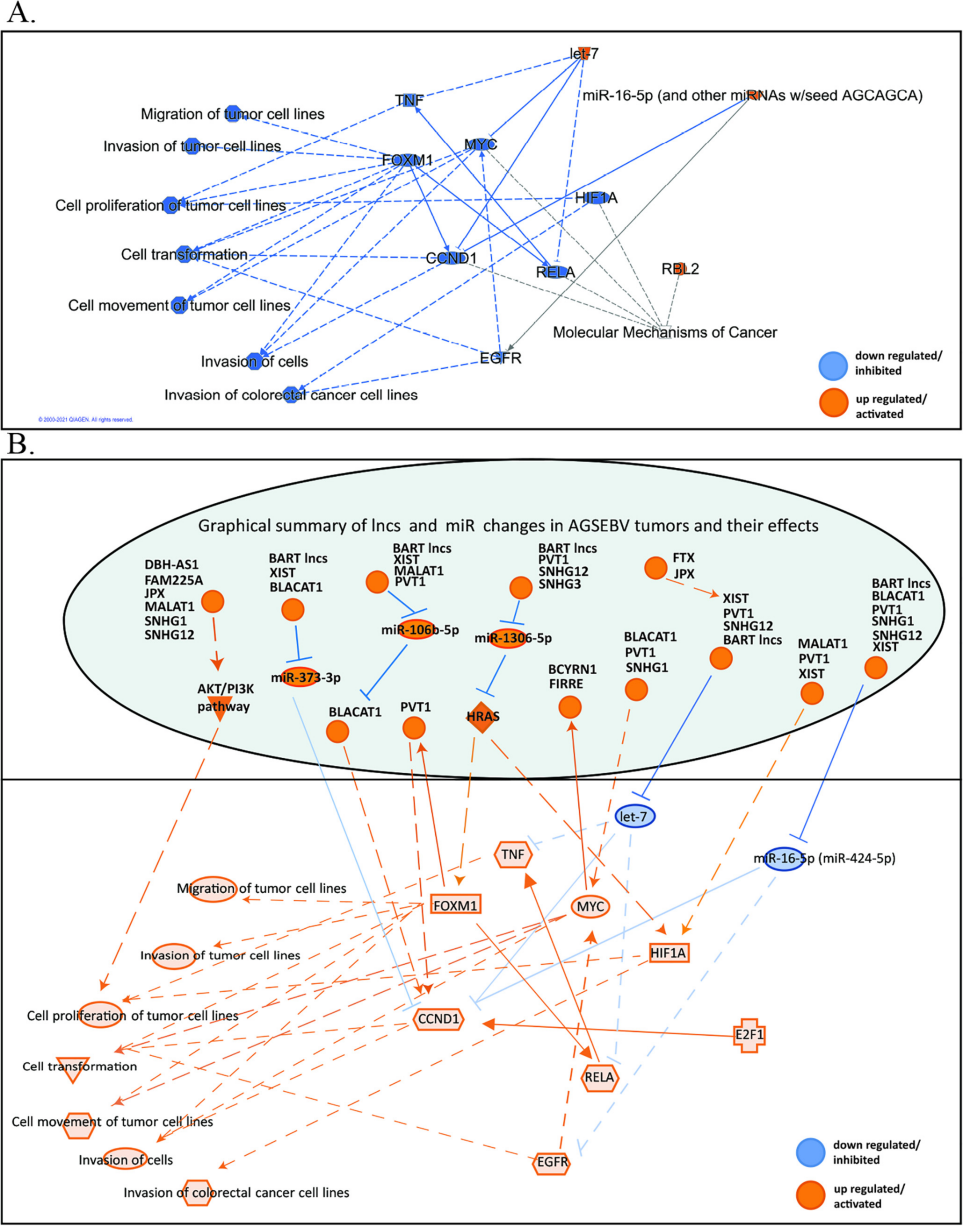
Summary of lncRNA and hsa-miR contribution to tumor development. (A) IPA-generated graphical summary of common lncRNAs and miR target changes in all the tumors compared to their cell lines with *P* value and FDR (*P* < 0.05). Blue denotes downregulation and orange upregulation. Dotted lines represent indirect relationship and solid lines represent direct relationship. (B) Unique lncRNA and hsa-miR changes in the AGS-EBV (a subset also detected in the AGS-BART) tumors compared to the AGSpc tumors that would lead to a more tumorigenic environment. Blue denotes downregulation, and orange denotes upregulation. Solid lines represent direct relationships of activation or inhibition, and dotted lines represent indirect relationships of activation or inhibition.

IPA analysis to identify common key pathways and critical points of control regulated by common lncRNAs and miRs and their target changes in the tumors compared to their cell lines revealed that the upregulation of the let7 miR family in the tumors is pivotal for the decreased activation of cell proliferation, transformation, movement, and invasion (Fig. 10A; Table S2). Let7 directly decreases c-*myc* and the cyclin-dependent kinase CCND1. The decrease in *myc* would indirectly impair all of these growth pathways. In contrast, in the EBV and BART tumors, the BART lncRNA and multiple induced cellular lncRNAs sponge let7, the miR 16-5p (424-5p), and other inhibitory miRs, resulting in the activation of Myc, tumor necrosis factor (TNF), RelA, HIF1A, FOXM1, CCND1, and E2F1 and all of the cellular functions that they regulate (Fig. 10B). The BART lncRNA potentially functions at several critical points and would inhibit miR 373-5p, which targets CCND1, miR 106b-5p, which targets BLACAT1, and miR 1306-5p, which targets RAS, in addition to having critical effects on let-7 and miR 16-5p. There is clear redundancy, with several cellular lncRNAs and the BART lncRNA targeting the same miRs (Fig. 6; Fig. 10B).

These system analyses of all potential changes in RNA populations reveal the extensive cross talk between systems and the clear interrelation between lncRNAs and miRs. Of the 698 cellular genes changed in common in the AGS-EBV and BART tumors versus pc tumors, 222 (32%) are targets of proposed sponged miRs and as such are not downregulated. The AGS cell line, like gastric cancer, is heavily methylated such that regulation through histone modification as identified in our data set in addition to lncRNA and miR modulation of expression may be particularly important (73). This is apparent in the changes induced by EBV and BART expression in the *in vivo* tumors, where many growth properties that are impaired in the control tumors are derepressed through lncRNA and miR function.

These findings reveal that a major potential mechanism for BART lncRNA function is mediated through sponging of cellular miRs. Gene expression analysis revealed that many genes involved in histone modification are also affected by EBV infection and BART expression. Importantly, three of four genes identified in methylated histone binding by GO analysis are targets of sponged miRs and as such are not downregulated and are functional in the EBV and BART tumors. Additionally, six of nine genes associated with modification-dependent protein binding are targets of the sponged miRs. Thus, it is probable that the BART lncRNA effects on miR function extend to its effects on changes in chromatin structure, which are mediated by histone methylation and acetylation.

As more characteristics of lncRNAs are identified, it is clear that the BART RNAs have many of those characteristics (20). lncRNAs are known to be variably spliced, as is the BART RNA, and many lncRNAs have been shown to encode small peptides (10, 74). The BART RNAs also contain several open reading frames (ORFs) whose peptides have been shown to have intriguing properties (Fig. 1A) (12, 75). The BART clone would contain the RB2/A73 ORF, which has been suggested to interact with RACK1, which modulates PKC and src (76). Other differently spliced BARTs have distinct ORFs, such as RK103/RPMS1, which interacts with CBF1, the intracellular signaling partner for Notch (76), and RK-BARF0, which interacts directly with Notch, in addition to epithelin, and promotes their degradation (12). Protein products of these ORFs have not been identified, so it is possible that these ORFs are just another characteristic linking the BART to cellular lncRNA properties (12). As the power of cellular lncRNAs continues to be revealed, the properties of the BART lncRNAs will contribute to our understanding of the genesis and function of this class of RNAs. Importantly, the BART lncRNA and miRs are the major viral products in type I latency and provide a mechanism for EBV to have stealth-like properties whereby cellular growth is modulated through the expression of RNAs in the absence of antigenic proteins.

## MATERIALS AND METHODS

### Ethics statement.

All animals at the University of North Carolina are maintained in compliance with the Animal Welfare Act and the Department of Health and Human Service’s *Guide for the Care and Use of Laboratory Animals* (77). UNC’s Animal Welfare Assurance Number is A3410-01. Animal experiments were performed in accordance with a protocol (no. 17-031) approved by the Institutional Animal Care and Use Committee (IACUC) at the University of North Carolina. Mice were monitored daily following injection for signs of distress and tumor growth, and postinjection general appearance and body weights were recorded. Upon observation of approved tumor endpoint growth or animal distress, the animals were euthanized by CO_2_ inhalation followed by injection of medetomidine (Domitor)/ketamine (Ketaset) (300 mg/kg and 3 mg/kg body weight).

### Cell lines.

AGSpcDNA3 (EBV-negative gastric carcinoma) cell line (AGSpc), AGS-EBV cells infected with EBV Akata BX1, and the AGS-BART1 cell line were grown as previously described (14). Cells (1 × 10^7^) were injected subcutaneously (s.c.) into NOD *scid* gamma (NSG) mice, and mice were monitored for tumor growth and illness. Tumor and spleen tissues were harvested at the endpoint.

### RNA sequencing.

RNA was prepared from the cell lines and tumors using TRIzol reagent (Life Technologies, Inc.). Poly(A)-selected, bar-coded cDNA libraries were prepared using a TruSeq stranded mRNA kit (Illumina), and the libraries were sequenced using a HiSeq 4000 instrument (Illumina) using paired-end 75-bp sequencing by the UNC High-Throughput Sequencing Facility. Small-RNA libraries were prepared from total RNA using the Bioscientific Next Flex v3 small-RNA kit and were sequenced using a HiSeq 4000 instrument (Illumina) using single-end 50-bp sequencing by the UNC High-Throughput Sequencing Facility.

### Bioinformatics.

RNA sequencing reads were aligned to the human genome (hg38) using the splicing-aware read aligner HISAT2 on the Galaxy suite (https://usegalaxy.org) and Biojupies, a web-based program available at https://amp.pharm.mssm.edu/biojupies/. Aligned reads were mapped to human Ensembl transcripts and human miRs using the Partek Genomics Suite, which was also used to calculate differential expression levels of genes, lncRNAs, and hsa-miRs and to perform gene enrichment (GO) analysis.

To quantify viral reads, sequences were aligned to the Akata genome (GenBank accession number KC207813.1). Transcripts were mapped and assembled using TopHat and StringTie on the Galaxy suite. EBV aligned transcripts were visualized from the TopHat assemblies using the Integrative Genomics Viewer. The EBV miR targets were obtained using the VIRmRNA data set (http://crdd.osdd.net/servers/virmirna/wiki.php). The sequences complementary to the hsa-miRs in the BART lncRNA exons were identified by complementation of the seed sequence in the BART exons (14). lncRNAs within our data set were identified using the lncRNAdisease 2 database (http://www.rnanut.net/lncrnadisease/index.php/home/search) and the LNC2CANCER database (http://bio-bigdata.hrbmu.edu.cn/lnc2cancer/). The interactions of hsa-miRs and cellular lncRNAs were identified using the STABASEv3.0 database (http://starbase.sysu.edu.cn).

Enriched molecular functions and pathways for the human data were obtained by running a core analysis on the statistically significant differentially expressed genes and lncRNAs (*P* values and FDR of <0.05) using IPA software (Qiagen). To identify hsa-miRs as potential regulators of gene expression, a microRNA target filter analysis was run in IPA to link differentially expressed hsa-miRs with the differentially expressed genes. The targets of the hsa-miRs were analyzed by a core analysis in IPA to determine enriched functions and pathways resulting from hsa-miR regulation. The graphical summary represents a core analysis in Ingenuity Pathway Analysis (IPA) of the common differentially expressed lncRNAs, hsa-miRs and their targets in the tumors versus the cell lines.

### Quantitative RT-PCR.

Total RNA was prepared from the tumors and cell lines using TRIzol reagent (Invitrogen), and quantitative RT-PCR was performed for the BART lncRNA as described previously (22).

### Quantitative PCR for EBV DNA.

PCR to check for EBV status in the tumors was performed in triplicate using a Quantifast SYBR green PCR kit (Qiagen) and LMP2 primers 741R (5′-AGGGGGCCTAGGTACTCTTGGTGCA-3′) and 951L (5′-CAAGTGTCCATAGGAGCATGAG-3′). Equal aliquots of the resulting PCR products were visualized on an agarose gel.

### Data availability.

The RNA sequencing files for the transcriptome analysis of the gastric samples are available at SRA under accession no. PRJNA780511. The small-RNA sequencing files for the miR analysis of the gastric samples are available at SRA under accession no. PRJNA780558.
