# Supplementary figures and images for: Major Role for Cellular MicroRNAs, Long Noncoding RNAs (lncRNAs), and the Epstein-Barr Virus-Encoded BART lncRNA during Tumor Growth In Vivo
Source: mBio. 2022 Apr 18;13(3):e00655-22. doi: 10.1128/mbio.00655-22 (PMC9239068; doi:10.1128/mbio.00655-22)

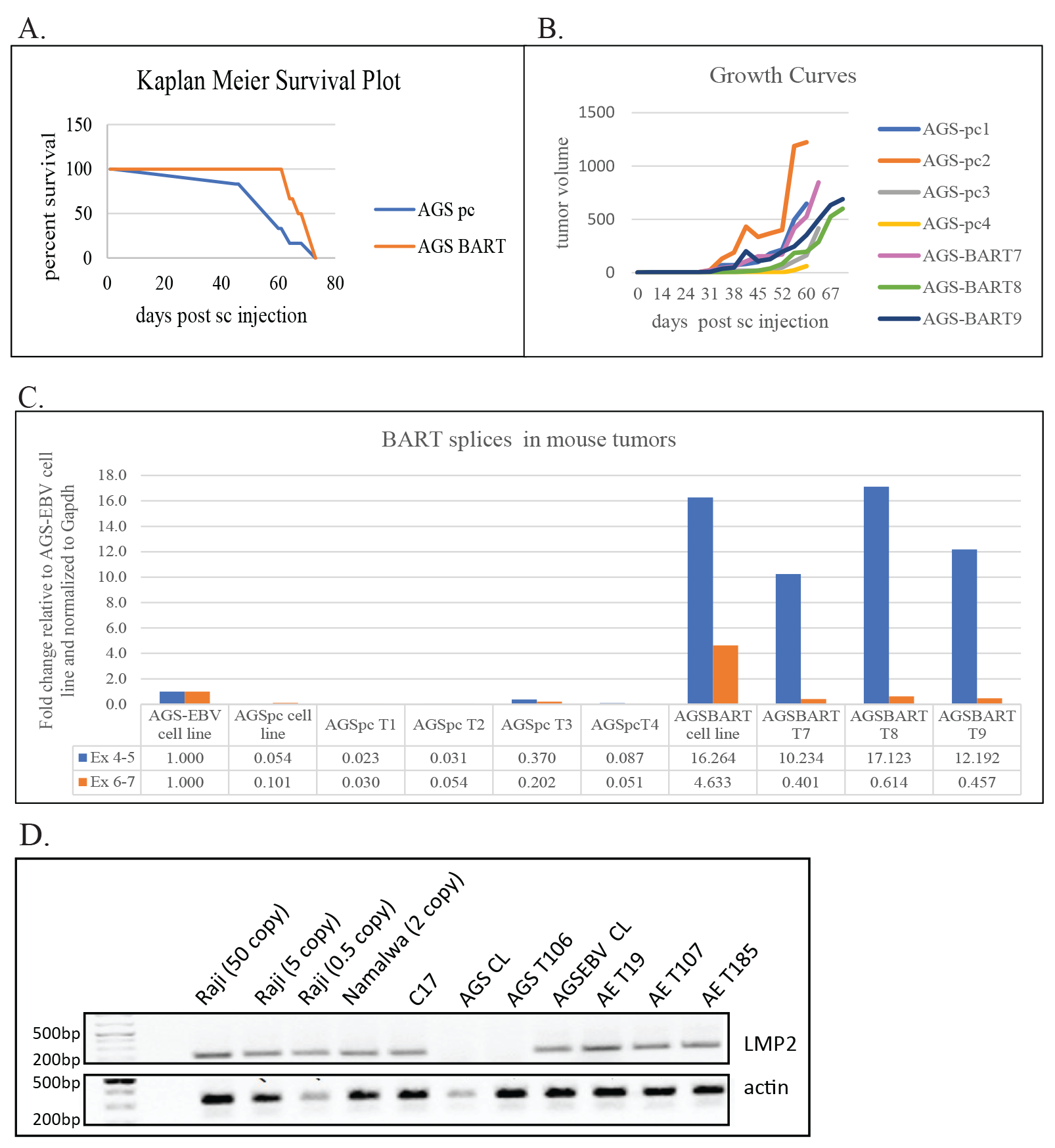

Supplement: FIG S1 [file mbio.00655-22-s0001.tif]
