# Supplementary material for: Major Role for Cellular MicroRNAs, Long Noncoding RNAs (lncRNAs), and the Epstein-Barr Virus-Encoded BART lncRNA during Tumor Growth In Vivo
Source: mBio. 2022 Apr 18;13(3):e00655-22. doi: 10.1128/mbio.00655-22 (PMC9239068; doi:10.1128/mbio.00655-22)
Supplement: TABLE S1 [file mbio.00655-22-s0002.docx]

Table S1

A. HISAT (hg38) and TopHat (Akata) summaries of reads to genomes

| Sample | Tumor and route | reads | Mapped reads to hg38 | %  to hg38 | Mapped reads to EBV (Akata) | %  to Akata |
| --- | --- | --- | --- | --- | --- | --- |
| AGSpc CL |  | 69700631 | 62145780 | 90.47 |  |  |
| AGSpc | 1(SC) | 77544718 | 64940322 | 85.29 |  |  |
| AGSpc | 2(SC) | 68793414 | 57386618 | 84.92 |  |  |
| AGSpc | 3(SC) | 67352634 | 53153376 | 80.68 |  |  |
| AGSpc | 4(SC) | 64903391 | 52064908 | 81.93 |  |  |
| AGSBART CL |  | 87888340 | 76625414 | 88.77 | 9763 | 0.01 |
| AGSBART | 7(SC) | 96792485 | 75637050 | 80.25 | 11010 | 0.01 |
| AGSBART | 8(SC) | 87315096 | 59743468 | 70.06 | 8721 | 0.01 |
| AGSBART | 9(SC) | 99335133 | 73407050 | 75.36 | 13069 | 0.01 |
| AGSEBV CL |  | 118097159 | 99920327 | 86.04 | 1749221 | 1.5 |
| AGSEBV | 107(SC) | 125046109 | 100674028 | 82.35 | 97822 | 0.1 |
| AGSEBV | 185(SC) | 138506184 | 92633327 | 69.07 | 283787 | 0.2 |

B. Small RNA reads mapped mature hsa-miRs

| Sample | Tumor and route | reads | % of reads fully overlapped with miR | % of reads partially overlapped with miR | % full and partial overlap | Total number of miRs in the model | Percentage of miRs with reads |  |
| --- | --- | --- | --- | --- | --- | --- | --- | --- |
| AGSpc CL |  | 45217804 | 40.59 | 33.30 | 73.88 | 2652 | 29.71 |  |
| AGSpc | 1(SC) | 46614261 | 35.31 | 14.54 | 49.84 | 2652 | 33.60 |  |
| AGSpc | 2(SC) | 58685128 | 34.77 | 12.73 | 47.50 | 2652 | 35.41 |  |
| AGSpc | 3(SC) | 63443430 | 42.19 | 16.26 | 58.45 | 2652 | 31.52 |  |
| AGSpc | 4(SC) | 69197992 | 24.85 | 13.08 | 37.92 | 2652 | 34.92 |  |
| AGSBART CL |  | 95937071 | 43.68 | 32.36 | 76.03 | 2652 | 26.58 |  |
| AGSBART | 7(SC) | 78294892 | 34.88 | 14.22 | 49.10 | 2652 | 34.20 |  |
| AGSBART | 8(SC) | 62357516 | 42.40 | 20.85 | 63.25 | 2652 | 38.01 |  |
| AGSBART | 9(SC) | 49437692 | 33.76 | 11.40 | 45.15 | 2652 | 32.58 |  |
| AGSEBV CL |  | 68331917 | 39.75 | 30.22 | 69.97 | 2652 | 30.09 |  |
| AGSEBV | 107(SC) | 77248126 | 31.78 | 14.26 | 46.04 | 2652 | 38.80 |  |
| AGSEBV | 185(SC) | 99192683 | 36.41 | 19.03 | 55.44 | 2652 | 37.63 |  |

C. Small RNA reads mapped to Akata

| Sample | Tumor and route | Total small reads | Total reads mapped to Akata | % total small reads to Akata |
| --- | --- | --- | --- | --- |
| AGSEBV CL |  | 68331917 | 9505347 | 12.20 |
| AGSEBV | 107(SC) | 77248126 | 7222156 | 8.55 |
| AGSEBV | 185(SC) | 99192683 | 13003908 | 11.59 |
