# Supplementary material for: Major Role for Cellular MicroRNAs, Long Noncoding RNAs (lncRNAs), and the Epstein-Barr Virus-Encoded BART lncRNA during Tumor Growth In Vivo
Source: mBio. 2022 Apr 18;13(3):e00655-22. doi: 10.1128/mbio.00655-22 (PMC9239068; doi:10.1128/mbio.00655-22)
Supplement: TABLE S3 [file mbio.00655-22-s0004.docx]

Table S3. hsa-miR upstream regulators and targets

1. AGS-EBV T vs AGS-EBV CL

| hsa-miR* | Fold change | targets changed/targets in dataset | targets |
| --- | --- | --- | --- |
| **hsa-miR-30d-5p**  miR-30c-5p (w/ seed GUAAACA)  (>0.087% total small reads mapped to hg38) | 4.26 | 57/62 | ATP2A2 (-2.0), BCL6 (-3.2), CARS1 (-3.5) CCN2 (-22.7), CEP76  (-2.4), CEP76 (-2.4), CHD1 (-2.0), CHI3L1 (-7.4), COL4A1 (-5.5), DBF4 (-2.8), DCUN1D3 (-3.9), FAM72B (-9.6), FAM72C (-11.4), FAM72D (-10.8), FBXO45( -2.1), GAN (-5.6), GTF2E2 (-1.6), HOXB8 (-1.6), IER5 (-4.7), IFRD1 (-2.0), ITGA2 (-2.0), LTN1 (-2.9), MAFA  (-2.7), MAP3K21 (-3.6), MET (-3.9), MICB (-16.3), NFAT5 (-2.9), NFATC3 (-2.2), NID1 (-2.9), NUFIP2 (-2.2), OTUD4 (-2.0), P4HA2  (-1.7), PAWR (-1.9), PLEC (-2.6), PLEKHA8 (-1.7), PNP (-3.8), POLR3G (-5.2), PPP1R18 (-6.8), PRDM1 (-19.2), PRRG1 (-7.7), RHEBL1 (-4.0), S100PBP (-2.0), SALL4 (-119.6), SEMA6B (-1.7), SH2B3 (-3.1), SLC12A4 (-3.4), SLC38A1 (-2.1), SLC4A7 (-4.0), SLC7A11 (-10.1), SNAI1 (-27.1), STX1A (-2.3), TEAD1 (-2.7), TMEM41B (-3.9), TNFRSF10B (-3.2), UAP1 (-2.4), UBE2I (-1.4), XPO1 (-1.9), YBX1 (-1.9) |
| **hsa-miR-26b-5p**  miR-26a-5p (w/ seed UCAAGUA**)**  (>0.087%) | 5.86 | 35/39 | ABL2 (-2.3), BAK1 (-2.0), C16orf70 (-2.3), CCN2 (-22.7), CDC6 (-3.2),  CDKN1A (-11.4), CEP76 (-2.4), CHAC1 (-17.7), CHD1 (-2.0), CKS2 (-3.3), DEPDC1 (-4.1), EPHA2 (-2.9), EZH2 (-1.9), GAN (-5.6), GRHL3 (-11.5), IPO7 (-3.5), JAG1 (-8.9), KIF18A (-2.4), KPNA2  (-2.5), KPNA6 (-2.1), MYC (-3.0), NAA15 (-2.0), NID1 (-2.9), NUP153 (-2.1), OTUD4 (-2.0), PDHX (-2.0), PHLDB2 (-22.9), PMAIP1 (-14.1),  POLH (-4.1), POLR3G (-5.2), RNGTT (-1.9), SACS (-4.3), SEPTIN10 (-2.2), VANGL2 (-10.6), ZNF492 (-2.6) |
| **hsa-miR-106b-5p**  miR-17-5p (w/ seed AAAGUGC)  (>0.001%) | 2.4 | 18/20 | ACTA2 (-6.7), CAMTA1 (-1.7), CCN (-4.9), CDK7 (-2.0), CDKN1A  (-11.4), CRIM1 (-3.6), EGR2 (-4.0), HBP1 (-2.0), HSF2BP (-2.7), KRT81 (-9.0), KRT86 (-8.7), MAP3K12 (-1.5), MICA (-3.1), MT2A  (-10.6), NCOA3 (-2.1), SLC1A3 (-2.0), TBC1D2 (-5.4), TXNIP (-2.8) |
| **hsa-miR-424-5p**  miR-16-5p (w/ seed AGCAGCA)  (>0.001%) | 888.5 | 37/44 | ABHD10 (-1.5), ANLN (-6.5), ARL2 (-1.5), CCNF (-2.1), CDC25A  (-1.8), CDK7 (-2.0), CENPJ (-1.7), DIPK1A (-2.1), DNAJB4 (-3.5), EGFR (-3.5), GFPT1 (-2.9), H3-3A/H3-3B (-2.0), HACE1 (-1.5), HMGA1 (-2.7), HSP90B1 (-2.6), IFRD1 (-2.0), IGF1R (-1.9), ITGA2  (-2.0), KIF23 (-2.3), KITLG (-4.5), MCL1 (-3.8), MGAT4A (-3.1), NAA15 (-2.0), PHLDB2 (-22.9), PNP (-3.8), PSAT1 (-3.7), RAD51C  (-1.70, RECK (-3.4), SEC24A (-1.9), SLC16A3 (-2.6), SLC38A1 (-2.1),  TMEM189-UBE2V1 (-3.2), TNFSF9 (-36.6), UBE2S (-4.6), UGDH  (-3.0), WEE1 (-3.7), YIF1B (-1.6) |
| **hsa-miR-590-5p**  miR-21-5p (w/ seed AGCUUAU)  (<0.001%) | 10.67 | 11/14 | FAS (-12.6), CDKN1A (-11.4), JAG1 (-9.0), ACTA2 (-6.7), SERPINB5 (-6.0), BCL2L1 (-5.2), BTG2 (-4.9), RECK (-3.4), ARF4 (-1.8), CDC25A (-1.8), MARCKS (-1.7) |
| **hsa-miR-331-3p**  (w/ seed CCCCUGG)  (>0.001%) | 3.15 | 3/3 | CDCA5 (-2.9), KIF23 (-2.3), PLAUR (-11.0) |
| hsa-miR-148b-3p  miR-148a-3p (w/ seed CAGUGCA)  (>0.087%) | 5.99 | 49/52 | ABL2 (-2.3), ACTA2 (-6.7), ADAM10 (-2.3), C16orf70 (-2.3), CD274 (-9.8), CDC25B (-4.4), CDKN1A (-11.4), CEBPG (-3.0), CHD1 (-2.0), CLOCK (-2.2), COL4A1 (-5.5), CYTH3 (-4.2), DNMT1 (-1.6), DNMT3B (-1.8), EGFR (-3.5), EPAS1 (-11.7), FAM161A (-3.0), FOXK2 (-1.4), IGF2BP3 (-32.6), ITGA5 (-1.6), KLHL5 (-7.3), KRTAP2-3 (-27.9), LIPG (-12.1), MED12L (-3.3), MOSPD1 (-2.6),  MTF1 (-1.7), MYBL1 (-2.7), NAA15 (-2.0), NPC1 (-4.8), NRP1 (-4.7),  OTUD4 (-2.0), PIDD1 (-3.8), PKIB (-5.0), PRRG1 (-7.7), PSMC3IP  (-1.5), PTPN14 (-2.5), RHOF (-7.4), RNF44 (-2.6), SESN2 (-4.0), SH2B3 (-3.1), SLC31A2 (-4.6), SMIM13 (-2.6), TAF1D (-2.8), TEAD1 (-2.7), TUBE1 (-1.5), UBA6 (-1.6), UBASH3B (-3.9), WNT10B (-2.1), ZNF488 (-25.8) |
| hsa-miR-1287-5p  (w/seed GCUGGAU)  (>0.001%) | 7.97 | 26/26 | ALPG (-59.6), APOBEC3H (-12.9), CEP57L1 (-2.2), CLOCK (-2.2), DDB2 (-6.4), DDIAS (-2.7), EGFR (-3.5), GXYLT1 (-2.4), KIAA0040 (-8.1), LIMS4 (-3.8), MAP3K21 (-3.5), MPRIP (-2.1), MUC13 (-21.00, NUP153 (-2.1), PLEKHA8 (-1.7), PRIM2 (-1.9), PRSS35 (-13.1), PSIP1 (-1.6), PSRC1 (-2.0), RGS16 (-11.0), SHC1 (-3.5), SULT1C4 (-92.7), TEAD3 (-2.3), TLR4 (-2.6), YBX1 (-1.90, YBX3 (-3.7) |
| hsa-miR-107  miR-103-3p (w/seed GCAGCAU)  (>0.001%) | 2.3 | 112/171 | AARS1 (-4.7), ABL2 (-2.30, ACVR2B (-4.1), ADAM10 (-2.3), ADGRA2 (-10.3), AGO2 (-2.2), AIP (-2.6), ARHGAP19 (-6.0), ARIH1 (-1.6), ATP13A3 (-2.2), B3GNT2 (-4.1), BAK1 (-2.0), BOLA3 (-1.4), BTG2 (-4.9), C11orf68 (-2.7), C16orf72 (-1.9), CCDC50 (-2.2), CDC25A (-1.7), CDC37L1 (-1.9), CENPM (-1.4), CEP85L (-4.7), CHAD (-3.3), CHD1 (-2.0), CISD1 (-1.7), CLOCK (-2.2), CRELD1  (-2.3), CRKL (-1.8), CRYBG2 (-8.5), DCUN1D3 (-3.9), DNAJB4  (-3.5), DUSP14 (-2.4), EDA (-7.7), FBXW7 (-2.1), FCHO1 (-5.6), FDXR (-14.1), GAS2L3 (-2.0), GCNT3 (-36.2), GLUD2 (-4.0), GNAI3 (-1.9), GRB7 (-6.2), HELZ (-1.4), HMGA1 (-2.7), HSPE1 (-1.4), IDH3A (-1.9), IGSF3 (-3.1), IL10RB (-1.6), JADE1 (-1.7), KATNBL1 (-2.2), KCND1 (-1.6), KCNK1 (-3.6), KIAA0040 (-8.1), KIF18B (-2.2), KIF23 (-2.3), KLF13 (-1.5), LCOR (-2.1), LONRF1 (-2.4), MAP3K21  (-3.5), MAP3K3 (-2.1), MBNL2 (-4.7), MIB1 (-3.1), MICB (-16.3), MTHFD1L (-2.9), MTMR4 (-2.5), MYBL1 (-2.6), MYEOV (-6.5), MYNN (-1.7), NAA15 (-2.0), OPRL1 (-1.9), ORC6 (-1.7), OTUD4  (-2.0), PAWR (-1.9), PDE8B (-9.3), PLA2G2F (-8.8), PMM1 (-5.4), POU2F1 (-1.7), PRKAB2 (-2.9), PRRG1 (-7.7), RCAN1 (-2.6), RFWD3 (-1.6), RGPD4 (-3.0), RNF24 (-2.8), SALL4 (-119.6), SAV1 (-3.4), SEPTIN5 (-4.4), SGO1 (-2.3), SIPA1L2 (-111.4), SLC48A1 (-2.0), SNAP47 (-2.4), SNCG (-5.9), SOCS2 (-2.3), SOWAHC (-10.0), STAMBPL1 (-2.1), SYDE2 (-3.1), SYNJ1 (-2.0), TAF5 (-3.1), TENT4B (-1.8), TFRC (-7.4), TGFBR3 (-2.4), TMEM143 (-1.7), TNIK (-3.3), TNPO1 (-2.5), TRANK1 (-3.1), TRIAP1 (-1.8), TRIM59 (-1.6), TRMT112 (-1.4), TUBGCP3 (-2.1), VCL (-3.5), VDAC2 (3.1), XKR8  (-1.6), ZBTB10 (-2.4), ZBTB34 (-2.1), ZNF273 (-2.6), ZNF526 (-1.4) |
| hsa-miR-503-5p  (w/ seed AGCAGCG)  (>0.001%) | 198.4 | 5/7 | ANLN (-6.5), CCNF (-2.1), CDC25A (-1.8), CDKN1A (-11.4), WEE1  (-3.7) |
| hsa-miR-3074-5p  (w/seed UUCCUGC)  (>0.001%) | 7.1 | 8/8 | IGFL2 (-15.0), PMAIP1 (-14.1), RHOV (-9.5), EML2 (-5.2), NEK2  (-3.5), DDIAS (-2.7), PIMREG (-2.2), SLC25A19 (-1.7) |
| hsa-let-7f-1-3p  let-7a-3p (w/seed UAUACAA)  (>0.001%) | -1.6 | 23/99 | ANKHD1 (2.8), ANKHD1-EIF4EBP3 (2.8), C1orf216 (2.5), COX19 (2.0), CPNE2 (7.5), EIF4ENIF1 (2.0), FAM160A2 (1.7), FZD6 (2.8), GPCPD1 (3.0), INO80D (2.2), KIN (2.2), LLPH (2.5), LYSMD4 (1.4), MKRN3 (2.0), MOK (9.5), MTARC1 (3.0), PELI1 (3.0), PLPP1 (2.6), PPP1R3F (5.6), PTBP2 (2.5), SCX (4.7), TBC1D2B (2.0), TSPAN13 (2.1) |
| hsa-miR-125a-3p  (w/seed CAGGUGA)  (>0.001%) | -2.2 | 53/117 | ACP2 (3.4), AGPAT3 (2.0), ARL1 (2.2), ATG10 (1.8), BIN3 (5.3), BSCL2 (4.5), C12orf65 (3.3), C2orf81 (2.2), CRCP (2.0), DAP (3.3), DNAJC22 (24.0), DPM2 (1.7), DPYSL3 (7.5), FAIM2 (678.5), FAM227A (7.4), FANCC (1.3), GPN2 (1.7), GPR157 (2.4), HGH1 (5.9), HMGA2-AS1 (15.5), IFT22 (2.9), IQCC (3.1), ITPKB (2.9), JMJD8 (2.9), KIAA0319L (3.1), KIF13B (3.1), KLK6 (13.8), LGALS3BP (22.6), MFSD14C (2.6), MPI (1.6), MSANTD2 (2.9), MTERF4 (2.4), MTRES1 (3.6), NBPF10 (1.8), NCDN (5.0), PRKAG1 (2.7), PRKAR1B (5.0), PRPF38A (1.4), RMND5B (1.9), RNF185 (2.4), ROBO3 (1.6), RUNDC1 (2.0), SCN8A (14.4), SERPIND1 (21.6), SLC22A23 (2.3), TEX261 (4.9), TIRAP (3.3), TMBIM4 (3.5), TMEM150A (4.7), TRMT10B (1.9), VPS52 (1.6), ZNF510 (2.1), ZNF785 (2.9) |
| hsa-miR-1292-5p miR-1247-3p  (w/seed GGGAACG)  (>0.001%) | -2.2 | 25/70 | C1orf216 (2.5), CCDC127 (1.8), CLDN2 (703.0), CLPTM1L (1.9), DNAJC4 (1.9), GATD1 (2.6), LUC7L (2.2), MANEAL (1.7), MAP11 (2.3), MARCHF9 (1.6), MARCKSL1 (2.0), MED22 (2.2), MGST2 (3.0), MRPL20-AS1 (2.9), MSRB2 (3.4), PILRB (1.9), PLA2G6 (1.6), SNAPIN (2.2), SRSF6 (1.7), SWI5 (2.8), TK2 (2.8), UNC119B (2.4), ZNF23 (3.5), ZNF436-AS1 (2.3), ZNF707 (2.3) |
| hsa-miR-21-3p  miR-122b-3p (w/seed AACACCA)  (>0.001%) | -3.9 | 23/90 | ATG10 (1.8), CDK2AP1 (3.4), DDX31 (1.5), DHRSX (3.6), DUSP22 (2.3), FOXD4 (11.6), GPCPD1 (3.0), HOXC9 (5.6), IL17RD (4.0), KLHL3 (3.4), LYSMD2 (3.8), MEIS2 (5.8), MSS51 (3.6), OARD1 (3.6), PLPP5 (2.0), RABL2A (2.1), RDH14 (4.5), SIK2 (2.5), WDCP (4.4), ZBTB18 (1.8), ZCCHC24 (3.9), ZNF302 (5.2), ZNF493 (3.8) |
| hsa-miR-30b-3p  miR-1273h-5p (w/seed UGGGAGG)  (>0.001%) | -4.4 | 80/172 | ACP2 (3.4), ALDH16A1 (1.5), ALG1 (4.3), ANKRD52 (2.3), ANKRD54 (1.7), ARF5 (1.9), ASB16 (3.6), ATP7B (3.4), BGLAP (3.8), BICDL1 (1.7), C9orf85 (2.4), CACNB3 (2.1), CCDC24 (2.6), CCZ1/CCZ1B (2.1), CDK10 (1.4), CDK2AP1 (3.4), CEMP1 (18.7), CENPS (3.7), CENPS-CORT (3.7), CLDN2 (703.0), CPLX2 (17.8), CPSF1 (1.6), CPTP (2.0), CRTC1 (3.8), CTDSP1 (14.7), CYB5D1 (4.2), DAP (3.3), DBNL (2.4), DFFB (1.9), DMTN (2.9), DNAH10OS (5.1), DNAJB2 (1.6), DNAJC14 (2.3), DPM2 (1.7), DRG2 (2.1), EFNA4 (2.1), ENTPD2 (6.4), ERBB3 (6.9), FAIM2 (678.5), FAM219B (1.7), FBXO10 (2.2), FGFR4 (6.6), FIZ1 (2.1), FKBP11 (3.1), FLAD1 (2.1), FOXP4 (2.1), FURIN (3.1), GATD1 (2.6), GOSR1 (2.0), GPN2 (1.7), GPR157 (2.4), GPX2 (6.9), GSTM1 (4.0), H6PD (3.2), HDAC10 (1.6), HFE (4.7), HGH1 (5.9), HMGA2-AS1 (15.5), IDUA (3.6), IFT22 (2.9), IHH (558.6), IL17RD (4.0), IRAK4 (3.6), KLHDC4 (2.4), LBHD1 (18.5), LCMT2 (8.3), LFNG (54.5), LHX4 (12.7), MARCHF9 (1.6), MARCKSL1 (2.0), MBD3 (2.4), MBD6 (2.7), METTL1 (3.6), MFSD14C (2.6),MINDY1 (3.9), MLLT6 (3.3), MOB3A (2.5), MRM2 (2.3), MSS51 (3.6), MTHFSD (2.0) |
| hsa-miR-942-5p  miR-12202-3p (w/seed CUUCUCU)  (>0.001%) | -6.3 | 27/73 | ARL17A/ARL17B (1.8), C12orf43 (2.7), C12orf49 (2.0), DTX1 (10.0), GNPTG (2.0), HIPK2 (4.8), HNRNPU (1.8), HS3ST1 (3.2), ITGA10 (6.6), KRBOX4 (1.8), LBHD1 (18.5), MESD (3.3), MTARC1 (3.0), NBPF10 (1.8), NTM (15.7), RNFT2 (1.3), RSAD1 (1.9), SWI5 (2.8), TMEM19 (4.7), UBE3D (1.9), ZBTB18 (1.8), ZBTB26 (2.6), ZNF23 (3.5), ZNF302 (5.2), ZNF37A (2.0), ZNF544 (2.4), ZNF75D (5.7) |

*bolded miRs are predicted to be activated or inhibited as upstream regulators (z score ≥ 2 activated) or (z score ≤ -2

inhibited)

All miRs listed have expression changes with significant p values (<0.05).

(% total small reads mapped to hg38) The average %=0.087%

1. BART T vs BART CL

| hsa-miR* | Fold change | targets changed/targets in dataset | targets |
| --- | --- | --- | --- |
| **hsa-let-7a-5p**  (w/seed GAGGUAG)  (>0.087%) | 3.3 | 149/176 | ABCB9 (-4.0), ABL2 (-1.9), ACVR2B (-2.9), AEN (-3.2), AHCTF1 (-1.8), AKNA (-4.3), ANAPC1 (-2.3), ARG2 (-4.2), ARID3A (-7.7), ARID3B (-1.7), ARRDC4 (-3.0), ASAP1 (-4.7), ATAD3B (-3.3), AURKB (-2.9), BMP2K (-2.4), BTF3L4 (-1.5), BZW2 (-1.8), C6orf120 (-1.9), CALCOCO2 (-1.7), CBL (-2.3), CCDC71L (-2.1), CCND1 (-2.0),  CCNF (-2.9), CDC25A (-2.5), CDC25B (-3.7), CDC34 (-2.0), CDCA8 (-1.8), CDK6 (-2.1), CDK8 (-1.7), CDKN1A (-8.1), CENPK (-2.0), CEP120 (-2.1), CHD7 (-2.0), CLASP2 (-1.5), CLOCK (-1.8), COL4A1 (-22.4), COL4A6 (-2.5), CPSF4 (-2.3), CTPS1 (-2.9), DCBLD1 (-2.4), DLC1 (-4.9), DLST (-1.6), DOCK5 (-2.6), EDN1 (-2.8), ESPL1 (-2.5), FAM135A (-2.4), FAM72A (-4.7), FAM72B (-4.4), FAM72C (-4.9), FANCD2 (-2.4), FAS (-3.9), FBXO30 (-2.6), FRMD4B (-2.2), GAN  (-2.4), GPAT3 (-16.7), GRPEL2 (-3.1), GTPBP3 (-2.1), GXYLT1 (-1.8), HASPIN (-2.5), HIC2 (-3.0), HMGA1 (-2.5), IFRD1 (-3.9), IGF1R  (-1.7), IGF2BP2 (-1.8), IGF2BP3 (-86.9), IQCB1 (-1.9), KIF2A (-1.9), KLF9 (-2.3), LBH (-13.4), LBR (-2.4), MAPK11 (-2.4), MEIS3 (-18.1), MGME1 (-1.8), MIB1 (-1.8), MIEF1 (-1.9), MIOS (-1.6), MLLT10  (-1.7), MSN (-2.5), MTRR (-2.3), MYC (-2.7), MYCL (-3.1), NARS2  (-1.8), NEDD4 (-1.8), NEMP1 (-2.0), NF2 (-2.0), NID1 (-33.8), NUDT15 (-2.0), NUMBL (-4.9), NUP155 (-2.7), NXN (-3.4), OLR1  (-1416.7), OSBPL3 (-2.7), PKN3 (-2.1), PLCXD1 (-2.1), PLEKHA8  (-1.6), PLSCR3 (-3.0), PM20D2 (-2.4), PMAIP1 (-19.8), POC1A (-1.8), POLQ (-2.2), POLR2D (-1.6), POLR3D (-1.6), PPARGC1B (-1.9), PPAT (-2.4), PRDM1 (-10.0), PRIM1 (-3.0), PRIM2 (-2.1), PRKAB2  (-2.3), PRRC2A (-1.4), PTGS2 (-10.2), RGPD8 (-2.3), RGS16 (-7.4), RNF44 (-2.2), RPP38 (-1.5), RPUSD2 (-1.5), RPUSD3 (-2.1), RRP1B  (-2.0), RSKR (-2.3), RTCA (-1.8), S100PBP (-1.9), SALL4 (-45.0), SH2B3 (-7.5), SLC1A4 (-3.4), SLC31A2 (-5.2), SLC38A1 (-3.5), SLC5A6 (-2.0), SMC1A (-1.6), SMOX (-2.3), SNAI3 (-3.0), SNX30  (-1.7), STARD4 (-2.0), SYNCRIP (-1.7), TAGLN (-2.8), TEAD3 (-2.2), TK1 (-2.7), TLR4 (-2.2), TNIK (-1.9), TRMT1 (-1.7), TTC9C (-1.4), TTL (-2.1), TTLL (-1.9), UHRF1 (-2.2), USP38 (-2.0), VANGL2  (-22.5), VSNL1 (-3.0), VSTM5 (-5.6), XKR8 (-1.9), ZC3H3 (-1.7), ZNF697 (-2.0) |
| **hsa-miR-30d-5p**  (w/ seed GUAAACA)  (>0.087%) | 3.3 | 49/54 | CARS1 (-4.2), CCN2 (-5.0), CDCP1 (-3.1), CEP76 (-2.3), CHD1 (-1.9),  CHI3L1 (-386.5), COL4A1 (-22.4), DBF4 (-3.0), DCUN1D3 (-3.3), FAM72B (-4.3), FAM72C (-4.9), FAM72D (-3.9), FBXO45 (-1.7), GAN (-2.4), GTF2E2 (-1.5), HOXB8 (-8.7), IER5 (-3.3), IFRD1 (-3.9),  ITGA2 (-1.7), LMNB2 (-1.5), MAP3K21 (-3.1), MICB (-3.5), NCL  (-1.7), NFAT5 (-1.7), NFATC3 (-2.1), NID1 (-33.8), OTUD4 (-1.8), PAWR (-3.1), PLEKHA8 (-1.6), POLR3G (-27.4), PRDM1 (-10.0), PRRG1 (-4.6), PTRH1 (-1.6), RAD23B (-1.5), RHEBL1 (-6.3), S100PBP (-1.9), SALL4 (-45.0), SEMA6B (-6.8), SH2B3 (-7.5), SLC38A1 (-3.5), SLC38A2 (-2.0), SLC4A7 (-3.3), SLC7A1 (-3.8), SLC7A11 (-11.7), SNAI1 (-16.5), TEAD1 (-2.3), UAP1 (-1.7), XPO1  (-2.0), YBX1 (-1.8) |
| **hsa-miR-26a-5p**  (w/ seed UCAAGUA)  (>0.087%) | 2.9 | 36/39 | ABL2 (-1.9), BAK1 (-1.5), C16orf70 (-2.0), CCN2 (-5.0), CDK6 (-2.1), CDKN1A (-8.1), CEP76 (-2.3), CHAC1 (-102.1), CHD1 (-1.8), CKS2 (-2.2), DEPDC1 (-2.6), EZH2 (-3.8), GAN (-2.4), GRHL3 (-2.1), HPGD (-34.3), IPO7 (-1.7), JAG1 (-2.5), KIF18A (-2.3), KPNA2 (-1.6), KPNA6 (-1.7), MYC (-2.7), NAA15 (-2.2), NID1 (-33.8), NUP153  (-1.6), OTUD4 (-1.8), PDHX (-1.7), PHLDB2 (-47.2), PMAIP1 (-19.8), POLH (-1.7), POLR3G (-27.4), PTGS2 (-10.2), RNGTT (-1.8), SACS  (-3.1), SEPTIN10 (-3.3), VANGL2 (-22.5), ZNF492 (-2.6) |
| **hsa-miR-375-3p**  (w/ seed UUGUUCG)  (>0.087%) | 104.3 | 4/4 | C1QBP (-2.3), CIP2A (-1.7), MYC (-2.7), YAP1 (-1.8) |
| **hsa-miR-424-5p**  miR-16-5p (w/ seed AGCAGCA)  (>0.001%) | Turned on | 36/41 | ABCF2 (-1.7), CCND1 (-2.0), CCNF (-2.9), CDC25A (-2.5), CDK6  (-2.1), CDK7 (-1.8), CENPJ (-1.8), CHEK1 (-2.3), DIPK1A (-1.9), EGFR (-2.5), GRB10 (-3.3), HACE1 (-1.7), HMGA1 (-2.5), IFRD1  (-3.9), IGFR1 (-1.7), ITGA2 (-1.7), KIF23 (-2.4), KPNA3 (-1.9), LUZP1 (-1.6), MGAT4A (-2.4), MSH2 (-1.8), NAA15 (-2.2), NOTCH2 (-1.9), PHLDB2 (-47.2), PLK1 (-2.8), PRIM1 (-3.0), PSAT1 (-8.9), PTGS2 (-10.2), RAD51C (-1.9), RECK (-2.4), RFT1 (-1.6), SLC25A22 (-1.6), SLC38A1 (-3.5), SLC7A1 (-3.8), UBE2S (-2.8), UTP15 (-2.0) |
| hsa-miR-148a-3p  (w/ seed CAGUGCA)  (>0.087%) | 11.0 | 49/52 | ABL2 (-1.9), ACTA2 (-5.5), ADAM10 (-1.7), C16orf70 (-2.0), CD274 (-3.1), CDC25B (-3.7), CDKN1A (-8.1), CEBPG (-2.6), CHD1 (-1.8), CLOCK (-1.8), COL4A1 (-22.4), CYTH3 (-9.2), DNMT1 (-2.0), DNMT3B (-3.5), EGFR (-2.5), EPAS1 (-4.2), FAM161A (-1.7), FOXK2 (-1.3), IGF2BP3 (-86.9), ITGA5 (-3.6), KLHL5 (-10.2), KRTAP2-3  (-145.0), LIPG (-4.4), MED12L (-3.4), MOSPD1 (-2.2), MTF1 (-1.4), MYBL1 (-2.4), NAA15 (-2.2), NPC1 (-2.3), NRP1 (-4.4), OTUD4  (-1.8), PIDD1 (-3.5), PKIB (-11.3), PRRG1 (-4.6), PSMC3IP (-2.7), PTPN14 (-2.3), RHOF (-2.9), RNF44 (-2.2), SESN2 (-4.7), SH2B3  (-7.5), SLC31A2 (-5.2), SMIM13 (-2.2), TAF1D (-2.1), TEAD1 (-2.3), TUBE1 (-4.4), UBA6 (-1.6), UBASH3B (-2.9), WNT10B (-4.6), ZNF488 (-8.0) |
| hsa-miR-1287-5p  (w/seed GCUGGAU)  (>0.001%) | 530.5 | 26/26 | ALPG (-1192.7), APOBEC3H (-16.3), CEP57L1 (-2.3), CLOCK (-1.8),  DDB2 (-3.3), DDIAS (-2.3), EGFR (-2.5), GXYLT1 (-1.8), KIAA0040 (-3.4), LIMS4 (-3.2), MAP3K21 (-3.1), MPRIP (-2.2), MUC13 (-2.1), NUP153 (-1.6), PLEKHA8 (-1.6), PRIM2 (-2.1), PRSS35 (-44.7), PSIP1 (-2.4), PSRC1 (-1.5), RGS16 (-7.4), SHC1 (-2.7), SULT1C4 (-1093.1), TEAD3 (-2.1), TLR4 (-2.2), YBX1 (-1.8), YBX3 (-5.3) |
| hsa-miR-3074-5p  (w/seed UUCCUGC)  (>0.001%) | 2.3 | 8/8 | IGFL2 (-22.3), PMAIP1 (-19.8), RHOV (-4.3), DDIAS (-2.3), SLC25A19 (-2.1), NEK2 (-2.0), EML2 (-2.0), PIMREG (-1.9) |
| hsa-let-7f-1-3p  let-7a-3p (w/seed UAUACAA)  (>0.001%) | -2.6 | 16/81 | AHR (2.5), ARID4B (1.7), BTG1 (3.6), CASC4 (2.0), CCDC186 (2.1), DCAF6 (2.3), FLT3LG (2.0), HSD17B11 (4.8), ITM2B (2.8), KCNE3 (8.6), KMT2E (2.3), MOK (3.8), PLPP1 (1.7), PLPP3 (5.5), PPM1L (22.6), TMEM263 (3.1) |
| hsa-miR-105-5p  (w/seed CAAAUGC)  (>0.001%) | -203.2 | 15/44 | CDK2AP1 (2.6), COA3 (1.6), CRYBG3 (1.8), CRYL1 (7.3), CYP3A5 (5.1), H3-3A/H3-3B (1.9), IKZF2 (3.6), ITM2B (2.8), NDUFA2 (1.5), PARP3 (4.9), RNASE4 (43.1), SEC62 (2.1), SELL (4.8), SH3YL1 (4.2), SLC5A1 (18.6) |
| hsa-miR-21-3p  miR-122b-3p (w/seed AACACCA)  (>0.001%) | -3.5 | 15/62 | ATF2 (2.0), BTG1 (3.6), CBLB (1.8), CDC42EP4 (2.5), CDK2AP1 (2.6), DDAH1 (2.0), DNAH6 (27.0), DRAM1 (2.4), H3-3A (1.9), MAT2B (1.9), MEIS2 (4.8), PLEKHS1 (516.1), POLD3 (3.0), SKAP2 (2.1), ZBTB18 (2.7) |
| hsa-miR-942-5p  miR-12202-3p (w/seed CUUCUCU)  (>0.001%) | -3.7 | 13/36 | ATF2 (2.0), CBLB (1.8), CYP3A5 (5.1), DRAM1 (2.4), GNPTG (1.9), HIPK2 (3.6), KCNE3 (8.6), MAT2B (1.9), PCDHB12 (27.4), POLD3 (3.0), TMEM263 (3.1), TOGARAM1 (1.6), ZBTB18 (2.7) |

*bolded miRs are predicted to be activated or inhibited as upstream regulators (z score ≥ 2 activated) or (z score ≤ -2

inhibited)

All miRs listed have expression changes with significant p values (<0.05).

(% total small reads mapped to hg38) The average %=0.087%

1. pc T vs pc CL

| hsa-miR* | Fold change | targets changed/targets in dataset | targets |
| --- | --- | --- | --- |
| **hsa-let-7b-5p**  let-7a-5p (w/seed GAGGUAG)  (>0.087%) | 3.2 | 223/235 | ABCB9 (-3.9), ABCC10 (-1.7), ABCC5 (-1.6), ABL2 (-2.0), ACER2  (-2.0), ACTA2 (-5.1), ACVR2B (-2.7), AEN (-3.7), AHCTF1 (-2.1), ANAPC1 (-2.4), AP1S1 (-1.8), ARG2 (-3.8), ARHGEF39 (-2.1), ARID3A (-7.7), ARID3B (-1.9), ARL5A (-1.8), ARRDC4 (-2.7), ASAP1 (-3.3), ATAD3B (-4.2), ATG10 (-1.9), ATP13A3 (-2.0), AURKB (-4.5), BDP1 (-1.6), BIRC5 (-2.2), BMP2K (-2.8), BRCA2  (-2.2), BTG2 (-2.2), BZW2 (-2.4), C19orf53 (-1.4), CALM1 (-1.9), CAP1 (-2.2), CARHSP1 (-2.1), CBL (-1.8), CCND1 (-2.9), CCNE1  (-3.1), CCNF (-3.9), CDC25A (-3.1), CDC25B (-3.4), CDC34 (-2.2), CDC42SE1 (-1.6), CDCA8 (-2.5), CDK6 (-3.0), CDK7 (-1.9), CDKN1A (-6.7), CENPK (-2.7), CEP120 (-1.8), CEP135 (-2.4), CEP164 (-1.7),  CHCHD1 (-1.5), CHD7 (-1.9), CIAO2A (-2.0), CLASP2 (-1.7), CLOCK (-2.0), CLP1 (-1.6), COL4A1 (-13.6), COL4A6 (-5.1), CPA4 (-13.3), CPSF4 (-2.0), CTPS1 (-3.4), DCBLD1 (-2.2), DDX19A (-1.6), DLC1  (-4.8), DLST (-1.9), DOCK5 (-2.8), DPH3 (-1.9), DPP3 (-1.8), DPYSL (-7.3), DUSP4 (-2.9), EDN1 (-4.6), EIF3J (-1.6), ELP1 (-2.4), ESPL1  (-2.4), ESRP2 (-1.9), FAM135A (-4.0), FAM189B (-1.5), FAM72A (-4.7), FAM72B (-5.5), FAM72C/FAM72D (-6.2), FANCD2 (-3.0), FAS (-5.8), FBXO30 (-3.7), FRMD4B (-2.5), FUT4 (-3.9), GALNT2 (-1.9), GAN (-2.9), GCNT4 (-12.9), GEMIN7 (-1.7), GNG5 (-2.1), GPAT3  (-7.5), GPX3 (-2.1), GRPEL2 (-2.0), GTPBP3 (-2.3), GXYLT1 (-2.1), GYS1 (-1.5), HASPIN (-2.8), HIC2 (-3.5), HSPE1-MOB4 (-2.1), ICMT (-1.8), IFRD1 (-2.4), IGF1R (-1.6), IGF2BP2 (-1.5), IGF2BP3 (-18.9), IL22RA1 (-3.1), IPO4 (-2.3), IQCB1 (-1.9), ISCA2 (-2.0), ITSN1 (-3.3), KIF2A (-2.2), LBH (-5.2), LBR (-2.5), LGR4 (-1.8), LIMD1 (-1.5), LOXL3 (-2.6), LRRC20 (-2.1), LSM11 (-1.6), MAGEA2 (-23.5), MAP3K3 (-1.8), MAPK11 (-4.0), MEIS3 (-74.6), MGME1 (-2.6), MIB1 (-1.5), MIEF1 (-1.9), MIOS (-2.2), MLLT1 (-1.4), MPHOSPH6 (-2.3),  MSN (-2.8), MT-ND4L (-1.8), MTRR (-2.4), MYC (-3.9), MYCBP (-1.5), NAA20 (-2.1), NARS2 (-2.1), NEMP1 (-2.8), NF2 (-2.1), NID1 (-29.5), NUDT15 (-1.9), NUMBL (-4.0), NUP155 (-3.0), NXN (-6.1), OLR1 (-133.4), ONECUT2 (-7.7), OSBPL3 (-3.8), OTULINL (-3.4), PARD6B (-2.6), PDP2 (-1.9), PDSS1 (-2.7), PKN3 (-2.0), PLCXD1 (-3.1), PLEKHA8 (-2.2), PLSCR3 (-4.2), PM20D2 (-4.6), PMAIP1 (-5.8), POC1A (-2.4), POLQ (-3.7), POLR2D (-2.1), POLR3D (-1.9), PPARGC1B (-2.7), PPAT (-2.6), PPP2R2A (-1.7), PRDM1 (-4.4), PRIM1 (-2.6), PRIM2 (-2.1), PRKAB2 (-1.8), PRRC2A (-1.5), PTGS (-11.3), RBM19 (-1.6), RDH10 (-2.0), RGPD4 (-1.9), RGS16 (-11.8), RNF44 (-2.0), RPP38 (-1.7), RPUSD2 (-1.7), RPUSD3 (-2.6), RRP1B (-2.4), RSKR (-3.2), RTCA (-1.8), RTKN (-2.3), S100PBP (-2.0), SALL4 (-17.5), SBK1 (-3.5), SENP5 (-1.6), SH2B3 (-2.8), SIGMAR1 (-1.7), SLC1A4 (-2.2), SLC20A1 (-1.4), SLC25A13 (-1.6), SLC25A32 (-1.8), SLC31A2 (-9.1), SLC38A1 (-2.1), SLC5A6 (-2.4), SMC1A (-2.2), SNAI3 (-3.5), SNN (-2.3), SOCS4 (-1.8), SPINDOC (-2.0), STARD4 (-3.7), STRBP (-1.6), TAF5 (-2.3), TAGLN (-7.1), TEAD3 (-2.0), TK1 (-3.8), TLR4 (-4.9), TNFAIP8L1 (-1.8), TNIK (-2.1), TRIB1 (-2.4), TRMT1 (-2.1), TTC9C (-1.5), TTL (-2.9), TTLL4 (-2.1), TUSC2 (-1.5), TYMS (-2.6), UBE2G2 (-2.5), UHRF1 (-2.6), USP38 (-1.9), VANGL2 (-13.2), XKR8 (-2.1), ZC3H3 (-1.7), ZCCHC9 (-1.6), ZNF341 (-1.5), ZNF343 (-1.8), ZNF473 (-1.6), ZNF697 (-1.5), ZNF765 (-1.5) |
| **hsa-miR-26b-5p**  miR-26a-5p (w/ seed UCAAGUA)  (>0.087%) | 5.8 | 39/43 | ABL2 (-2.0), BAK1 (-2.0), C16orf70 (-2.7), CCN2 (-12.7), CCNE1 (-3.1), CCNE2 (-2.4), CDK6 (-3.0), CDKN1A (-6.7), CEP76 (-2.9), CHAC1 (-19.1), CHD1 (-2.1), CHORDC1 (-3.5), CKS2 (-4.5), DEPDC1 (-3.5), EZH2 (-4.8), GAN (-2.9), GRHL3 (-2.4), HPGD (-14.2), IPO7 (-2.3), JAG1 (-3.4), KIF18A (-2.1), KPNA2 (-2.8), KPNA6 (-1.9), MYC (-3.9), NAA15 (-2.8), NID1 (-29.5), NUP153 (-1.8), OTUD4 (-1.8), PDHX (-1.8), PHLDB2 (-30.8), PMAIP1 (-5.8), POLH (-3.3), POLR3G (-17.7), PTGS2 (-11.3), RNGTT (-1.7), SACS (-3.6), SEPTIN10 (-2.6), VANGL2 (-13.2), ZNF492 (-3.0) |
| **hsa-miR-424-5p**  miR-16-5p (w/ seed AGCAGCA)  (>0.001%) | Turned on | 48/50 | ABCF2 (-1.9), ANLN (-3.2), ARL2 (-1.9), ASXL2 (-1.5), CCND1 (-2.9), CCNE1 (-3.0), CCNF (-3.9), CDC14B (-2.1), CDC25A (-3.1), CDK6 (-3.0), CDK7 (-1.9), CENPJ (-3.0), CHORDC1 (-3.5), DIPK1A (-4.0), DMTF1 (-1.7), EGFR (-1.9), EIF2B2 (-1.5), EIF4E (-1.9), HACE1 (-1.7), HARS1 (-1.8), HSDL2 (-1.6), IFRD1 (-2.4), IGF1R (-1.6), IPO4 (-2.3), ITGA2 (-1.6), KIF23 (-2.6), LUZP1 (-1.8), MAP2K4 (-1.6), MLLT1 (-1.4), MSH2 (-2.8), NAA15 (-2.8), NOTCH2 (-2.3), PISD (-2.0), PLK1 (-3.0), PNN (-2.1), PNP (-2.5), PPIF (-1.9), PRIM1 (-2.6), PSAT1 (-3.6), RARS1 (-2.0), RECK (-2.7), RFT1 (-2.0), SLC25A22 (-2.4), SLC38A1 (-2.1), TMEM189-UBE2V1 (-2.8), TPM3 (-2.0), UBE2S (-2.8), UTP15 (-1.8) |
| hsa-miR-148b-3p  miR-148a-3p (w/seed CAGUGCA)  (>0.087%) | 4.0 | 47/50 | ABL2 (-2.0), ADAM10 (-1.7), C16orf70 (-2.7), CD274 (-2.6), CDC25B (-3.4), CDKN1A (-6.7), CEBPG (-1.8), CHD1 (-2.1), CLOCK (-2.0), COL4A1 (-13.6), CYTH3 (-11.4), DNMT1 (-2.2), DNMT3B (-5.5), EGFR (-1.9), EPAS1 (-5.5), FAM161A (-3.6), FOXK2 (-1.7), IGF2BP3 (-18.9), ITGA5 (-2.1), KLHL5 (-11.5), KRTAP2-3 (-236.2), LIPG (-6.9), MED12L (-4.6), MOSPD1 (-1.9), MTF1 (-1.4), MYBL1 (-4.6), NAA15 (-2.8), NPC1 (-2.5), NRP1 (-4.9), OTUD4 (-1.8), PIDD1 (-4.8), PKIB (-2.3), PRRG1 (-5.3), PSMC3IP (-3.4), PTPN14 (-1.7), RHOF (-2.80, RNF44 (-2.0), SESN2 (-3.0), SH2B3 (-2.8), SLC31A2 (-9.1), SMIM13 (-2.5), TAF1D (-2.4), TEAD1 (-1.9), TUBE1 (-1.5), UBA6 (-1.9), WNT10B (-24.9), ZNF488 (-11.7) |
| hsa-miR-30d-5p  miR-30c-5p (w/seed GUAAACA)  (>0.087%) | 3.1 | 41/46 | CARS1 (-2.1), CCN2 (-12.7), CEP76 (-2.9), CHD1 (-2.1), CHI3L1 (-939.7), COL4A1 (-13.6), DBF4 (-3.4), DCUN1D3 (-2.4), FAM72B (-5.5), FAM72C (-6.2), FAM72D (-5.6), FBXO45 (-1.6), GAN (-2.9), GTF2E2 (-1.9), HOXB8 (-3.9), IER5 (-2.8), IFRD1 (-2.4), ITGA2 (-1.6), MAP3K21 (-2.7), MICB (-5.0), NFAT5 (-1.4), NFATC3 (-2.7), NID1 (-29.5), OTUD4 (-1.8), PAWR (-2.7), PLEKHA8 (-2.2), POLR3G (-17.7), PRDM1 (-4.4), PRRG1 (-5.3), RHEBL1 (-5.1), S100PBP (-2.0), SALL4 (-17.5), SEMA6B (-9.2), SH2B3 (-2.8), SLC38A1 (-2.1), SLC4A7 (-4.3), SNAI1 (-13.9), TEAD1 (-1.9), UAP1 (-1.7), XPO1 (-2.6), YBX1 (-1.9) |
| hsa-miR-1287-5p  (w/seed GCUGGAU)  (>0.001%) | 9.4 | 26/26 | ALPG (-100.6), APOBEC3H (-9.1), CEP57L1 (-2.1), CLOCK (-2.0), DDB2 (-3.9), DDIAS (-3.1), EGFR (-1.9), GXYLT1 (-2.1), KIAA0040 (-3.3), LIMS4 (-3.3), MAP3K21 (-2.7), MPRIP (-2.3), MUC13 (-13.5), NUP153 (-1.8), PLEKHA8 (-2.2), PRIM2 (-2.1), PRSS35 (-70.8), PSIP1 (-2.3), PSRC1 (-1.8), RGS16 (-11.8), SHC1 (-2.3), SULT1C4 (-728.1), TEAD3 (-2.0), TLR4 (-4.9), YBX1 (-1.9), YBX3 (-3.8) |
| hsa-miR-3074-5p  (w/seed UUCCUGC)  (>0.001%) | 2.7 | 8/8 | DDIAS (-3.1), EML2 (-2.5), IGFL2 (-17.0), NEK2 (-2.0), PIMREG (-2.7), PMAIP1 (-5.8), RHOV (-4.7), SLC25A19 (-3.0) |
| hsa-let-7e-3p  (w/seed UAUACGG)  (>0.001%) | -3.7 | 2/25 | TMEM163 (8.5), ZNF503 (1.6) |
| hsa-let-7f-1-3p  let-7a-3p (w/seed UAUACAA)  (>0.001%) | -2.3 | 8/117 | APOL6 (6.0), BTG1 (5.2), KMT2E (2.3), LIX1L (8.6), PLPP1 (1.9), PNRC1 (3.1), TACR2 (21.6), TCP11L2 (4.3) |
| hsa-miR-125a-3p  (w/seed CAGGUGA)  (>0.001%) | -1.8 | 8/79 | APOL6 (6.0), BCKDHA (1.8), DAP (2.8), DNAJC22 (1.5), GGT6 (6.1),  KIF13B (2.4), KLK6 (7.0), LGALS3BP (10.0) |
| hsa-miR-1292-5p  miR-1247-3p (w/seed GGGAACG)  (>0.001%) | -1.9 | 3/54 | CAPN5 (5.2), DNAJC4 (1.8), TACR2 (21.6) |
| hsa-miR-942-5p  miR-12202-3p (w/seed CUUCUCU)  (>0.001%) | -1.9 | 6/60 | DTX1 (4.4), HIPK2 (2.7), PCDHB12 (18.0), TACR2 (21.6), TCP11L2 (4.3), ZBTB18 (1.8) |

*bolded miRs are predicted to be activated or inhibited as upstream regulators (z score ≥ 2 activated) or (z score ≤ -2

inhibited)

All miRs listed have expression changes with significant p values (<0.05).

(% total small reads mapped to hg38) The average %=0.087%

1. AGS-EBV T vs pc T

| hsa-miR* | Fold change | targets changed/targets in dataset | targets |
| --- | --- | --- | --- |
| **hsa-let-7b-5p**  let-7a-5p (w/seed GAGGUAG)  (>0.087%) | -2.1 | 67/72 | AMT (6.7), APBB3 (2.7), ARID3A (2.2), ARID3B (1.8), ARL4D (4.3), ATAD3B (1.9), ATXN7L2 (2.0), AURKB (2.1), AVEN (1.6), BLOC1S1 (1.5), C6orf141 (2.3), CARHSP1 (1.6), CDC25A (1.6), CDC42SE1 (1.8), COL1A1 (1.9), COL27A1 (2.1), COL4A1 (7.3), CRTAP (1.3), DDTL (2.1), DPF2 (1.6), DUSP22 (1.6), FAM118A (2.5),  FANCD2 (1.7), GOLGA6L9 (2.7), HMGA2 (2.8), IFRD1 (1.8), IGF2BP2 (1.7), IL10 (8.4), INTS6L (2.7), IQCB1 (1.7), MED28 (1.9), MLLT1 (1.2), MTRR (1.5), NEK3 (2.6), NR6A1 (2.0), PBX2 (1.9), PCTP (1.6), PDGFB (2.8), POLL (1.5), POLR2D (1.6), PRPF38B (1.6), RAB11FIP4 (1.8), RGS16 (1.9), RPP38 (1.3), RPUSD3 (1.6), RRP8 (1.4), SCN8A (2.3), SCYL3 (1.7), SLC1A4 (2.0), SLC25A32 (1.5), SMC1A (1.5), SMOX (2.2), SMUG1 (2.1), TARBP2 (2.1), THRA (1.3), TLR4 (2.7), TMEM234 (2.0), TRMT1 (1.7), TTLL4 (2.2), TUSC2 (1.5), UHRF2 (1.5), ZC3H3 (1.9), ZCCHC9 (1.7), ZNF226 (1.7), ZNF341 (1.6), ZNF436-AS1 (2.5), ZNF710 (1.2) |
| hsa-let-7b-3p  let-7a-3p (w/seed UAUACAA)  (>0.001%) | -2.4 | 4/6 | COA1 (1.6), IER5 (1.5), LYSMD4 (1.4), SCX (4.1) |
| hsa-miR-1910-5p  (w/seed CAGUCCU)  (<0.001%) | -5.2 | 16/20 | ATG10 (2.1), CDC42SE1 (1.8), DPM2 (1.7), FAM118A (2.5), GLDC (2.7), LPAR2 (1.5), LRRC37A3 (2.4), MEA1 (1.7), MFSD2A (1.7), MIS12 (1.9), PDGFB (2.8), POLR2D (1.6), RSAD1 (1.7), SELENOW (1.9), TULP3 (1.5), ZNF688 (2.2) |
| hsa-miR-193b-3p  miR-193a-3p (w/seed ACUGGCC)  (>0.001%) | -1.3 | 25/27 | ADAMTS13 (4.1), AIMP2 (1.5), ARID3B (1.8), C11orf49 (1.4), C19orf44 (1.8), CALM1 (1.9), CAMK2N2 (1.8), DEF8 (1.4), HEMK1 (1.9), IL17RD (3.3), ING5 (1.6), LYSMD4 (1.4), MSANTD2 (2.6), NT5DC3 (1.8), NT5M (1.9), POLR2J2 (1.9), /POLR2J3 (2.1), PSRC1 (2.4), RPS6KB2 (1.5), SENP5 (1.4), SMUG1 (2.1), SRSF2 (1.5), STX16 (2.5), TWISTNB (1.6), ZC3H10 (1.9) |
| hsa-miR-193a-5p  (w/seed GGGUCUU)  (>0.001%) | -2.1 | 14/14 | ARAP3 (2.0), CCDC159 (2.3), GAS7 (4.4), IL10 (8.4), KIF18A (1.9), NBPF10 (2.5), S100PBP (1.7), SHARPIN (2.3), SNX22 (2.1), TMEM234 (2.0), TNS4 (2.0), UBBP4 (1.6), UCN (4.6), ZNF875 (1.8) |
| hsa-miR-203b-5p  (w/seed AGUGGUC)  (>0.001%) | -3.5 | 6/7 | HIKESHI (2.3), METTL23 (1.5), NDUFA12 (1.7), PDX1 (1.8), RBM14 (1.5), TMEM218 (2.1) |
| hsa-miR-3065-5p  (w/seed CAACAAA)  (>0.001%) | -3.4 | 6/6 | CCL2 (27.9), COA1 (1.6), DFFB (1.5), SRSF7 (1.8), TM2D1 (1.5), TRMT112 (1.4) |
| hsa-miR-338-5p  (w/seed ACAAUAU)  (>0.001%) | -8.1 | 5/5 | CCDC14 (2.6), DYRK4 (1.7), FAM72A (1.5), RAB28 (1.8), RBX1 (1.5) |
| hsa-miR-33b-3p  miR-515-3p (w/seed AGUGCCU)  (>0.001%) | -2.6 | 8/9 | PFN4 (3.6), POLR2D (1.6), RNF43 (2.0), TBC1D3 (13.3), TMEM138 (1.9), TMEM218 (2.1), ZNF202 (1.6), ZNF513 (1.7) |
| hsa-miR-345-5p  (w/seed CUGACUC)  (>0.001%) | -1.8 | 4/5 | AIMP2 (1.5), DNAH10OS (3.5), NMRK1 (2.2), ZNF133 (1.8) |
| hsa-miR-4516  miR-4434 (w/seed GGAGAAG)  (>0.001%) | -3.3 | 17/18 | ARF3 (1.6), CDRT4 (10.5), CPOX (1.6), FAM227A (2.3), IFNE (8.0), ING4 (2.1), MIEN1 (1.4), MSL1 (1.4), NGLY1 (1.5), PAK1 (1.6), PDX1 (1.8), RNF10 (1.3), S1PR2 (1.4), SPATA33 (2.1), TADA2A (1.7), VPS72 (1.4), ZNF133 (1.8) |
| hsa-miR-4707-5p  (w/seed CCCCGGC)  (<0.001%) | -7.6 | 24/24 | APOBEC3F (2.0), C15orf62 (2.8), CFAP298 (1.8), COX19 (2.0), DMPK (2.7), DTX3 (2.0), ENGASE (2.1), EPOR (4.0), GMPPA (1.5), H2AX (2.0), HOXC6 (3.7), IER5 (1.5), IP6K2 (1.6), LIMD1 (1.5), MELTF (1.9), MIER2 (1.5), NAP1L4 (1.7), PPFIA3 (1.7), RHEBL1 (2.1), RHOT2 (1.7), SEMA6B (5.2), SOCS2 (2.4), ST3GAL2 (4.0), THAP3 (1.8) |
| hsa-miR-4749-3p  (w/seed GCCCCUC)  (<0.001%) | -3.5 | 38/38 | AGER (5.79), ANKRD52 (1.4), ATXN7L3 (1.4), DMTN (1.9), DNAJB2 (1.8), DOK1 (1.4), EFEMP2 (2.9), EML3 (1.7), ETV1 (2.2), FURIN (2.7), GDF11 (1.9), GIGYF1 (1.7), HARS2 (1.6), HDAC11 (2.3), LAGE3 (1.5), LMBR1L (2.3), LYSMD4 (1.4), MAZ (1.3), METRN (1.4), MRPL20-AS1 (1.7), MTA3 (1.7), MTSS2 (2.6), NFIX (1.3), PIAS4 (1.4), POLR2J2 (1.9), POLR2J3 (2.1), POMGNT1 (1.5), PRKAG1 (1.7), PRPF38A (1.4), QTRT1 (1.7), SNX22 (2.1), SPRYD3 (1.3), SRCIN1 (3.5), THRA (1.3), TMEM216 (1.8), ULK3 (1.9), WBP1 (2.7), ZNF502 (1.8) |
| hsa-miR-501-3p  (w/seed AUGCACC)  (>0.001%) | -1.5 | 2/4 | CCR1 (4.1), SLC26A6 (2.1) |
| hsa-miR-106b-5p  miR-17-5p (w/seed AAAGUGC)  (>0.087%) | 1.9 | 2/15 | CRYBG3 (-1.6), FGD4 (-1.7)  [targets not downregulated - ANKRD52, APCDD1, BLACAT1, CAMK2N2, CAPRIN2, FAM219B, FICD, LIN7B, MAP11, MRPL24, PXK, TMEM138, TUSC2] |
| hsa-miR-1296-5p  (w/seed UAGGGCC)  (>0.001%) | 2.2 | 1/13 | SNX29 (-1.4)  [targets not downregulated - C17orf67, CTC1, DPF2, HMGA2-AS1, KANSL2, LZTR1, PLEKHB1, RUSF1, SRSF2, TMEM107, UNC119, WNT5B] |
| hsa-miR-1306-5p  (w/seed CACCUCC)  (>0.001%) | 1.7 | 3/21 | AREL1 (-1.6), COL17A1 (-4.9), TACR2 (-7.2)  [targets not downregulated - AZGP1, BTN2A2, DNASE1, EIPR1, GOLGA7B, HRAS, IL17RD, MC1R, METRN, NASP, NR6A1, PABPN1, SUDS3, TRMT112, UNC119B, ZFP41, ZNF589, ZNF841] |
| hsa-miR-142-3p  (w/seed GUAGUGU)  (>0.001%) | 4.5 | 2/11 | ABCD1 (-1.9), MARCKS (-1.6)  [targets not downregulated - COG4, HMGA2, MRFAP1, NR2C1, PPP1R37, RGL2, SEC22A, SLC1A3, ZFYVE27] |
| hsa-miR-144-5p  (w/seed GAUAUCA)  (>0.001%) | 8.7 | 2/5 | ANG (-3.3), SYNPR (-3.4)  [targets not downregulated - GOLGA7B, PIN4, ZNF700] |
| hsa-miR-148b-3p  miR-148a-3p (w/seed CAGUGCA)  (>0.087%) | 1.6 | 2/13 | ELF5 (-7.5), GLRX5 (-1.5)  [targets not downregulated - DNMT1, EMG1, HOXC8, MIGA2, MRPL28, NEURL4, PABPC1L, TNRC6A, TUBE1, VMP1, ZNF226] |
| hsa-miR-15b-3p  (w/seed GAAUCAU)  (>0.001%) | 2.0 | 2/6 | GLRX5 (-1.5), KLF13 (-1.60)  [targets not downregulated - HIKESHI, STRADB, TBCE, TEFM] |
| miR-205-5p (w/seed CCUUCAU)  (>0.087%) | 9.1 | 1/8 | FMN1 (-3.0)  [targets not downregulated - ABI2, CISD3, HS3ST1, INPPL1, NACC2, NDUFA4, ZNF23] |
| hsa-miR-301a-3p  miR-130a-3p (w/seed AGUGCAA)  (>0.001%) | 2.5 | 6/20 | ARHGAP24 (-3.6), ATG2B (-1.8), DICER1 (-1.7), GAREM1 (-2.1), KLF3 (-2.2), TRERF1 (-2.0)  [targets not downregulated - APCDD1, CALM1, CENPO, FICD, IL10RB, LRP8, MEIS1, PHF14, PXK, SLC25A32, SRSF2, STK33, STX6, TSC1, ZNF3] |
| hsa-miR-301a-5p  (w/seed CUCUGAC)  (>0.001%) | 2.1 | 1/2 | GLRX5 (-1.5)  [targets not downregulated - KRBOX4] |
| hsa-miR-339-3p (w/seed GAGCGCC)  (>0.001%) | 1.5 | 1/14 | SOD3 (-11.2)  [targets not downregulated - ABHD17A, ARFGAP2, CST1, IGHMBP2, ING5, IP6K2, KMT5A, LBX2, MRPL20-AS1, TAOK2, TCEANC2, TOLLIP, TUBGCP6] |
| hsa-miR-339-5p  (w/seed CCCUGUC)  (>0.001%) | 1.7 | 1/14 | APOL6 (-3.01)  [targets not downregulated - C15orf62, COPZ1, CPLX2, FAM219B, GABARAPL1, MFSD13A, NIF3L1, RFC5, TLE3, TRAPPC2, ZNF397, ZNF589, ZNF771] |
| hsa-miR-421  miR-421-3p (w/seed UCAACAG)  (>0.001%) | 1.3 | 3/13 | KRT6B (-6.1), SIX4 (-242.5), SPRY1 (-1.8)  [targets not downregulated - ARL17A/ARL17B, ERLIN1, GEMIN6, MPV17, SEC11C, TBC1D3, TPK1, UTP3, VPS29, ZNF337] |
| hsa-miR-424-5p  miR-16-5p (w/seed AGCAGCA)  (>0.001%) | 3.0 | 11/55 | AREL1 (-1.6), CBX4 (-1.5), EGFR (-1.7), FGF18 (-2.6), GAREM1  (-2.1), GFPT1 (-1.9), ITGA2 (-1.7), RPS6KA3 (-1.8), SLC12A2 (-3.7), TOGARAM1 (-1.8), SEC24A (-1.6)  [targets not downregulated - ATXN2, ATXN7L3, CDC25A, CENPJ, CFAP45, CHORDC1, DMTF1, DNAJB4, ELL2, FAM133B, GABARAPL1, GABBR1, HMGA2, HPF1, IFRD1, IKBKB, LARGE2, MLLT1, MTMR11, NFS1, PISD, PLPP1, PMS1, PPT2, PRIMPOL, PTCD3, RAD51C, RBM6, SCN8A, SMURF2, SPRYD3, STRADB, TARBP2, TIA1, TMEM258, TRMT112, TRMT13, UCP2, WDR83OS, ZBTB46, ZDHHC23, ZNF609, ZNF691, ZNF697] |
| hsa-miR-4443  (w/seed UGGAGGC)  (>0.001%) | 2.3 | 5/33 | CKMT1A/CKMT1B (-6.6), CLDN18 (-337.5), IL1RN (-12.4), KLF13 (-1.6), PRPS2 (-1.5)  [targets not downregulated - AKT2, APOBEC3C, ATXN2, BCL11A, C1orf74, DPF2, EXOG, EXOSC2, FBXL18, GBA2, HEPACAM, IRF3, KLHDC4, LDB1, LZTR1, MRPL20-AS1, NECAB3, NR6A1, PCGF3, POLR2D, PRR3, PRRT1, PRSS33, PYROXD2, RABEP2, SPC24, TESK1, TRAPPC2] |
| hsa-miR-454-5p  (w/seed CCCUAUC)  (>0.001%) | 1.8 | 1/7 | TLN2 (-2.7)  [targets not downregulated - ANKRD10, DUSP14, FOXD4, LMBR1L, MARS1, RAD54L] |
| hsa-miR-486-3p  (w/seed GGGGCAG)  (>0.001%) | 5.6 | 7/112 | AATK (-3.1), CDC42EP1 (-1.9), FGF18 (-2.6), IRF2BPL (-1.4), JDP2 (-67.7), KLF13 (-1.6), ZFHX3 (-1.5)  [targets not downregulated - ANKRD52, ANKRD54, ARF3, ASB16, ATXN7L3, AURKB, CCDC106, CCDC114, CDA, CDC42SE1, CENPO, CLDN15, CLIP3, CNTNAP1, CPLX2, CPSF7, CYB5RL, DBF4B, DDX11, DDX56, DHRSX, DMTN, DNAH10OS, DTX3, E2F4, EIF2AK1, ENSA, ENTPD2, EPOR, FAM53A, FIZ1, GABBR1, GDPD5, HAUS5, HCN3, HEMK1, HGH1, HOXC6, HOXC8, HROB, HS6ST1, IP6K2, ITGA5, KIF21B, KSR1, LARGE2, MAP3K10, MBD3, MIB2, MTA3, MXRA8, NACC2, NDOR1, NDUFA11, NR6A1, NXF1, ORAI3, PABPN1, PIGQ, PLA2G6, PLEKHO2, POLL, POLR2D, POLR2F, POLR2J2/POLR2J3, PRRT2, PYCR3, RAB11FIP4, RBCK1, RDH5, SAMD1, SCN1B, SCN8A, SCRIB, SGSM3, SH3GLB2, SLC16A8, SLC7A5, SMOX, SOX13, SPATA33, SPRYD3, SRSF6, SUFU, TBC1D24, TCEANC2, THRA, TLE5, TMCC2, TMEM234, TMEM91, TP53I3, TULP3, TXLNA, U2AF1L4, UNC119B, UQCC2, WNT5B, ZDHHC8, ZFYVE28, ZMIZ2, ZNF436-AS1, ZNF444, ZNF502, ZNF710] |
| hsa-miR-498-5p  (w/seed UUCAAGC)  (>0.001%) | 23.4 | 1/4 | PRDX4 (-1.7)  [targets not downregulated - ACYP1, RAB28, SF3B6] |
| hsa-miR-499a-5p  miR-499-5p (w/seed UAAGACU)  (>0.001%) | 4.0 | 1/2 | LGMN (-3.1)  [targets not downregulated - C12orf73] |
| hsa-miR-516a-5p  (w/seed UCUCGAG)  (>0.001%) | 5.8 | 1/6 | CREBBP (-1.7)  [targets not downregulated - EXOSC2, FAM220A, LHX1, LINC01006, SUPT4H1] |
| hsa-miR-516b-5p  (w/seed UCUGGAG)  (>0.001%) | 40.9 | 3/15 | DGKG (-3.3), IL1RN (-12.4), SYNPR (-3.4)  [targets not downregulated - BGLAP, CCDC167, KNSTRN, KRBOX4, MTMR11, NDUFA11, PHYKPL, PPP1R12B, SRRM2, TCEANC2, UBBP4, ZNF250] |
| hsa-miR-517a-3p  (w/seed UCGUGCA)  (>0.001%) | 8.1 | 2/6 | NFIB (-2.2), SNX18 (-2.1)  [targets not downregulated - C12orf65, MYH4, NUP42, SH3TC1] |
| hsa-miR-106b-3p  (w/seed CGCACUG)  (0.001%) | 1.5 | 0/8 | [targets not downregulated - ADAMTS13, C11orf45, DNAH10OS, LAGE3, MRPS21, PUF60, RNFT2, SIGLEC15] |
| hsa-miR-1283  (w/seed CUACAAA)  (0.001%) | 5.2 | 0/3 | [targets not downregulated - ANKRD10, CDK11A, SUFU] |
| hsa-miR-144-3p  (w/seed ACAGUAU)  (>0.001%) | 7.4 | 0/6 | [targets not downregulated - MPHOSPH6, PFDN6, PLAT, PLPP5, ZHX1, ZNF493] |
| hsa-miR-372-5p  (w/seed CUCAAAU)  (0.001%) | 1.9 | 0/4 | [targets not downregulated - CDC45, LBX2, SEC11C, YAF2] |
| hsa-miR-522-3p  (w/seed AAAUGGU)  (>0.001%) | 3.2 | 0/8 | [targets not downregulated - ATP23, BANF1, DHFR, DNAJC19, GTF2H5, PYCR2, TMEM91, UBE2N] |

*bolded miRs are predicted to be activated or inhibited as upstream regulators (z score ≥ 2 activated) or (z score ≤ -2

inhibited)

All miRs listed have expression changes with significant p values (<0.05).

(% total small reads mapped to hg38) The average %=0.087%

[targets not downregulated]

1. BART T vs pc T

| hsa-miR* | Fold change | targets changed/targets in dataset | targets |
| --- | --- | --- | --- |
| hsa-miR-1257  (w/seed GUGAAUG)  (<0.001%) | -3.8 | 77/79  (71/79 if ≥1.3 FC) | ALDOB (2.3), ANK3 (1.3), ARL13B (1.4), BMP2K (1.4), BTF3L4 (1.3), CBLB (1.4), CDK6 (1.6), CERS6 (2.1), CFDP1 (1.6), CRACD (1.3), CSNK1G1 (1.3), CTC1 (1.3), DMTF1 (1.4), DNAJB4 (2.0), DNAJC6 (1.3), EMSY (1.4), ERH (1.3), EXOSC2 (1.3), FAM13A (1.4), FAM3C (1.2), FER (1.7), FYTTD1 (1.3), GALNT4 (1.3), GASK1B (2.9), GMDS (1.3), GSPT1 (1.2), GTF2F2 (1.8), GZF1 (1.4), H3-3A (1.2), H3-3B (1.3), HACD2 (1.3), IFT46 (1.5), INPP1 (1.4), LRRC31 (2.0), LYPD6 (1.8), LYRM1 (1.5), MOB3B (2.2), MRPL20-AS1 (1.4), MTFR1 (1.2), MYNN (1.3), NAB1 (1.5), NUDT17 (1.5), NUP37 (1.5), PAN3 (2.1), PAPSS1 (1.5), PHYH (1.4), PIGO (1.3), PIK3C2A (1.3), REEP3 (1.3), RNF14 (1.3), RPUSD2 (1.2), SAMD5 (2.9), SAYSD1 (1.4), SDHAF3 (1.3), SKP1 (1.2), SMC1A (1.2), SOX12 (1.4), SRSF7 (1.3), STX18 (1.9), SVIL (1.6), TASOR (1.6), TTLL11 (1.5), UBTD2 (1.3), UGT8 (1.6), VAPB (1.4), VKORC1L1 (1.3), XRCC4 (1.6), YES1 (1.3), ZCCHC17 (1.4), ZFX (1.4), ZNF117 (2.4), ZNF17 (1.3), ZNF23 (1.3), ZNF286A (1.3), ZNF543 (1.4), ZNF544 (1.4), ZNF584 (1.3), ZNF808 (1.3) |
| hsa-miR-18a-5p  (w/seed AAGGUGC)  (>0.001%) | -1.6 | 85/87  (70/87 if≥1.3 FC) | ARPIN (1.4), ARPIN-AP3S2 (1.3), ASXL2 (1.3), ATP9B (1.2), AXIN2 (1.8), BBX (1.4), BMP2K (1.4), BRWD3 (1.3), C7orf50 (1.2), CAD (1.3), CHTF8 (1.3), CTF1 (1.8), DCAF7 (1.4), DFFA (1.4), DNAJC8 (1.3), EHMT1 (1.1), ENC1 (2.0), ERBB2 (1.2), ERLIN1 (1.4), ESCO2 (1.3), FAM229B (1.8), FAM3C (1.2), FANCG (1.4), FCHSD2 (1.3), FER (1.7), GAB1 (1.5), GABPA (1.3), GINS1 (1.4), HEATR5A (2.1), HHLA3 (2.1), HIF1AN (1.3), HMGN2 (1.4), HOMER1 (1.3),  IFT140 (1.4), IL10RB (1.3), IRF2 (1.5), ITM2B (1.6), KDM5B (1.3), KPNA6 (1.2), LRRC73 (2.1), LSM14B (1.4), MACIR (1.3), MANEAL (2.1), MYO5C (1.2), NKIRAS1 (1.4), ORAI3 (1.3), PEX11A (1.4), PIK3C2A (1.3), POLR2D (1.3), PRKACB (2.0), PRXL2B (1.2), RABGAP1 (1.3), RNF145 (1.3), RNF187 (1.2), RNF4 (1.6), RPL7L1 (1.3), RRAS (1.7), RSU1 (1.3), RTL6 (1.4), SCLY (1.5), SDHAF3 (1.3),  SLX4IP (2.1), SNX29 (1.1), SOX12 (1.4), STK17A (1.4), SUCO (1.2),  TAOK1 (1.3), TKFC (1.3), TMEM230 (1.4), TMEM254 (1.7), TOR1B (1.3), TRAPPC8 (1.2), TRIQK (1.6), TTPAL (1.8), UBTD2 (1.3), USP24 (1.2), VPS13A (1.4), VPS52 (1.3), WDR82 (1.2), WNT8B (1.6), ZFP62 (1.5), ZNF286A (1.3), ZNF341 (1.2), ZNF501 (1.7), ZNF589 (1.4), ZNF627 (1.6) |
| hsa-miR-193b-3p  miR-193a-3p (w/seed ACUGGCC)  (>0.001%) | -1.6 | 111/119  (95/119  if ≥1.3 FC) | ADAMTS13 (1.7), AGTPBP1 (1.3), ANKFY1 (1.2), ANKRD54 (1.3), ARFIP1 (1.3), ARHGEF12 (1.3), ATG7 (1.4), ATXN3 (1.3), BDP1 (1.4), C11orf49 (1.2), C19orf44 (1.8), C9orf85 (1.3), CALM1 (1.2), CAMK2N2 (1.4), CBX1 (1.6), CCDC127 (1.4), CCDC186 (1.3), CENPS (1.4), CEP41 (1.9), CHTF8 (1.3), CLPB (1.4), COPS3 (1.2), DCAF7 (1.4), DDAH1 (1.2), DENND2B (1.4), DIPK1A (1.6), DLG1 (1.7), DOK7 (1.7), EBAG9 (1.5), ELMO2 (1.6), ENKD1 (1.5), FARSA (1.3), FCHSD2 (1.3), GAMT (1.5), GPALPP1 (1.6), GRHL2 (1.4), GSDMD (5.3), HACD2 (1.3), HELZ (1.2), HOXC6 (2.9), IMMP2L (1.7), JMY (1.3), KANK2 (1.3), KAZALD1 (1.8), KHDC4 (1.3), MARCKSL1 (1.4), MARF1 (1.2), MAX (1.2), MGAT3 (2.0), MKLN1 (1.3), MKS1 (1.3), MSANTD2 (1.3), MTF1 (1.2), MTHFD1 (1.2), NDOR1 (1.2), NF1 (1.5), NSF (1.3), NUDT15 (1.4), NUDT18 (1.5), OGG1 (1.4), PAFAH2 (1.6), PCYOX1L (1.8), POGK (1.4), POLR3H (1.4), POMK (1.3), PREB (1.3), PRR14L (1.3), PRR15L (1.4), PSRC1 (1.3), PXYLP1 (1.4), RALGAPB (1.5), RBMXL1 (1.3), RNF144B (1.7),  RPL17-C18orf32 (1.6), RPS6KA1 (1.3), RPS6KB2 (1.2), RSF1 (1.3), SAYSD1 (1.4), SCYL3 (1.5), SKAP2 (1.6), SLC9A7 (1.3), SLF2 (1.8), SMIM14 (1.3), SNAPC1 (1.3), SNX1 (1.3), SNX27 (1.3), SOS2 (1.3), SOX12 (1.4), SP4 (1.6), SPECC1L (1.4), SRSF6 (1.3), TAOK1 (1.3),  TIMM8B (1.3), TMEM216 (1.5), TMEM30B (1.3), TMEM43 (1.3), TPM2 (3.1), TTC5 (1.3), TTPAL (1.8), USP40 (1.6), USP53 (1.3), UTP18 (1.3), WDR6 (1.2), WDR82 (1.2), WDR92 (1.6), ZBTB5 (1.2), ZNF248 (1.6), ZNF37A (1.3), ZNF510 (1.3), ZNF638 (1.6), ZNF646 (1.1) |
| **hsa-miR-449c-5p**  miR-2682-5p (w/seed AGGCAGU)  (<0.001%) | -2.9 | 80/82  (70/82 if ≥1.3 FC) | ADORA2B (1.4), AP4S1 (1.6), APPL1 (1.2), ARRDC3 (1.7), ATXN7L3B (1.5), BMP7 (2.8), C1orf131 (1.3), C2orf15 (1.5), C6orf141 (2.3), CALD1 (1.7), CASP6 (1.6), CBLB (1.4), COG3 (1.5), CREB1 (1.4), CREB3L1 (2.0), CRIP2 (3.1), CTF1 (1.8), DAAM1 (1.5), DAGLB (1.3), DDX50 (1.4), DNAJB2 (1.2), EPHA4 (1.9), ERLIN1 (1.4), FAM3C (1.2), FANCE (1.3), FBXO34 (1.3), FGFRL1 (1.9), FNBP1L (1.5), FUT10 (1.5), GOLGA4 (1.3), GPALPP1 (1.6), HACD2 (1.3), HMBS (1.3), HNF4G (1.6), IL10RB (1.3), ISG20L2 (1.2), LHPP (1.4), LYZ (1.4), MAMSTR (1.5), MCPH1 (1.3), MET (1.6), MPV17 (1.5), MTFR1 (1.2), MTMR6 (1.6), NBR1 (1.3), NCOA4 (1.3), NUDT16L1 (1.2), NUP153 (1.2), PDS5A (1.3), PIAS1 (1.3), PIGO (1.3), PIK3C2A (1.3), PLEKHA8 (1.4), PREB (1.3), PRR3 (1.4), PYGB (1.5), QDPR (1.7), RAB8B (1.4), RANBP10 (1.2), RBBP5 (1.4), RIC8B (1.4), RNF144B (1.7), RNF4 (1.6), RPL7L1 (1.3), RPP25L (1.7), RTL6 (1.4), RUSF1 (1.3), SKP1 (1.2), SLC19A2 (1.4), SLC30A6 (1.5), SLC39A9 (1.2), SPINK5 (1.6), STEAP2 (2.2), STK38L (1.5), SURF1 (1.3), TGFBR2 (1.6), TLR4 (1.9), TMCC1 (1.4), TMED8 (1.3), TNPO1 (1.3) |
| hsa-miR-4632-3p  miR-1900 (w/seed GCCGCCC)  (<0.001%) | -12.5 | 58/60  (41/60 if ≥1.3 FC) | ABHD15 (1.4), ADAMTS13 (1.7), AKAP13 (1.3), C17orf80 (1.5), CAMK2N2 (1.4), CARM1 (1.2), CCM2 (1.4), CELF1 (1.1), CENPS (1.4), CHD8 (1.3), DNAJC22 (1.2), DNM2 (1.2), ENTPD6 (1.3), ETHE1 (2.0), FAHD1 (1.4), GRAMD4 (1.4), H2AJ (1.3), HTT (1.8), KAZALD1 (1.8), KMT2C (1.1), LCMT2 (1.6), LRRC56 (1.3), LTBP4 (1.4), MAD1L1 (1.2), MARK2 (1.2), MEX3D (1.2), MTHFSD (1.5), NFIX (1.2), NRBP1 (1.2), NUDT18 (1.5), PCDHGA1 (2.0), PCDHGA6 (2.5), PCGF3 (1.6), PHF21A (1.3), PIAS4 (1.2), POLR2D (1.3), PRKCZ (1.2), PRXL2B (1.2), PYCR3 (1.4), SERTAD4 (1.4), SHANK2 (1.4), SLC26A1 (1.8), SNAP47 (1.3), SRCIN1 (2.0), STMN3 (2.0), TMEM184A (1.5), TMEM43 (1.3), TNKS (1.2), TRIM15 (1.9), TSSK6 (1.3), VAPB (1.4), VPS52 (1.3), WDR24 (1.3), WDR82 (1.2), ZMIZ1 (1.2), ZNF497 (1.6), ZNF74 (1.2), ZNF747 (2.3) |
| **hsa-let-7b-5p**  let-7a-5p (w/seed GAGGUAG)  (>0.087%) | 1.5 | 2/175 | GLRX (-1.8), PI4K2B (-1.6)  [targets not downregulated - ABL2, ACER2, ACVR2B, AGO3, AHCTF1, ALKBH1, APBB3, APPBP2, ATG10, ATP13A3, ATXN7L3B, BDP1, BLOC1S5, BMP2K, BRD3, BRWD3, BTBD3, BTF3L4, C19orf53, C5orf51, C6orf141, CALM1, CCDC141, CDK6, CDK8, CDKAL1, CEP164, CERT1, CHCHD1, CHD7, CLASP2, CREB3L4, CRTAP, DAD1, DDTL, DDX19A, DICER1, DLC1, DPF2, DSP, EDEM3, ELP1, EPHA4, ERCC4, FAM135A, FAM189B, FAM72A, FAM72B, FAM72C/FAM72D, FANCD2, FBXO30, FKBP3, FNDC3A, FSD1L, FZD4, GABPA, GALE, GALNT1, GATM, GLB1, GMPR2, GNPTAB, GOLGA4, GTF2I, GYS1, HIPK2, HOOK1, HSPE1-MOB4, IFRD1, IKZF2, IPO4, IQCC, ITSN1, KCNE3, KIAA0895, KIF27, LGR4, LMLN, LOXL3, LRRC20, MED28, MEIS1, MEIS2, MGAM2, MIOS, MLLT1, MOCS3, MRPS33, MTUS1, NARS2, NAT14, NME6, NUDT15, NUP155, NXN, P4HA2, PAFAH2, PCGF3, PIAS4, PKN2, PLEKHA8, PLSCR3, PM20D2, POC1A, POGZ, POLQ, POLR2D, POLR3D, PPP2R2A, PPP3CA, PRIM2, PRKAB2, PRKAR2A, PRPF38B, PRRC2A, RAB8B, RABL2B, RB1, RGPD4, RMI2, RPP38, RPUSD2, RPUSD3, RRP8, S100PBP, SBK1, SCYL3, SLC30A4, SLC30A6, SLF2, SMC1A, SPATA7, SPTSSB, ST7L, STEAP2, STRBP, SUCLG2, SWSAP1, TADA2A, TECPR2, TET2, TGDS, TGFBR2, THYN1, TIMM17B, TLR4, TMED10, TMEM138, TMEM65, TNIK, TPM2, TRMT1, TTC26, TTC9C, TTL, UGT8, UHRF2, USP21, USP47, VSNL1, ZBTB5, ZC3HAV1L, ZCCHC9, ZNF226, ZNF22-AS1, ZNF248, ZNF329, ZNF341, ZNF343, ZNF410, ZNF583, ZNF599, ZNF765] |
| hsa-miR-1287-5p  (w/seed GCUGGAU)  (>0.001%) | 1.5 | 1/103 | ADM2 (-2.7)  [targets not downregulated – ABR, ARMCX6, BCL2L13, BCL2L14, BLOC1S5, BMPR2, C14orf119, C5orf51, CASP10, CCDC125, CCDC137, CCDC142, CEP63, CHTF8, CNDP2, COG8, CUX1, CXXC1, DAD1, DCAF10, DDAH1, DDX10, DHFR2, DHRS4, DHRS4L2, DLEU1, EGFR, ENTPD2, FAM168A, FAM71E1, FLT3LG, FUT2, GARRE1, GINS1, H2AX, HOMER1, HSPA4, IFT22, JPT2, KHK, L2HGDH, LCMT2, LGR4, LONP2, LSM6, LTBP4, MAF1, MAMDC4, MAVS, MBIP, MRPS10, MTERF3, MUC13, MYO9A, NIPBL, NMD3, NUP153, NUP188, OCIAD1, ORC2, ORC5, PAX9, PKP2, PLEKHA8, PLEKHG4, POGLUT1, PPP2R5E, PRIM2, PRKAR1A, PRPF38A, PRR3, PSIP1, PSRC1, RAB23, RIC8B, RNF41, RUFY1, SAYSD1, SDHAF3, SEPTIN11, SLC27A3, SLC29A4, SLC39A9, SNX29, SOAT1, SOX12, TBC1D3, THAP11, TLR4, TMEM128, TOGARAM1, TRABD2A, TTC5, UTP18, WDR92, XBP1, ZBED8, ZCCHC17, ZNF17, ZNF429, ZNF584, ZNF790 |
| hsa-miR-185-5p  (w/seed GGAGAGA)  (>0.001%) | 1.3 | 9/141 | CYFIP1 (-1.7), GCKR (-2.1), GLRX (-1.8), PLBD2 (-1.2), PLEKHF1 (-1.8), TANC2 (-1.3), VDR (-2.5), ZBTB7C (-1.4), ZCCHC4 (-1.6)  [targets not downregulated - AHSA1, ARID1A, BCL11B, BCL2L13, BTBD3, C5orf51, CALM1, CBY1, CCDC102A, CDK6, CHCHD1, CLBA1, CPT1A, CTF1, CYB5D1, CYP4V2, DCAF7, DHFR2, DNAJB4, DPF2, DSC2, EHBP1, EHMT2, ELP5, ENC1, EPHA4, ERCC4, ERG28, FBXO46, FIZ1, FNTB, FSCN2, FZD4, GASK1B, GDA, GINS1, GSTO2, GTF3C4, HNRNPA0, HOXC6, HS1BP3, ID3, INTS5, KANK2, KAZALD1, KSR1, LIG3, LYSMD1, MAD1L1, MEIS1, MOB3A, MOB3B, MRPL45, MRPS11, MSH3, MSX2, NFIX, NUDT18, NXF1, OCEL1, PAK2, PEX11A, PGAP4, PITPNC1, PLEKHA8, PLS3, PLSCR3, POGK, POT1, PRIM2, PRKAB2, PRKAR2A, PRMT6, PRUNE1, PRXL2B, PTPN13, RAB28, RBM6, REG4, RIC8B, RNF145, RRNAD1, RSBN1, RSU1, SAMD1, SBK1, SET, SGMS1, SLC30A6, SLC39A14, SMCO4, SMG7, SNAPC3, SNRPG, SNX27, SOX12, SRCIN1, SREBF2, SUCLG2, SUMF1, SUMF2, TAF8, TEX264, THTPA, TMEM139, TMEM161A, TMEM218, TOX3, TRIM5, TTC5, UBE2H, UBL7, UBQLN4, UBXN7, UCP2, VAPB, VIPAS39, WDCP, WDR7, WNT5B, WNT8B, XRCC3, ZBTB45, ZBTB5, ZFP41, ZMIZ1, ZNF226, ZNF37A, ZNF569, ZNF586, ZNF620, ZNF75D] |
| hsa-miR-1180-3p  (w/seed UUCCGGC)  (0.001%) | 1.6 | 0/26 | [targets not downregulated - C5, CEP41, DNAJC22, EFNA2, GINS4, ID3, MFAP1, MLLT1, MOB3A, MRPL20-AS1, NCOA4, NDOR1, NUDT16L1, PABPN1, PPP2R5E, RPS6KA1, RUFY1, TMEM254, TMTC2, TTLL11, WDR1, ZFP41, ZNF341, ZNF584, ZNF653, ZSWIM9] |

*bolded miRs are predicted to be activated or inhibited as upstream regulators (z score ≥ 2 activated) or (z score ≤ -2

inhibited)

All miRs listed have expression changes with significant p values (<0.05).

(% total small reads mapped to hg38) The average %=0.087%

[targets not downregulated]

1. Common AGS-EBV and BART T vs pc T

| hsa-miR* | Fold change | targets changed/targets in dataset | targets |
| --- | --- | --- | --- |
| hsa-miR-1257  (w/seed GUGAAUG)  (<0.001%) | -3.2 | 12/12 | CTC1 (1.6), DMTF1 (2.6), DNAJB4 (2.1), EXOSC2 (1.7), LYRM1 (2.0), MRPL20-AS1 (1.7), NUDT17 (2.0), NUP37 (2.0), SMC1A (1.5), SRSF7 (1.8), STX18 (2.1), ZNF23 (1.6) |
| hsa-miR-1910-5p  (w/seed CAGUCCU)  (<0.001%) | -3.5 | 25/25 | AP4M1 (1.6), ATG10 (2.1), CALM1 (1.9), CCDC58 (1.8), DNAJB4 (2.1), DNAJC22 (2.3), DOK1 (1.4), DPM2 (1.7), ELP6 (1.7), IP6K2 (1.6), LRRC37A3 (1.7), MIS12 (1.9), MOCS2 (1.5), MRFAP1L1 (2.3), MTFR1L (1.6), NDOR1 (1.9), NMRK1 (2.2), PABPN1 (1.7), POLR2D (1.6), RAB28 (1.8), SELENOW (1.9), TSPAN5 (2.7), TULP3 (1.5), ZNF688 (2.2), ZNF692 (3.5) |
| hsa-miR-193b-3p  miR-193a-3p (w/seed ACUGGCC)  (>0.001%) | -1.4 | 30/31 | ADAMTS13 (4.1), ANKRD54 (1.6), C11orf49 (1.4), C19orf44 (1.8), CALM1 (1.9), CAMK2N2 (1.8), CCDC127 (1.8), DENND2B (1.8), DOK7 (2.7), ENKD1 (1.7), GAMT (2.2), HOXC6 (3.7), KANK2 (1.5),  KHDC4 (1.8), MKS1 (1.3), MOCS2 (1.5), MSANTD2 (2.6), NDOR1 (1.9), PREB (1.3), PSRC1 (2.4), RPS6KB2 (1.5), SCYL3 (1.7), SNX1 (1.5), SPECC1L (1.3), SRSF6 (2.1), TIMM8B (1.5), TMEM216 (1.8), WDR6 (1.4), ZNF248 (3.0), ZNF638 (1.5) |
| hsa-miR-449c-5p  miR-2682-5p (w/seed AGGCAGU)  (<0.001%) | -2.0 | 22/23 | C6orf141 (2.3), DAGLB (1.7), DDX50 (1.5), DNAJB2 (1.8), ERLIN1 (1.5), FGFRL1 (2.2), IL10RB (1.9), MPV17 (1.9), PIGV (1.9), PREB (1.3), PRR3 (1.7), QDPR (1.9), RANBP10 (1.7), RIC8B (1.9), RUSF1 (1.6), SURF1 (1.5), TLR4 (2.7), TOMM6 (1.6), VPS52 (1.5), WBP1 (2.7), ZMYM3 (1.8), ZNF226 (1.7) |
| hsa-miR-4516  miR-4434 (w/seed GGAGAAG)  (<0.001%) | -2.3 | 34/35 | AASDH (1.5), ADAMTS13 (4.1), ANKRD26 (2.2), CPOX (1.6), DHFR (2.0), EED (1.9), ENTPD2 (1.8), FBXO46 (1.3), HMGN2 (1.7), MAD1L1 (1.9), MSL1 (1.4), NDUFAF3 (1.7), NGLY1 (1.5), NLGN2 (2.5), PAAF1 (1.6), PABPN1 (1.7), PRR3 (1.7), RNF167 (1.4), RPA1 (1.3), SHROOM1 (1.8), SLX4 (1.5), SPATA33 (2.1), SPOP (1.4), SRCIN1 (3.5), STIP1 (1.7), STK36 (2.5), STRADB (2.0), TADA2A (1.7), TAF15 (1.7), TERF2 (1.3), U2AF2 (1.3), VPS52 (1.5), VPS72 (1.4), ZNF133 (1.8) |
| hsa-miR-4749-3p  (w/seed GCCCCUC)  (<0.001%) | -3.5 | 40/40 | CFAP298 (1.8), DGKQ (2.0), DNAJB2 (1.8), DOK1 (1.4), ERCC2 (1.3), FASTK (2.1), GFER (1.3), HARS2 (1.6), HMGN2 (1.7), LRRC56 (2.1), LYRM1 (2.0), MANEAL (3.5), MEIS1 (3.2), METRN (1.4), MRPL20-AS1 (1.7), NFIX (1.3), NPDC1 (2.2), NSMCE1 (1.9), PABPN1 (1.7), PACSIN3 (1.6), PDCD7 (1.7), PIAS4 (1.4), PREB (1.3),  PRKACA (1.5), PRPF38A (1.4), PYCR2 (2.0), PYCR3 (1.9), QDPR (1.9), QTRT1 (1.7), RCN3 (4.4), RNF167 (1.4), RRAS (2.1), SLC29A4 (1.9), SRCIN1 (3.5), TMEM216 (1.8), WBP1 (2.7), XPC (1.5), ZFYVE28 (2.9), ZNF502 (1.8), ZNF771 (1.4) |
| hsa-miR-491-3p  (w/seed UUAUGCA)  (<0.001%) | -1.8 | 11/14 | CACYBP (1.7), DMTF1 (2.6), DRG1 (1.4), GINS4 (1.9), HNRNPA2B1 (1.6), MRFAP1L1 (2.3), MTG2 (1.4), RRP8 (1.4), RYK (1.5), ZCCHC9 (1.7), ZNF226 (1.7) |
| hsa-miR-642a-5p  (w/seed UCCCUCU)  (<0.001%) | -2.4 | 10/10 | C12orf65 (2.1), CWF19L2 (1.6), HOXC6 (3.7), KAT2A (2.2), SMCO4 (2.1), SRSF6 (2.1), STRADB (2.0), TTL (1.5), U2AF2 (1.3), ZNF586 (1.5) |
| hsa-miR-105-5p  (w/seed CAAAUGC)  (<0.001%) | 68.9 | 1/12 | SPRR1A (-16.9)  [targets not downregulated - C7orf50, CCDC34, CISD2, COA3, EIF4A2, EXOSC7, GEMIN6, NDUFA2, QDPR, SELL, TRA2B] |
| hsa-miR-1234-3p  (w/seed CGGCCUG)  (<0.001%) | 25.8 | 1/21 | ZBTB7C (-2.0)  [targets not downregulated - ADAMTS13, BTBD2, C12orf65, CERK, DFFA, DGKQ, ERLIN1, H2AX, HDDC3, NDE1, NDOR1, PARK7, POLR2D, PPP1R12B, RANBP10, RRP7A, RUSF1, STX18, TMEM175, TOP3A] |
| hsa-miR-1843  miR-1843a-5p  (w/seed AUGGAGG)  (<0.001%) | 2.5 | 1/27 | PI4K2B (-2.2)  [targets not downregulated – ABR, CALM1, CAMK2N2, CELF1, CENPM, CUEDC2, EXOSC2, H2AX, HARS2, HDDC3, HS3ST1, KCTD6, LINC01006, LZTR1, MPV17, NFIX, RAD23A, RNF187, S100PBP, SNRPG, TCEAL8, THAP3, TMEM107, TSPAN13, TTI1, ZNF589] |
| hsa-miR-30d-5p  miR-30c-5p (w/seed GUAAACA)  (>0.087%) | 1.3 | 5/30 | ADGRA3 (-1.7), NUS1 (-1.5), PI4K2B (-2.2), ZCCHC4 (-1.5), ZDHHC20 (-1.7)  [targets not downregulated - ANKHD1, ANKHD1-EIF4EBP3, ARL4A, C6orf141, C8orf76, CAMK2N2, CCDC14, CCDC18, CISD2, CPOX, CRTAP, EED, ERLIN1, IFRD1, MIS12, MLLT1, NIF3L1, NRBP1, PAAF1, PCGF3, PDE6D, RGPD4, S100PBP, SCYL3, SRSF7, SYCE2] |
| hsa-miR-3179  w/seed GAAGGGG)  (<0.001%) | 2.7 | 3/45 | ABCD1 (-1.9), IGFL1 (-8.3), ZFHX3 (-1.5)  [targets not downregulated - CAMK2N2, CENPM, COA3, CUEDC2, DHFR, DNAL4, DPM2, EFNB1, EPOR, INTS3, KNSTRN, MANEAL, MFSD10, NRBP1, PABPN1, PFDN6, POLR2D, PPP1R12B, PRKACA, RABL2B, RANBP10, RNASEH2C, RNF187, RWDD2B, SAMD1, SNX17, SPATA33, ST3GAL2, STIP1, TAOK2, TCEANC2, TMCO6, TMEM216, TOMM6, TSPAN5, TULP3, U2AF2, WDR27, WDTC1, WNT5B, ZNF691, ZNF875] |
| hsa-miR-320c  miR-320b (w/seed AAAGCUG)  (>0.001%) | 2.1 | 1/14 | ZFHX3 (-1.5)  [targets not downregulated - ARL4A, BANP, CISD2, DSCC1, GTF2H5, KLHDC9, NCAPD3, NSMCE1, PYGO2, RNF187, TCEAL8, TIMM8B, ZNF23] |
| hsa-miR-5010-5p  miR-4525 (w/seed GGGGGAU)  (<0.001%) | 7.7 | 1/50 | CARD11 (-22.2)  [targets not downregulated - BRCA1, C11orf49, C7orf50, CALM1, CCDC61, CELF1, CNOT3, DBNL, DNAL4, DOK1, DOK7, EFNB1, ENTPD2, EPS15L1, FAM193A, FGFRL1, GLI4, HEXIM1, INTS3, KANK2, LIN37, MRPL20-AS1, MTHFSD, MUS81, NFIX, NLGN2, ORAI3, POLR2D, PRKACA, PRPF38B, RAD23A, RRAS, RRP7A, RUSF1, SAMD1, SHROOM1, SLC29A4, SNX17, SPCS2, SPOP, SRCIN1, ST3GAL2, TCEANC2, U2AF2, UBBP4, UQCC1, VPS52, VTN, ZFYVE28, ZNF226] |
| hsa-miR-505-3p  (w/seed GUCAACA)  (>0.001%) | 1.5 | 1/13 | FGF18 (-2.6)  [targets not downregulated – DHFR, DNAJA3, ELP6, ENTPD6, MAEA, MEIS1, POLR3K, TMEM203, UBFD1, UCP2, UPF3B, ZNF133] |
| hsa-miR-549a-3p  (w/seed GACAACU)  (<0.001%) | 1.8 | 1/14 | ADGRA3 (-1.7)  [targets not downregulated - ANKRD26, C12orf65, CCDC137, DMTF1, EED, FAM220A, FRA10AC1, RBM4B, SELENOW, TCEANC2, TEFM, TRA2A, ZNF404] |
| hsa-miR-590-5p  miR-21-5p (w/seed AGCUUAU)  (<0.001%) | 4.3 | 1/14 | FGF18 (-2.6)  [targets not downregulated - CCDC142, ECI2, HAX1, MED28, MTHFSD, NCAPD3, PHF14, PSRC1, SRSF3, TADA2A, TOMM6, VRK3, ZNF589] |
| hsa-miR-597-3p  (w/seed GGUUCUC)  (<0.001%) | 3.4 | 1/28 | APOL1 (-7.0)  [targets not downregulated - ATG10, CCDC18, DPF2, EPOR, HMGN2, HS3ST1, MAEA, MIOS, MKS1, MRM2, MRPL17, NGLY1, NIT1, NPDC1, NUP62, PMS2, RNASEH2A, RWDD2B, SPATA33, SPCS2, TMEM107, TMEM203, ZNF12, ZNF337, ZNF502, ZNF768, ZNF875] |
| hsa-miR-628-3p  (w/seed CUAGUAA)  (>0.001%) | 1.3 | 2/8 | ZCCHC4 (-1.5)  [targets not downregulated - C5orf34, GINS4, HNRNPH3, SUPT7L, TMEM99, XPC] |
| hsa-miR-1287-5p  (w/seed GCUGGAU)  (>0.001%) | 1.4 | 0/20 | [targets not down regulated – ABR, CCDC137, CCDC142, CXXC1, ENTPD2, H2AX, LSM6, MAF1, MAMDC4, MTERF3, PRPF38A, PRR3, PSIP1, PSRC1, RIC8B, SLC27A3, SLC29A4, TBC1D3, THAP11, TLR4] |
| hsa-miR-296-3p  (w/seed AGGGUUG)  (>0.001%) | 1.7 | 0/18 | [targets not downregulated - ANKHD1, ANKHD1-EIF4EBP3, ANKRD54, APBB3, EPOR, FKBP15, HMGN2, HS3ST1, IP6K2, MSANTD2, MTFR1L, PFDN6, SELENOH, TAF15, TERF2, TRMT112, ZNF226, ZNF23 ZNF875] |
| hsa-miR-301a-5p  (w/seed CUCUGAC)  (>0.001%) | 1.6 | 0/6 | [targets not downregulated - ELP5, HMGN2, NFU1, TMEM107, VPS52, ZNF23] |
| hsa-miR-3064-3p  (w/seed UGCCACA)  (<0.001%) | 3.9 | 0/26 | [targets not downregulated - AASDH, CENPJ, CTC1, FAM200B, FANCC, JAGN1, MEIS1, MRFAP1L1, MSL1, NAP1L4, NAPB, NEURL4, NSD2, NUP62, PDCD7, PFDN6, PWWP3A, RPUSD3, RYK, SAFB, SELENOW, TIMM17B, TMEM161A, ZNF226, ZNF23, ZNF623] |
| hsa-miR-373-3p  (w/seed AAGUGCU)  (>0.001%) | 1.5 | 0/26 | [targets not downregulated - ANKRD54, ARL4A, CCDC18, CCS, HIF1AN, MED28, MRPL17, MRPL24, MSL1, ODF2, PHF14, POLQ, PRPF38A, SHROOM1, SPOP, SWI5, TAOK2, TAPT1, TFAP4, TMEM107, TMEM223, ZNF226, ZNF250, ZNF589, ZNF830, ZSCAN9] |
| hsa-miR-3939  (w/seed ACGCGCA)  (<0.001%) | 17.4 | 0/3 | [targets not downregulated - CENPM, CISD2, FIZ1] |
| hsa-miR-421  (w/seed UCAACAG)  (>0.001%) | 1.3 | 0/18 | [targets not downregulated - CCDC14, DNAJA3, EED, ERLIN1, GEMIN6, IP6K2, MPV17, POLR2D, SAMD1, SPCS2, SRSF7, TAF15, TBC1D3, UTP3, ZNF337, ZNF623, ZNF638] |
| hsa-miR-4474-3p  (w/seed UGUGGCU)  (<0.001%) | 2.9 | 0/26 | [targets not downregulated - ANKRD54, ATRIP, CPOX, DALRD3, DMTF1, DOK1, DPF2, ERLIN1, FAM72A, FBXO46, LZTR1, MIB2, MRPL20-AS1, NUDCD3, NUP62, PPP1R12B, PXK, RNF187, SHARPIN, TCEANC2, TMEM138, TMEM216, ZC3H18, ZNF226, ZNF23, ZSCAN21] |
| hsa-miR-4724-5p  (w/seed ACUGAAC)  (<0.001%) | 9.4 | 0/13 | [targets not downregulated - APBB3, CALM1, CWF19L2, DTYMK, MAEA, MANEAL, PHF14, RAB28, RWDD3, SELENOH, SLF1, UBBP4, ZCCHC8] |
| hsa-miR-769-3p  w/seed UGGGAUC)  (>0.001%) | 1.3 | 0/18 | [targets not downregulated - COPS6, CUEDC2, CZIB, DOK1, EHMT2, ELP5, GAMT, GINS4, INTS3, KRI1, LARS2, NDOR1, PPP1R12B, RPA1, TMEM107, UCP2, ZNF250, ZNF875] |
| hsa-miR-874-3p  (w/seed UGCCCUG)  (>0.001%) | 1.5 | 0/25 | [targets not downregulated - CCDC142, CCS, CDAN1, DFFA, DNAJB2, FAM104B, FAM189B, GFER, HIF1AN, IL10RB, KANK2, MRPL20-AS1, PAAF1, PARK7, PIGV, RPAP1, RUSF1, SLC25A11, TIMM17B, TMEM107, TMEM184A, UPF3B, XRCC3, ZFP41, ZNF623] |

All miRs listed have expression changes with significant p values (<0.05)

(% total small reads mapped to hg38) The average %=0.087%

[targets not downregulated]

1. All Tumors vs All Cell lines

| hsa-miR* | Fold change | targets changed/targets in dataset | targets |
| --- | --- | --- | --- |
| **hsa-miR-148b-3p**  miR-148a-3p (w/seed CAGUGCA)  (>0.087%) | 5.1 | 47/50 | ABL2 (-2.0), ADAM10 (-1.7), C16orf70 (-2.3), CD274 (-5.6), CDC25B (-4.1), CDKN1A (-9.1), CEBPG (-2.5), CHD1 (-1.9), CLOCK (-1.9), COL4A1 (-8.8), CYTH3 (-8.2), DNMT1 (-2.0), DNMT3B (-3.6), EGFR (-2.4), EPAS1 (-5.6), FAM161A (-2.3), FOXK2 (-1.4), IGF2BP3 (-33.0), ITGA5 (-2.2), KLHL5 (-9.8), KRTAP2-3/KRTAP2-4 (-117.2), LIPG (-6.9), MED12L (-3.5), MOSPD1 (-2.2), MTF1 (-1.4), MYBL1 (-3.1), NAA15 (-2.2), NPC1 (-2.8), NRP1 (-4.3), OTUD4 (-1.8), PIDD1 (-4.4), PKIB (-5.1), PRRG1 (-5.1), PSMC3IP (-2.4), PTPN14 (-2.2), RHOF (-3.7), RNF44 (-2.3), SESN2 (-4.0) SH2B3 (-4.1), SLC31A2 (-6.2), SMIM13 (-2.3), TAF1D (-2.7), TEAD1 (-2.2), TUBE1 (-2.3), UBA6 (-1.7), WNT10B (-6.2), ZNF488 (-12.7) |
| **hsa-miR-26b-5p**  miR-26a-5p (w/seed UCAAGUA)  (>0.087%) | 6.7 | 32/36 | ABL2 (-2.0), BAK1 (-1.8), C16orf70 (-2.3), CCN2 (-8.3), CDC6 (-3.7), CEP76 (-2.4), CHAC1 (-34.2), CHD1 (-1.9), CKS2 (-3.4), DEPDC1 (-3.3), EZH2 (-3.4), GAN (-2.9), GRHL3 (-2.6), IPO7 (-2.2), JAG1 (-3.1), KIF18A (-2.6), KPNA2 (-2.2), KPNA6 (-1.9), NAA15 (-2.2), NID1 (-15.5), NUP153 (-1.8), OTUD4 (-1.8), PDHX (-1.8), PHLDB2 (-33.8), PMAIP1 (-14.0), POLH (-2.8), POLR3G (-13.9), RNGTT (-1.8), SACS (-3.3), SEPTIN10 (-2.8), VANGL2 (-12.6), ZNF492 (-2.7) |
| **hsa-miR-30d-5p**  miR-30c-5p (w/seed GUAAACA)  (>0.087%) | 3.5 | 41/46 | CARS1 (-3.3), CCN2 (-8.3), CEP76 (-2.4), CHD1 (-1.9), CHI3L1 (-83.4), COL4A1 (-8.8), DBF4 (-3.1), DCUN1D3 (-3.4), FAM72B (-7.1), FAM72C/FAM72D (-8.6), FBXO45 (-1.8), GAN (-2.9), GTF2E2 (-1.7), HOXB8 (-3.4), IER5 (-3.9), IFRD1 (-2.8), ITGA2 (-1.6), MAP3K21 (-3.1), MICB (-7.9), NFAT5 (-2.0), NFATC3 (-2.3), NID1 (-15.5), OTUD4 (-1.8), PAWR (-2.6), PLEKHA8 (-1.8), POLR3G (-13.9), PRDM1 (-10.6), PRRG1 (-5.1), RHEBL1 (-5.0), S100PBP (-2.1), SALL4 (-34.4), SEMA6B (-4.1), SH2B3 (-4.1), SLC38A1 (-2.5), SLC4A7 (-3.6), SNAI1 (-21.9), TEAD1 (-2.2), UAP1 (-1.9), XPO1 (-2.1), YBX1 (-1.8) |
| **hsa-miR-424-5p**  miR-16-5p (w/ seed AGCAGCA)  (>0.001%) | 1412.0 | 19/19 | CCNF (-3.0), CDC25A (-2.4), CDK7 (-1.9), CENPJ (-2.1), DIPK1A (-2.3), EGFR (-2.4), HACE1 (-1.7), IFRD1 (-2.8), IGF1R (-1.7), ITGA2 (-1.6), KIF23 (-2.5), MGAT4A (-3.3), NAA15 (-2.2), PHLDB2 (-33.8), PSAT1 (-5.5), RAD51C (-2.4), RECK (-2.6), SLC38A1 (-2.5), UBE2S (-3.6) |
| hsa-miR-1287-5p  (w/seed GCUGGAU)  (>0.001%) | 182.6 | 27/27 | ALPG (-160.5), APOBEC3H (-12.9), CEP57L1 (-2.3), CLOCK (-1.9), DDB2 (-4.7), DDIAS (-2.7), EGFR (-2.4), GXYLT1 (-2.1), KIAA0040 (-4.0), LIMS3/LIMS4 (-3.1), MAP3K21 (-3.1), MPRIP (-2.2), MUC13 (-6.2), NUP153 (-1.8), PLEKHA8 (-1.8), PRIM2 (-2.0), PRSS35 (-44.1), PSIP1 (-2.1), PSRC1 (-2.0), RGS16 (-10.7), SHC1 (-2.9), SULT1C4 (-277.0), TEAD3 (-2.2), TLR4 (-3.2), YBX1 (-1.8), YBX3 (-4.3) |
| hsa-miR-1307-5p  (w/seed CGACCGG)  (>0.001%) | 4.6 | 1/1 | UHRF1BP1 (-2.6) |
| hsa-miR-3074-5p  (w/seed UUCCUGC)  (>0.001%) | 4.1 | 8/8 | DDIAS (-2.7), EML2 (-3.1), IGFL2 (-17.7), NEK2 (-2.5), PIMREG (-2.3), PMAIP1 (-14.0), RHOV (-6.6), SLC25A19 (-2.3) |
| hsa-miR-324-5p  (w/seed GCAUCCC)  (>0.001%) | 4.2 | 26/28 | ABL2 (-2.0), ANKRD27 (-1.8), BUB1 (-2.8), CD274 (-5.6), DENND1A (-2.0), FOXO1 (-2.0), GAN (-2.9), HES2 (-16.7), IDH3A (-2.1), KIAA0040 (-4.0), KIF20A (-3.1), LTBP2 (-2.0), MAD2L2 (-2.6), MICALL1 (-3.2), NUP43 (-1.7), OPRL1 (-2.8), OTUD3 (-2.7), PRKAB2 (-2.4), PVR (-2.8), RNF44 (-2.3), SGO1 (-2.4), SMIM13 (-2.3), SPRR2D (-25.5), TBC1D2 (-4.4), UHRF1BP1 (-2.6), ZNF724 (-2.7) |
| hsa-miR-371b-3p  miR-290b-3p ( w/seed AGUGCCC)  (>0.087%) | -18.2 | 4/33 | GALNT10 (4.7), PPP1R1B (344.0), PRRG2 (2.5), ZSWIM5 (11.7) |
| hsa-let-7f-1-3p  let-7a-3p (w/seed UAUACAA)  (>0.001%) | -2.2 | 1/34 | PLPP1 (1.9) |
| hsa-miR-125a-3p  (w/seed CAGGUGA)  (>0.001%) | -2.1 | 4/39 | DNAJC22 (24.0), KIF13B (2.6), KLK6 (6.9), LGALS3BP (12.4) |
| hsa-miR-1296-5p  (w/seed UAGGGCC)  (>0.001%) | -3.0 | 4/21 | GALNT10 (4.7), HID1 (3.0), LGALS3BP (12.4), TRAPPC6A (2.1) |
| hsa-miR-1306-5p  (w/seed CACCUCC)  (>0.001%) | -2.1 | 3/28 | OS9 (3.2), ZNF710 (2.0), ZNF768 (1.9) |
| hsa-miR-149-5p  (w/seed CUGGCUC)  (>0.001%) | -3.8 | 2/32 | DNAJC22 (24.0), GALNT6 (2.5) |
| hsa-miR-185-5p  (w/seed GGAGAGA)  (>0.001%) | -1.6 | 5/49 | FOXP4 (2.0), NFIX (2.3), RAB40B (3.8), TNFSF13 (7.5), ZBTB7C (6.6) |
| hsa-miR-18a-3p  (w/seed CUGCCCU)  (>0.001%) | -2.7 | 5/34 | DNAJC4 (2.0), OS9 (3.2), PPP1R13B (3.4), PRRG2 (2.5), ZNF768 (1.9) |
| hsa-miR-191-3p  (w/seed CUGCGCU)  (>0.001%) | -3.3 | 1/5 | TLE5 (1.6) |
| hsa-miR-23a-5p  (w/seed GGGUUCC)  (>0.001%) | -4.4 | 3/26 | OS9 (3.2), PLCH1 (3.3), PSD4 (5.4) |
| hsa-miR-296-3p  (w/seed AGGGUUG)  (>0.001%) | -2.8 | 1/10 | KLK6 (6.9) |
| hsa-miR-296-5p  (w/seed GGGCCCC)  (>0.001%) | -2.4 | 10/71 | FOXP4 (2.0), GALNT10 (4.7), GALNT6 (2.5), HIPK2 (3.5), KIF13B (2.6), NFIX (2.3), OS9 (3.2), TNFSF13 (7.5), VGLL4 (2.8), ZNF768 (1.9) |
| hsa-miR-302b-3p  miR-291a-3p (w/seed AAGUGCU)  (>0.001%) | -228.6 | 3/39 | NFIB (6.7), YPEL2 (5.1), ZBTB18 (2.1) |
| hsa-miR-372-5p  miR-295-5p (w/seed CUCAAAU)  (>0.001%) | -15.9 | 1/9 | RAB40B (3.8) |

All miRs listed have expression changes with significant p values (<0.05)

(% total small reads mapped to hg38) The average %=0.087%
