# Supplementary material for: Major Role for Cellular MicroRNAs, Long Noncoding RNAs (lncRNAs), and the Epstein-Barr Virus-Encoded BART lncRNA during Tumor Growth In Vivo
Source: mBio. 2022 Apr 18;13(3):e00655-22. doi: 10.1128/mbio.00655-22 (PMC9239068; doi:10.1128/mbio.00655-22)
Supplement: TABLE S4 [file mbio.00655-22-s0005.docx]

Table S4. Differentially expressed lncs

A. Differentially expressed lncRNAs between tumors and corresponding cell line (p value and FDR <0.05)

|  | lncRNAs upregulated* | lncRNAs down regulated** |
| --- | --- | --- |
| AE T vs AE CL  193 T only  58 up in T  57 down in T  104 CL only | **AC004076.2, AC004466.3, AC004803.1, AC004918.1, AC005229.4, AC005332.4, AC005534.1, AC006008.1, AC006027.1, AC006042.1, AC006111.2, AC006230.1, AC007405.3, AC007608.1, AC007608.2, AC007663.4, AC008105.1, AC008264.2, AC008537.4, AC008555.4, AC008610.1, AC008915.3, AC009093.6, AC009107.2, AC009120.2, AC009120.3, AC010168.2, AC010186.3, AC010201.2, AC010319.4, AC010719.1, AC010733.1, AC010969.2, AC011462.4, AC011472.3, AC011477.1, AC012073.1, AC012531.1, AC016026.1, AC016773.2, AC017100.1, AC021321.1, AC022007.1, AC023494.1, AC023509.3, AC024060.2, AC024267.7, AC024560.2, AC024560.4, AC025165.5, AC026336.3, AC026368.1, AC026740.1, AC026748.3, AC034236.2, AC046143.1, AC060780.1, AC064836.3, AC068205.2, AC068790.3, AC068790.7, AC069120.1, AC073195.2, AC073611.1, AC073896.2, AC073896.3, AC078846.1, AC083843.3, AC084018.2, AC084198.4,, AC087292.2, AC090116.1, AC091132.1, AC091729.3, AC092119.2, AC092171.2, AC092171.4, AC092375.2, AC093827.4, AC095057.3, AC099778.1, AC102953.2, AC103691.1, AC104532.2, AC107375.1, AC108673.3, AC114490.1, AC116158.1, AC116158.3, AC118344.1, AC119403.1, AC125257.1, AC125494.1, AC126755.3, AC127024.2, AC127024.5, AC136443.3, AC138393.3, AC139795.2, AC142472.1, AC144548.1, AC145207.5, AC145207.7, AC232271.1, AC234917.3, AC243919.2, AC245297.4, AC245884.1, AC245884.8, AC245884.9, ADNP-AS1, AL021707.6, AL022322.1, AL031985.3, AL035461.3, AL109615.4, AL118505.1, AL121829.2, AL122058.1, AL133410.1, AL136221.1, AL136304.1, AL137058.2, AL139089.1, AL158214.2, AL162458.1, AL354707.1, AL354740.1, AL355488.1, AL357079.1, AL390066.2, AL445222.2, AL513548.1, AL590064.1, AP000786.1, AP000866.6, AP001160.3, AP001267.1, AP001453.2, AP002840.2, AP003419.3, AP006222.2, AUXG01000058.1, C1RL-AS1, C2CD4D-AS1, C8orf44, CAPN10-DT, CERS6-AS1, CYMP-AS1, DBH-AS1, DNAAF4-CCPG1, ERICD, FAM13A-AS1, FEZF1-AS1, FTX, GARS1-DT, GPRC5D-AS1, HCG18, HMGA2-AS1, HOXC-AS1, LINC00115, LINC00174, LINC00339, LINC00648, LINC00680, LINC00842, LINC00869, LINC00909, LINC01004, LINC01089, LINC01138, LINC01355, LINC01711, LINC02820, MIR193BHG, MIR205HG, MIR600HG, N4BP2L2-IT2, PDCD4-AS1, PRR7-AS1, PSMG3-AS1, RNASEH1-AS1, RNF139-AS1, SH3BP5-AS1, SLC16A1-AS1, STARD7-AS1, TAPT1-AS1, TFAP2A-AS1, TMEM44-AS1, TSPOAP1-AS1, Z83843.1, ZFHX2-AS1, ZNF436-AS1**  AC005041.5, AC005083.1, AC005261.3, AC009283.1, AC010327.6, AC010542.6, AC011498.6, AC012467.2, AC020907.4, AC020915.2, AC020915.3, AC020978.5, AC026362.1, AC027307.2, AC037459.2, AC048341.2, AC073957.3, AC074117.1, AC084125.2, AC093525.6, AC109322.1, AC132872.1, AC245060.7, AF001548.2, AL109627.1, AL132655.2, AL356740.3, AL390719.2, AP000525.1, AP003900.1, ASMTL-AS1, BLACAT1, CU634019.6, FBXL19-AS1, HOXC-AS2, JPX, LINC00623, LINC00665, LINC00888, LINC01145, LINC02381, MAFG-DT, MINCR, MIR200CHG, MMP25-AS1, MRPL20-AS1, NRAV, PPP1R26-AS1, PSMA3-AS1, PTOV1-AS2, RAB11B-AS1, SLC9A3-AS1, STAG3L5P-PVRIG2P-PILRB, TMEM147-AS1, U47924.2, VASH1-AS1, XIST, ZKSCAN2-DT | **AC004471.1, AC004585.1, AC005540.1, AC006262.2, AC007952.1, AC007952.4, AC008119.1, AC008147.2, AC008443.5, AC008687.2, AC008687.3, AC008753.3, AC008982.2, AC011462.5, AC012645.4, AC016588.2, AC018410.1, AC018695.4, AC022613.3, AC023946.1, AC026202.3, AC039056.2, AC040169.3, AC048344.4, AC055811.1, AC073569.2, AC087533.1, AC087752.3, AC092053.4, AC093866.1, AC097382.3, AC104024.2, AC105036.3, AC105206.2, AC129926.1, AC138956.2, AC140479.4, AC243960.3, AL021878.2, AL022328.3, AL031118.1, AL031670.1, AL035587.2, AL109955.1, AL121906.2, AL133346.1, AL137800.1, AL138689.1, AL138789.1, AL139095.5, AL139407.1, AL157394.3, AL158196.1, AL161431.1, AL162231.2, AL357033.2, AL360012.1, AL391425.1, AL592166.1, AL606500.1, AL645608.7, AL645933.4, AL662890.1, AL772307.1, ALMS1-IT1, AP000547.3, AP000593.3, AP001033.4, AP003559.1, AP005212.4, ARAP1-AS2, C6orf99, CASC19, CASC8, CRIM1-DT, CYTOR, EML2-AS1, FLG-AS1, GAS6-AS1, HDAC4-AS1, IDH1-AS1, IDI2-AS1, IGFL2-AS1, LINC00973, LINC01300, LINC01356, LINC01480, LINC01564, LINC01775, LINC01881, LINC02086, LINC02178, LINC02273, LINC02560, LINC02846, MIR302CHG, NALT1, PCAT19, POLH-AS1, PURPL, RAD21-AS1, SOS1-IT1, UBR5-AS1, ZNF687-AS1**  AC004264.1, AC004943.2, AC005256.1, AC009118.2, AC010624.5, AC010761.1, AC012181.1, AC012181.2, AC021078.1, AC022211.2, AC022211.4, AC023157.2, AC025423.4, AC026401.3, AC027644.3, AC064836.2, AC069148.1, AC092803.1, AC112907.3, AC122129.1, AC137932.3, AC239868.1, AC245140.2, ADIRF-AS1, AL031714.1, AL118516.1, AL133243.2, AL359513.1, AL445524.1, AL590004.3, AL590705.1, AL606834.1, AP000692.1, AP001453.4, AP001816.1, ARHGAP5-AS1, ATP2A1-AS1, BCYRN1, BTG3-AS1, BX842570.1, DBF4B, FLNB-AS1, FOXD2-AS1, LINC00659, LINC01003, LINC01843, LINC02802, MELTF-AS1  PVT1, RNF213-AS1, SMARCA5-AS1, SNHG1, SOCS2-AS1, TMEM202-AS1, UCA1, Z98884.2, ZEB1-AS1 |
| BART T vs BART CL  19 T only  2 up in T  46 down in T  86 CL only | **AC007128.2, AC010168.2, AC025154.2, AC080037.2, AC130456.2, AC244517.4, AC244517.6, ADNP-AS1, AL353759.1, AL450345.2, DDN-AS1, ELFN1-AS1, FEZF1-AS1, LINC01876, LINC02672, NKILA, PAXIP1-AS2, TAPBPL, Z97192.1**  LINC01145 ZNF503-AS2 | **AC004585.1, AC005330.1, AC005540.1, AC007952.1, AC008622.2, AC008753.3, AC010894.2, AC012181.2, AC012464.1, AC012640.4, AC022098.1, AC022211.2, AC023157.2, AC040970.1, AC079921.2, AC087752.3, AC093484.4, AC093866.1, AC103957.2, AC104024.2, AC104024.3, AC104667.2, AC112907.3, AC122129.1, AC129926.1, AC133644.2, AC244153.1, AL121759.2, AL133346.1, AL138689.1, AL139352.1, AL161431.1, AL353138.1, AL354872.1, AL391425.1, AL645608.7, AL645608.8, ALMS1-IT1, AP000547.3, AP000851.1, AP002478.1, AP003559.1, BX539320.1, C8orf31, CASC19, CDK6-AS1, CEBPB-AS1, CRIM1-DT, DLEU2, DLGAP1-AS2, EMC3-AS1, FAM225A, FAM225B, FLG-AS1, HECW2-AS1, KTN1-AS1, LIF-AS1, LINC00319, LINC00842, LINC00973, LINC01224, LINC01260, LINC01300, LINC01356, LINC01411, LINC01480, LINC01979, LINC02273, LINC02387, LINC02732, LINC02783, LINC02802, LINC02846, MIR1915HG, MIR302CHG, NALT1, ODC1-DT, PINCR, PURPL, PVT1, SLC16A1-AS1, SNHG26, SOCS2-AS1, TRIM31-AS1, U62317.1, UBR5-AS1**  AC005523.2, AC008753.2, AC009093.4, AC010761.1, AC022137.4, AC025423.4, AC074117.1, AC092803.1, AC108488.3, AC125807.2, AC145124.1, AC245041.2, AL365181.3, AL590004.3, AL590666.2, AP001816.1, ARHGAP5-AS1, ATP2A1-AS1, BCYRN1, C6orf99, CCDC84-DT, DBF4B, EML2-AS1, FAM198B-AS1, GAS5, HCG15, LINC00659, LINC01133, LINC01843, LINC02331, LINC02747, MNX1-AS1, NOP14-AS1, NUP50-DT, PCCA-DT, RNASEH1-AS1, SNHG1, SNHG12, SNHG15, SNHG17, SNHG3, SNHG32, TMEM202-AS1, TPM3P9, UCA1, VPS9D1-AS1 |
| pc T vs pc CL  6 T only  2 up in T  57 down in T  122 CL only | **AC007128.2, AC080037.2, AC116351.2, AC245297.4, ELFN1-AS1, TAPBPL**  AL391121.1, SCARNA9 | **AC002094.1, AC004585.1, AC004943.2, AC005329.1, AC005330.1, AC007342.5, AC007906.2, AC008753.3, AC008870.3, AC008982.2, AC009065.9, AC012360.3, AC012464.1, AC015917.2, AC016588.2, AC018410.1, AC022007.1, AC022098.1, AC022211.2, AC022211.4, AC069120.1, AC079354.1, AC079466.1, AC079684.2, AC079921.2, AC083843.3, AC087392.5, AC087752.3, AC090587.1, AC092803.1, AC093866.1, AC102953.2, AC103957.2, AC104024.2, AC104024.3, AC105219.1, AC108488.3, AC112907.3, AC122129.1, AC127526.4, AC129926.1, AC133644.2, AC145124.1, AC242988.2, AC247036.1, AL031670.1, AL049795.2, AL121658.1, AL121759.2, AL133346.1, AL133477.1, AL133499.1, AL137129.1, AL138689.1, AL139289.1, AL139352.1, AL161891.1, AL355075.6, AL356299.3, AL391425.1, AL445524.1, AL606500.1, AL645608.2, AL645608.7, AL928970.1, AP000851.1, AP001453.4, AP002478.1, AP005482.4, APCDD1L-DT, ATP2A1-AS1, BX539320.1, C1QTNF1-AS1, C6orf99, C8orf31, CASC19, CCDC84-DT, CDK6-AS1, COX10-AS1, CRIM1-DT, EMC3-AS1, FAM198B-AS1, FAM225A, FAM225B, FLG-AS1, GASAL1, GCC2-AS1, HECW2-AS1, IPO9-AS1, LINC00313, LINC00319, LINC00337, LINC00842, LINC00973, LINC01224, LINC01260, LINC01300, LINC01356, LINC01411, LINC01480, LINC01843, LINC02273, LINC02331, LINC02365, LINC02802, LINC02846, MAST4-AS1, MGAT3-AS1, MIR1915HG, MIR302CHG, MIR600HG, MNX1-AS1, NALT1, PINCR, PURPL, PVT1, SCARNA13, SLC16A1-AS1, SNHG26, SOCS2-AS1, TNFRSF10A-AS1, TRIM31-AS1**  AC005523.2, AC005540.1, AC006213.2, AC008753.2, AC010327.6, AC010761.1, AC022137.4, AC025423.4, AC026401.3, AC074117.1, AC099329.1, AC125611.3, AC125807.2, AC138028.4, AC245041.1, AC245041.2, AC245060.7, AL139287.1, AL158206.1, AL161431.1, AL365181.3, AL590004.3, AL590666.2, AL606834.1, AL662791.1, AP000525.1, AP000786.1, AP001505.1, AP003392.1, ARHGAP5-AS1, BCYRN1, DBF4B, DGCR11, EML2-AS1, EMSLR, HCG15, HOXB-AS3, IGFL2-AS1, LIMD1-AS1, LINC00857, LINC01133, LINC02313, LINC02747, MAFG-DT, MMP25-AS1, NOP14-AS1, PCCA-DT, RNASEH1-AS1, SMARCA5-AS1, SNHG1, SNHG12, SNHG15, SNHG17, SNHG3, TPM3P9, UCA1, VPS9D1-AS1 |
| All Ts vs CLs  (run all Ts vs all Cell lines)  Likely pulls out more as it has 3 cell lines to compare to for stronger statistics  43 T only  14 up in T  43 down in T  73 CL only | **AC005332.4, AC007996.1, AC008555.4, AC010168.2, AC011477.1, AC020656.2, AC021016.2, AC025154.2, AC025181.2, AC060780.1, AC068338.2, AC087591.1, AC090114.2, AC112220.2, AC245297.4, AL078587.2, AL121820.2, AL356740.1, AP001160.3, CASC9, COLCA1, FEZF1-AS1, FUT8-AS1, GPRC5D-AS1, IL10RB-DT, KCNQ1OT1, LINC00324, LINC01291, LINC01978, LINC01996, MIR210HG, PAXIP1-AS2, PDCD4-AS1, RHPN1-AS1, SCAMP1-AS1, SLC25A25-AS1, STARD7-AS1, TAPBPL, TONSL-AS1, TPM1-AS, TSPEAR-AS1, USP27X-AS1, ZNF503-AS2AC136475.3**  AL391121.1, AL499602.1, AL591895.1, BAIAP2-DT, FAM111A-DT, FGD5-AS1, HCG11, KDM7A-DT, MCF2L-AS1, PCAT7, PINK1-AS, PSMA3-AS1, RAB11B-AS1, SCARNA9 | **AC004263.2, AC004585.1, AC005540.1, AC007906.2, AC008622.2, AC008753.3, AC009065.9, AC009118.2, AC016588.2, AC022098.1, AC022211.2, AC022211.4, AC023157.2, AC040970.1, AC087752.3, AC090587.2, AC092803.1, AC093866.1, AC097382.3, AC104024.2, AC112907.3, AC122129.1, AC129926.1, AC133644.2,**  **AC140479.4, AL121759.2, AL133346.1, AL133477.1, AL138689.1, AL138789.1, AL139289.1, AL161431.1, AL355075.6, AL391425.1, AL445524.1, AL645608.7, AL645608.8, AL928970.1, AP001453.4, ATP2A1-AS1, ATP6V1G2-DDX39B, C6orf99, CASC19, CRIM1-DT, CYTOR, EML2-AS1, FAM198B-AS1, FAM225A, FAM225B, FLG-AS1, GAS6-AS1, IGFL2-AS1, LINC00973, LINC01133, LINC01224, LINC01300, LINC01356, LINC01411, LINC01843, LINC01881, LINC02273, LINC02802, LINC02846, MIR1915HG, MIR302CHG, MIR4435-2HG, MNX1-AS1, NALT1, PINCR, PURPL, SOCS2-AS1, ZNF687-AS1**  ABALON, AC005523.2, AC008753.2, AC010761.1, AC015912.3, AC022137.4, AC025423.4, AC092139.1, AC093673.1, AC099329.1, AC108449.2, ADIRF-AS1, AL160006.1, AL365181.3, AL590004.3, AL590666.2, AL606834.1, AP001816.1, AP001931.2, ARHGAP5-AS1, BCYRN1, DBF4B, EMSLR, FLNB-AS1, FOXD2-AS1, GAS5, HOXB-AS3, LINC01315, LINC01709, LINC02747, NOP14-AS1, PCCA-DT, PVT1, SNHG1, SNHG12, SNHG15, SNHG16, SNHG17, SNHG3, SNHG32, THAP9-AS1, TMEM202-AS1, TPM3P9, UCA1 |
| All Ts vs CLs  (compare each individual result and looking for commonality |  | **AC004585.1**, **AC008753.3, AC087752.3**, **AC093866.1**, **AC104024.2**, **AC129926.1, AL133346.1**, **AL138689.1**, **AL391425.1, AL645608.7, CASC19**, **CRIM1-DT**, **FLG-AS1**, **LINC00973**, **LINC01300**, **LINC01356**, **LINC01480, LINC02273**, **LINC02846**, **MIR302CHG**, **NALT1**, **PURPL,**  AC005540.1, AC010761.1, AC022211.2, AC025423.4, AC092803.1, AC112907.3, AC122129.1, AL161431.1, AL590004.3 , ARHGAP5-AS1, ATP2A1-AS1, BCYRN1, C6orf99, DBF4B, EML2-AS1, LINC01843, LINC02802, PVT1, SNHG1, SOCS2-AS1, UCA1 |

*Bolded are only expressed in T (<0.5rpkms in cell lines)

** Bolded are only expressed in CL (<0.5rpkms in tumors)

B. Differentially expressed lncRNAs between AGS-EBV and BART cell lines and pc control cell line (p value and FDR <0.05)

|  | lncRNAs upregulated* | lncRNAs down regulated** |
| --- | --- | --- |
| AE CL vs pc CL  75 AE only  47 up AE  34 down AE  53 pc only | **AC002553.1, AC005041.3, AC005261.3, AC006262.2, AC007952.1, AC008119.1, AC008147.2, AC008443.5, AC008687.3, AC011462.5, AC011472.1, AC012181.2, AC012321.1, AC012645.4, AC018809.1, AC020765.2, AC022613.3, AC023946.1, AC024896.1, AC026202.3, AC039056.2, AC040169.3, AC048344.4, AC069148.1, AC073569.2, AC087239.1, AC087533.1, AC092053.4, AC105036.3, AC138956.2, AC243960.3, AC245052.4, AL021878.2, AL022328.3, AL031118.1, AL035587.2, AL109955.1, AL133243.2, AL137800.1, AL139095.5, AL157394.3, AL158196.1, AL162231.2, AL357033.2, AL359513.1, AL360012.1, AL442125.2, AL645933.4, AL662884.3, ALMS1-IT1, AP000547.3, AP000593.3, AP000692.1, AP000894.4, AP001033.4, AP003559.1, AP005212.4, ARAP1-AS2, FOXP4-AS1, HDAC4-AS1, IDI2-AS1, LINC01054, LINC01564, LINC01775, LINC02086, LINC02178, LINC02560, PCAT19, POLH-AS1, RAD21-AS1, RNF213-AS1, SOS1-IT1, TCERG1L-AS1, UBR5-AS1, Z98884.2**  AC004264.1, AC004585.1, AC004943.2, AC005253.1, AC005540.1, AC008735.2, AC010624.5, AC012181.1, AC016588.2, AC018410.1, AC018695.4, AC021078.1, AC023157.2, AC026401.3, AC027644.3, AC055811.1, AC092803.1, AC093866.1, AC129926.1, AC137932.3, AC239868.1, AC245140.2, AL031670.1, AL031714.1, AL118516.1, AL133346.1, AL161431.1, AL391425.1, AL590705.1, AL606834.1, AP001453.4, ARRDC1-AS1, BCYRN1, BTG3-AS1, BX537318.1, BX842570.1, KMT2E-AS1, LINC00659, LINC01480, LINC01843, LINC02273, LINC02846, MELTF-AS1, NALT1, PVT1, SNHG1, ZEB1-AS1 | **AC002094.1, AC009041.3, AC012464.1, AC069120.1, AC079354.1, AC079684.2, AC079921.2, AC090587.1, AC102953.2, AC103957.2, AC105219.1, AC127526.4, AC145124.1, AC234917.3, AC243919.2, AC245041.1, AC245041.2, AC247036.1, AL049795.2, AL133499.1, AL139352.1, AL589986.2, AL645608.2, AP000786.1, AP000851.1, AP000866.6, AP002478.1, AP005242.1, AP005482.4, APCDD1L-DT, C1QTNF1-AS1, C8orf31**  **CCDC84-DT, CDK6-AS1, GASAL1, HCG15, HCG18, HECW2-AS1, KC877982.1, LINC00313, LINC00319, LINC00680, LINC00842, LINC01260, LINC01594, LINC02331, LINC02365, MAST4-AS1, PINCR, RNASEH1-AS1, SLC16A1-AS1, SNHG26, TRIM31-AS1**  AC004816.1, AC007906.2, AC008753.2, AC008753.3, AC022137.4, AC074117.1, AC104024.2, AC108488.3, AC125807.2, AC245060.7, AL121759.2, AL158206.1, AL590004.3, AL662791.1, ATP2A1-AS1, CASC19, DANCR, DGCR11, EMC3-AS1, FLG-AS1, IGFL2-AS1, LINC00665, LINC01133, LINC01300, LINC01356, LINC02747, MAFG-DT, MIR1915HG, MNX1-AS1, PCCA-DT, SMARCA5-AS1, SOCS2-AS1, TPM3P9, VPS9D1-AS1 |
| BART CL vs pc CL  14 BART only  11 up BART  18 down  19 pc only | **AC104667.2, AC244153.1, AL353138.1, AL354872.1, AP000547.3, AP003559.1, DLEU2, KTN1-AS1, LIF-AS1, LINC01979, LINC02783, ODC1-DT, SFTA1P, U62317.1**  AC009093.4, AC023157.2, AC104024.2, AL590666.2, AP000851.1, BCYRN1, LINC01300, LINC02331, MIR1915HG, SNHG32, SOCS2-AS1 | **AC002094.1, AC005329.1, AC018410.1, AC069120.1, AC079466.1, AC079684.2, AC087392.5, AC105219.1, AC127526.4, AC242988.2, AL049795.2, AL589986.2, AL645608.2, APCDD1L-DT, C1QTNF1-AS1, KC877982.1, LINC00313, LINC01594, LINC02365**  AC022211.2, AC022211.4, AC025423.4, AC103957.2, AC125611.3, AC245060.7, AL139287.1, AL391425.1, AP005482.4, CASC19, DANCR, FLG-AS1, IGFL2-AS1, LINC01356, LINC02273, LINC02747, PINCR, PURPL |
| Common AE and BART CLs vs pc CL | **AP000547.3**, **AP003559.1**, AC023157.2, BCYRN1 | **AC002094.1**, **AC069120.1**, **AC079684.2**, **AC105219.1**, **AC127526.4**, **AL049795.2**, **AL589986.2**, **AL645608.2**, **APCDD1L-DT**, **C1QTNF1-AS1**, **KC877982.1**, **LINC00313**, **LINC01594**, **LINC02365,** AC103957.2, AP005482.4, , PINCR, AC245060.7, CASC19, DANCR, FLG-AS1, IGFL2-AS1, LINC01356, LINC02747 |

*Bolded are only expressed in AE and/or BART cell lines (>0.5 rpkms)

** Bolded are only expressed in pc cell line (>0.5 rpkms)

C. Differentially expressed lncRNAs between AGS-EBV and BART tumors and pc control tumors (p value and FDR <0.05)

|  | lncs upregulated* | lncs downregulated** |
| --- | --- | --- |
| AE Ts vs pc T  269 AE only  151 up AE  5 down AE  8 pc only | **AC004076.2, AC004253.1, AC004466.3, AC004943.2, AC005034.4, AC005104.1, AC005261.3, AC005534.1, AC005606.2, AC005838.3, AC006008.1, AC006027.1, AC006042.1, AC006111.2, AC006230.1, AC007009.1, AC007292.1, AC007405.3, AC007608.1, AC007608.2, AC007663.4, AC008105.1, AC008264.2, AC008537.4, AC008610.1, AC008622.2, AC008760.1, AC008764.8, AC009065.9, AC009093.6, AC009107.2, AC009120.2, AC009120.3, AC009133.1, AC009690.2, AC010186.3, AC010201.2, AC010319.4, AC010331.1, AC010733.1, AC011462.4, AC011472.3, AC011498.6, AC012073.1, AC012531.1, AC016026.1, AC016026.2, AC016394.3, AC016747.3, AC016773.2, AC018809.1, AC018904.1, AC020907.4, AC021321.1, AC022007.1, AC022098.1, AC022211.2, AC022400.6, AC023494.1, AC023509.3, AC024267.4, AC024267.7, AC024560.2, AC024560.4, AC025165.5, AC025370.2, AC026333.4, AC026336.3, AC026356.1, AC026471.4, AC034236.2, AC040162.3, AC064836.3, AC067838.1, AC068205.2, AC068473.5, AC068790.3, AC068790.5, AC069120.1, AC069281.2, AC069544.1, AC073195.2, AC073487.1, AC073575.4, AC073611.1, AC073842.2, AC073896.3, AC078846.1, AC078860.3, AC078883.1, AC079414.3, AC083843.3, AC084018.1, AC084125.2, AC084125.4, AC084198.4, AC087292.2, AC090116.1, AC090198.1, AC091132.1, AC091729.3, AC092119.2, AC092171.2, AC092171.4, AC092301.1, AC095057.3, AC098484.4, AC099522.2, AC099778.1, AC102953.2, AC107068.1, AC107375.1, AC108488.3, AC108673.3, AC114490.1, AC116158.1, AC116158.3, AC118344.1, AC119403.1, AC120114.1, AC124319.1, AC125257.1, AC125494.1, AC127024.2, AC131097.2, AC133528.1, AC136443.3, AC138696.2, AC138932.6, AC139100.2, AC142472.1, AC144548.1, AC145207.5, AC145207.7, AC244197.2, AC245052.4, AC245884.1, AC245884.8, AC245884.9, AC253536.6, ADAM1A, ADNP-AS1, AF129075.4, AL008729.2, AL021707.2, AL021707.3, AL022238.3, AL023803.2, AL031123.2, AL031673.1, AL031778.1, AL035071.1, AL096701.4, AL109615.4, AL118505.1, AL121829.2, AL132655.1, AL133243.2, AL133338.1, AL133410.1, AL136221.1, AL136304.1, AL138478.1, AL138885.3, AL139089.1, AL139317.3, AL158214.2, AL161729.4, AL162258.2, AL162458.1, AL354707.1, AL354740.1,, AL355388.1, AL355488.1, AL357079.1, AL358115.1, AL358472.5, AL390066.2, AL390728.6, AL442125.1, AL445222.2, AL451050.2, AL513218.1, AL513327.1, AL513548.1, AL513550.1, AL928970.1, AP000866.6, AP000894.4, AP001267.1, AP001273.1, AP001453.4, AP002490.1, AP002807.1, AP003065.2, AP003419.3, AP005233.2, AP006222.2, AP006623.1, ASMTL-AS1, AUXG01000058.1, BOLA3-AS1, C8orf44, CCDC18-AS1, CERS6-AS1, CHKB-DT, CKMT2-AS1, CYMP-AS1, DBH-AS1, DLGAP1-AS2, DM1-AS, ELOA-AS1, EMC3-AS1, FAM13A-AS1, FAM225A, FAM225B, FIRRE, FOXP4-AS1, FTX, GARS1-DT, HMGA2-AS1, HOXC-AS1, HOXC-AS2, LINC00174, LINC00339, LINC00514, LINC00622, LINC00842, LINC00869, LINC01004, LINC01138, LINC01355, LINC01711, LINC01843, MAP3K14-AS1, MIR181A2HG, MIR193BHG, MIR205HG, MIR600HG, MRPL23-AS1, N4BP2L2-IT2, NBR2, NDUFA6-DT, PCAT14, PRKCZ-AS1, PRR7-AS1, PSMG3-AS1, PVT1, RAB30-DT, RNF139-AS1, RNF213-AS1, SLC16A1-AS1, SNHG26, SOCS2-AS1, SPACA6P-AS, SPAG5-AS1, TAPT1-AS1, TFAP2A-AS1, TMED2-DT, TPT1-AS1, TSPOAP1-AS1, WARS2-AS1, YEATS2-AS1, Z82243.1, Z98884.2, ZEB1-AS1, ZFHX2-AS1, ZNF213-AS1, ZNF436-AS1**  AC004148.1, AC004951.1, AC005041.5, AC005083.1, AC005253.1, AC005261.1, AC006449.7, AC008115.3, AC008735.2, AC008753.2, AC008894.2, AC009283.1, AC010168.2, AC010327.6, AC010542.6, AC010719.1, AC010761.1, AC010973.2, AC011477.1, AC012321.1, AC012467.2, AC015813.1, AC020915.2, AC020915.3, AC020978.5, AC022137.4, AC024060.2, AC026362.1, AC026368.1, AC026401.3, AC027307.2, AC037459.2, AC048341.2, AC060780.1, AC073896.2, AC073957.3, AC074117.1, AC084018.2, AC092375.2, AC092718.4, AC093297.2, AC093525.6, AC093827.4, AC103691.1, AC106782.5, AC109322.1, AC110285.2, AC125611.3, AC126755.3, AC127024.5, AC127502.2, AC132192.2, AC132872.1, AC138028.4, AC145285.6, AC147651.1, AC232271.1, AC239868.1, AC245060.7, AC245297.4, AF001548.2, AL021707.6, AL022322.1, AL109811.2, AL122058.1, AL132655.2, AL133215.2, AL139287.1, AL161669.3, AL354733.3, AL356740.3, AL365181.3, AL390719.2, AL391244.2, AL590064.1, AL590666.2, AL671710.1, AL731571.1, AP000254.2, AP000525.1, AP002360.2, AP003900.1, AP006621.3, ARRDC1-AS1, ASB16-AS1, BCYRN1, BLACAT1, BX470102.1, BX842570.1, C1RL-AS1, CAPN10-DT, CU634019.6, CU638689.5, DBF4B, FBXL19-AS1, INE1, JPX, KMT2E-AS1, LENG8-AS1, LINC00115, LINC00623, LINC00648, LINC00659, LINC00680, LINC00888, LINC00997, LINC01006, LINC01089, LINC01145, LINC01311, LINC02604, LMNTD2-AS1, LOXL1-AS1, MAFG-DT, MALAT1, MELTF-AS1, MINCR, MIR200CHG, MIR34AHG, MIR4453HG, MIRLET7A1HG, MMP25-AS1, MRPL20-AS1, MUC20-OT1, NFYC-AS1, NOP14-AS1, PPP1R26-AS1, PTOV1-AS2, RAB11B-AS1, RAB4B-EGLN2, RNASEH1-AS1, RUSC1-AS1, SH3BP5-AS1, SLC9A3-AS1, SNHG1, SNHG12, SNHG15, SNHG19, SNHG3, STAG3L5P-PVRIG2P-PILRB, THUMPD3-AS1, TMEM147-AS1, TMEM44-AS1, TNRC6C-AS1, TPM3P9, U47924.2, XIST, Z95115.1, ZFAND2A, ZKSCAN2-DT, ZNF529-AS1 | **AC007128.2, AC012363.2, AC015563.3, AC080037.2, AC116351.2, AL132712.2, BBOX1-AS1, ELFN1-AS1**  AC073046.1, AFAP1-AS1, AL096870.10, SMARCA5-AS1, TAPBPL |
| BART T vs pc T  56 BART only  73 up BART  6 down  2 pc only | **AC004943.2, AC007342.5, AC009065.6, AC009509.2, AC009902.2, AC011445.1, AC012073.1, AC017100.1, AC023509.3, AC024575.2, AC036176.1, AC063960.1, AC073270.2, AC099518.1, AC108488.3, AC115618.1, AC120114.1, AC244033.2, AC244517.4, ADNP-AS1, AL035587.2, AL139289.1, AL353759.1, AL354707.1, AL390198.1, AL450345.2, AL831711.1, ATP2A1-AS1, CCDC84-DT, CKMT2-AS1, CU633906.2, DDN-AS1, DLEU1, DNAAF4-CCPG1, ELOA-AS1, ENTPD1-AS1, FAM198B-AS1, LINC00174, LINC00638, LINC00909, LINC01843, LINC01876, LINC02331, LINC02672, LSAMP-AS1, MRPL23-AS1, NKILA, NRSN2-AS1, SLC5A4-AS1, TAPT1-AS1, TFAP2A-AS1, WARS2-AS1, Z97192.1, ZEB1-AS1, ZNF22-AS1, ZNF337-AS1**  AC004816.1, AC005670.3, AC006213.2, AC007128.2, AC008771.1, AC011468.2, AC011477.2, AC020915.3, AC022075.1, AC025154.2, AC026748.3, AC027644.3, AC064807.1, AC074117.1, AC080037.2, AC136475.3, AC137894.1, AC239868.1, AC244517.6, AC245060.7, AF001548.2, AL109627.1, AL133215.2, AL137785.1, AL354743.2, AP000640.1, AP001816.1, AP002360.2, ARHGAP5-AS1, ARRDC1-AS1, BCYRN1, CAPN10-DT, CASC9, CD2BP2-DT, CEBPA-DT, CTBP1-DT, CU634019.6, DGCR11, DHRS4-AS1, DNAJC3-DT, FP236383.3, FUT8-AS1, JPX,, LINC00205, LINC00659, LINC00847, LINC01006, LINC01011, LINC01106, LINC01145, LINC01184, LINC01278, MAFG-DT, MRPL20-AS1, MZF1-AS1, NNT-AS1, NOP14-AS1, NRAV, PAN3-AS1, PAXIP1-AS2, PCAT7, PPP1R26-AS1, RAB4B-EGLN2, RNASEH1-AS1, SMARCA5-AS1, SREBF2-AS1, TMEM44-AS1, TUG1, UBA6-AS1, VASH1-AS1, VIM-AS1, ZNF503-AS2, ZNF793-AS1 | **AL022328.2, ANKRD10-IT1**  AC073046.1, AC116351.2, AL137058.2, CECR7, DANCR, MIR3648-1 |
| Common AE and BART T vs pc T  Compare individually and find commons | **AC004943.2**, **AC012073.1**, **AC023509.3**, **AC108488.3**, **AC120114.1**, **ADNP-AS1**, **AL354707.1**, **CKMT2-AS1**, **ELOA-AS1**, **LINC00174**, **LINC01843**, **MRPL23-AS1**, **TAPT1-AS1**, **TFAP2A-AS1**, **WARS2-AS1**, **ZEB1-AS1**, AC020915.3, AC074117.1, AC239868.1, AC245060.7, AF001548.2, AL133215.2, AP002360.2, ARRDC1-AS1, BCYRN1, CAPN10-DT, CU634019.6, JPX, LINC00659, LINC01006, LINC01145, MAFG-DT, MRPL20-AS1, NOP14-AS1, PPP1R26-AS1, RAB4B-EGLN2, RNASEH1-AS1, TMEM44-AS1 | AC073046.1, AC116351.2 |

*Bolded are only expressed in AE and/or BART tumors (>0.5 rpkms)

** Bolded are only expressed in pc tumors (>0.5 rpkms)
